# Modeling the Sensory Characteristics of Japanese Sake Using the Sake Metabolome Analysis Method

**DOI:** 10.3390/metabo15080559

**Published:** 2025-08-20

**Authors:** Takuji Kobayashi, Yuko Komatsu-Hata, Ryota Saito, Hisashi Yazawa, Masayuki Takahashi, Ken Oda, Kazuhiro Iwashita

**Affiliations:** National Research Institute of Brewing, 3-7-1 Kagamiyama, Higashi-Hiroshima 739-0046, Hiroshima, Japan

**Keywords:** Japanese sake, metabolome, liquid chromatography mass spectrometry, quantitative descriptive analysis, prediction model, orthogonal projections to latent structures

## Abstract

**Background/Objectives:** The components of food and beverages are important elements that determine their palatability. Although the components of sake, a traditional Japanese alcoholic beverage, have been studied for many years, their correlation with sensory characteristics is unclear. **Methods:** We investigate the correlation with the sake metabolome analysis method developed by our group using ultra-high-performance liquid chromatography quadrupole time-of-flight mass spectrometry. We constructed orthogonal projections to latent structure models to predict sensory evaluation data obtained through the quantitative descriptive analysis method from the sake metabolome data. **Results:** For two years of study, 8 sensory evaluation models of the 2016 brewing year and 11 sensory evaluation models of the 2017 brewing year, including color, ethyl hexanoate, *Hine-ka*, *Nama hine-ka*, ethyl acetate, grainy/sweet aroma, sweetness, sourness, body, astringency, harsh taste/acrid taste, aftertaste, and overall quality, demonstrated a predictive performance with *Q*^2^ > 0.5. Liquid chromatography-based analytical data indicated that it is possible to predict not only taste but also aroma. Additionally, the generalization performance of the prediction models for sensory evaluation attributes common to both years was verified. **Conclusions:** These results provide a new option for explaining the sensory characteristics of sake from its components and contribute to a deeper understanding of them.

## 1. Introduction

For humans, the significance of food and drink extends beyond mere nutritional intake; it also provides the psychological satisfaction derived from the experience of palatability. In particular, alcoholic beverages provide feelings of enjoyment that enrich our life. This enrichment arises not only from the effects of ethanol on the body and mind, but also from the sensory experiences of taste and aroma related to other components. This is clear from the fact that humans do not prefer drinking pure ethanol solutions. Because flavor, composed of taste and aroma, is an essential element of palatability, understanding the correlation between the flavors perceived by humans and the components is important for enjoying food and beverages and for making better products. Recently, due to social factors and changes in consumer preferences, the consumption of alcoholic beverages has declined domestically in Japan. As for sake, a traditional Japanese alcoholic beverage, the taxable volume of sake has decreased from its peak of 1.77 million KL in the fiscal year 1973 to 0.39 million KL in the fiscal year 2023, and the demand for high-value-added products is increasing, and the market outside Japan is expanding [1,2].

Sake is a complex alcoholic beverage composed of numerous components. This is because it is prepared through many processes and parameters, including the raw ingredient rice and the involvement of microorganisms, such as *koji* mold and yeast [3]. The investigation of sake flavor has long been a subject of interest, and the correlations between the components and sensory characteristics of sake have been studied for many years [4,5,6,7]. Sensory evaluation is a basic and essential analytical method for sake, performed not only in laboratories but also in sake breweries. Sensory evaluations, including those conducted at sake awards, such as the Annual Japan Sake Awards, have contributed to the understanding of sensory characteristics and the improvement of sake quality [8]. Many studies have used component analysis to evaluate the components that contribute to the flavor of sake [9]. Based on research findings, the organization of flavor terminology for the sensory analysis of sake and the creation of flavor wheels have been carried out, thereby establishing sensory evaluation methods for sake [10]. Some studies have been conducted to construct models predicting sake flavor from its components using statistical analysis [4]. For example, prediction models have been developed for the sweetness and fullness of sake using the analytical values of reducing sugar as glucose and acidity [11,12]. However, sake contains hundreds of compounds, and understanding its sensory characteristics requires considering their interactions, with the correlation between the components and sensory characteristics of sake not fully elucidated. It is conceivable that some components in sake have not yet been detected. Consequently, current knowledge to explain the palatability of sake is insufficient.

Analytical instruments have evolved over time, and in recent years, metabolomic analysis, which enables the high-sensitivity and comprehensive analysis of components in food and beverages, has been recognized as a powerful tool [13]. In sake studies, the metabolomic analysis has been reported [3,14,15,16,17,18,19]. Shimofuji et al. highlighted the importance of a comprehensive analysis to correlate the physicochemical characteristics of sake with sensory evaluation data in their report [20]. Some studies have linked sake component analysis values to sensory characteristics through metabolomic analysis. For example, capillary electrophoresis (CE)–time-of-flight mass spectrometry (TOF-MS) was used to predict *zatsumi*, sweetness, bitterness, and sourness [14]; two-dimensional gas chromatography (GC)–TOF-MS was used to assess the quality of *ginjo* sake [18], and GC–MS was used to predict *oshi-aji* [21]. Mimura et al. treated sake samples with three sample preparation methods and injected them into GC–MS, detecting nonvolatile and volatile components. They linked the sensory evaluation score obtained using the quantitative descriptive analysis (QDA) with component profiles, constructing orthogonal projections to latent structures (OPLS) models to predict each of the 12 sake attributes and extracting important variables [16]. Thus, the types of sake used for analysis, the methods of component analysis, and the sensory characteristics targeted vary by research, and accumulating research is necessary to analyze the correlation between component analysis data and sensory evaluation results.

In the study of sake components, the correlation with sake-making parameters is important, and analysis using metabolomics technology is being conducted. Recently, Yazawa et al. reported that the rice cultivar, rice polishing ratio, and differences in yeast strains can affect sake metabolites [3]. This was accomplished with the “sake metabolome analysis method” using ultra-high-performance liquid chromatography quadrupole (UPLC-Q)/Tof-MS, characterized by very simple sample pretreatment and a short analysis time of 30 min per sample, capable of detecting low-molecular-weight compounds. For fermented beverages, the raw materials often play a significant role in the final product’s quality (e.g., grapes in wine and barley and hops in beer), and in sake, the variety and quality of rice as raw materials impact sake quality. The effects of microorganisms, such as *koji* mold and yeast, as well as the complexity and variety of the production process, including the differences in sake breweries, significantly influence sake quality. Hence, sake exhibits a diversity of flavor and qualities, and there has been much research on the correlation between the sake-making process and the components of sake. Studies using the sake metabolome analysis method have demonstrated that this correlation can be represented through sake metabolites [3,22]. As reported by CE–MS and GC–MS, other comprehensive analysis results have been linked to sensory evaluation results [14,16,18,21]. However, the data obtained using the sake metabolome analysis method have not been linked to the sensory evaluation results.

In this study, we aimed to construct models to predict sensory evaluation scores from sake metabolome data obtained using the sake metabolome analysis method for sake produced in different sake breweries using various rice cultivars. Furthermore, we conducted analyses across multiple years and aimed to verify the predictive performance of the constructed model. This study provides a new option for explaining the sensory characteristics of sake from its components, contributing to a deeper understanding of these characteristics. By elucidating the correlation between sake components and sensory characteristics, the relationship between the sake-making process and sensory evaluation characteristics also becomes easier to understand. This enables product development and quality control of sake to be carried out more logically and efficiently.

## 2. Materials and Methods

### 2.1. Samples

In this study, 66 bottles of sake, produced in 19 sake breweries across 5 prefectures in Japan during the 2016 brewing year (BY) (July 2016–June 2017) (H28BY, according to the Japanese calendar) and during 2017 BY (July 2017–June 2018) (H29BY), were used (H28BY: 26 bottles and H29BY: 40 bottles) (Table 1). These sake were all made using the rice varieties indicated below, which were made by the sake breweries that cooperated in the study. The rice cultivars, excluding those not disclosed, included *Hyogonishiki*, *Hyogo Sake* 85, *Yumesasara* (*Tochigisake*-27), *Hyakumangokunoshiro* (*Ishikawasake*-68), *Iwai*, and *Kyonokagayaki*, totaling nine varieties (Table 1). The rice polishing ratios were mainly 40–60% (40–60% for H28BY and 35–85% for H29BY). The brewing microorganisms (mainly *koji* mold and yeast) and sake-making parameters were freely determined by individual sake breweries to investigate the sensory characteristics of commercial sake in this study. The sake samples were stored at 4 °C until used for component analysis or sensory evaluation.

### 2.2. Sensory Evaluations

Sensory evaluations were performed using QDA separately for each BY. The panelists were 19 individuals in H28BY and 26 in H29BY who belonged to sake breweries, universities, or public research organizations. They were involved in sake production and research and can perform the sensory evaluation of sake. All panelists received verbal explanations about the handling of information prior to the sensory evaluation and agreed to participate in the study by providing responses to the sensory evaluation. The sake samples were temperature adjusted to 18–20 °C and served in sake tasting cups with concentric blue circles on the bottom [23,24]. The panelists transferred the sake from these cups into plastic cups using a dropper for sensory evaluation.

Prior to the actual sensory evaluation, a preliminary sensory evaluation was performed by several representative panelists, who had expertise in each sake cultivar used in this study. They listed the perceived aromas and tastes of the samples as “words.” These characteristics were shared and discussed by representative panelists to identify those that could be quantitatively expressed, and a 6 cm scale was prepared for the evaluation attributes (Table 2). The actual sensory evaluation began with training the panelists using standard samples for sensory evaluation. The control sake used as the base for these samples was a *daiginjo* sake made by the National Research Institute of Brewing in H28BY (Y-6) and H29BY (Y-p and G-a, respectively), which was charcoal-filtered to primarily reduce *ginjo*-*ka*. Standard samples for sensory evaluation were prepared by adding various compounds to control sake (Tables S1 and S2). For sensory evaluation attributes without standard samples, the panelists set a relative standard for all sake samples during the actual sensory evaluation. The actual sensory evaluation was performed with a request to evaluate at least three rounds in each session to ensure reproducibility.

### 2.3. General Properties and Aroma Components of Sake

Ethanol concentration (alcohol content), acidity, amino acid content, sake meter value (*nihonsyu-do*), and concentration of volatile aromatic compounds (ethyl acetate, *n*-propanol, isobutanol, isoamyl acetate, isoamyl alcohol, and ethyl hexanoate) in sake were determined using the official methods of the National Tax Administration Agency, Japan, and the standard analytical methods of the National Research Institute of Brewing, Japan [25,26] (Table S3). Ethanol concentration was analyzed by GC, amino acid content was analyzed using ethanol with an auto piston burette APB-510 (Kyoto Electronics Manufacturing, Kyoto, Japan), and the sake meter value was analyzed by an oscillation-type density meter. Each sample was analyzed in duplicate (acidity, amino acid content, and sake meter value) or triplicate (ethanol concentration and concentration of volatile aromatic compounds).

### 2.4. Sake Metabolome Analysis

Sake samples were pretreated as described [3] and stored at −30 °C until sake metabolome analysis. Sake samples were filtered using Amicon Ultra 0.5 3K (Merck Millipore, Billerica, MA, USA) and then diluted 10-fold by using MS-grade water. The pretreated samples of H28BY and H29BY were analyzed by UPLC-Q/TOF-MS using a sake metabolome analysis method [3]. Briefly, a column was an Acquity UPLC HSS T3 (2.1 × 150 mm^2^, 1.8 mm column) (Waters, Milford, MA, USA), and mobile phases A and B were 0.1% (*v*/*v*) formic acid in Milli-Q water and 0.1% (*v*/*v*) formic acid in acetonitrile, respectively. The total analysis time per sample is 30 min, including the time until the next sample can be analyzed. Each sample was analyzed in triplicate.

The raw analytical data were processed using MarkerLynx XS in MassLynx V4.1 software (Nihon Waters, Tokyo, Japan) to detect and align the peaks. A peak table containing 8516 peaks was exported. Each peak that had a maximum value of zero or strong variations between the values for all replicates of every sample (maximum coefficient of variation ≥10%) was removed from the peak table. This gave a new peak table containing 430 peaks.

Peaks for metabolites were annotated as candidate compounds by comparing the retention time (RT) and mass-to-charge ratio for each peak to an in-house library of compounds detected by UPLC-Q/TOF-MS [3]. The thresholds of differences in RT and mass-to-charge ratio were 0.1 min and 0.007 Da, respectively.

### 2.5. Statistical Analysis

For the quality check of sensory evaluation data, an outlier removal and analysis of variance were performed using JMP 13.0.0. Initially, box-and-whisker plots were created for each sensory evaluation attribute and sake sample, identifying and removing outliers that fell below the lower whisker or exceeded the upper whisker. Subsequently, one-way analysis of variance was performed to test for differences in the mean scores of the sensory evaluation attributes (*p* < 0.05).

Principal component analysis (PCA) and OPLS analysis were performed using SIMCA ver. 17. The analytical and sensory evaluation data were standardized using UV scaling (autoscaling, mean = 0, and variance = 1). PCA was performed using mean values. OPLS analysis was performed using sensory evaluation data as objective variables and analytical data as explanatory variables. Using data with *n* = 3, a 7-fold cross-validation was performed to ensure that the same sample did not enter different cross-validation groups, adopting the maximum number of latent variables as *Q*^2^ increased. Then, a permutation test (*n* = 100) was performed, reducing latent variables that satisfied *R*^2^ < 0.3 and *Q*^2^ < −0.05 [27,28]. The important variables for each sensory evaluation score prediction model were selected based on those with high variable importance for prediction predictive (VIPpred) values. A correlation analysis was performed using Excel 2016.

## 3. Results

### 3.1. Sensory Evaluation of Sake Using QDA

Sake is made through a complex multistep process; therefore, not only the variety and quality of rice but also the rice polishing ratio, brewing microorganisms, such as *koji* mold and yeast, and differences in sake-making methods affect the quality of the final product. The sake samples used in this study, as described in the Materials and Methods section, were made with various rice cultivars and sake-making methods, with rice polishing ratios of mainly 40–60%, resulting in a relatively diverse range of sake flavors and qualities (Table 1).

**Figure 1:**
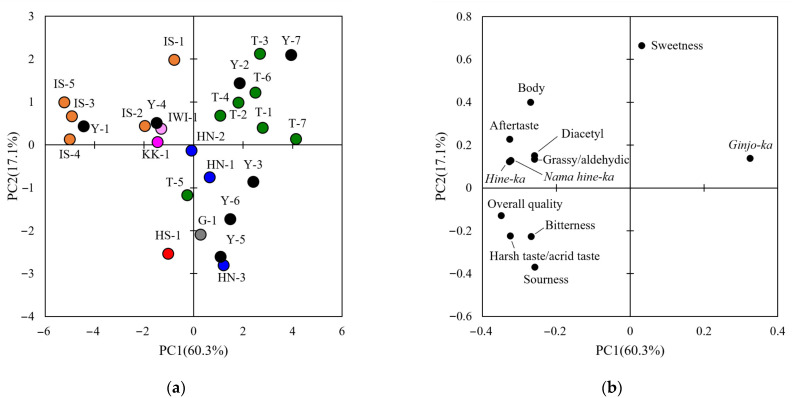
Principal component analysis of the sensory evaluation data for H28BY. (**a**) A score scatter plot of PC1 against PC2 for the sensory evaluation data in H28BY. Data for different sake samples are shown, and different colors indicate different rice cultivars. Numbers indicate the sample IDs of the sake samples (Table 1). (**b**) Loading plot of the sensory evaluation data in H28BY.

**Figure 2:**
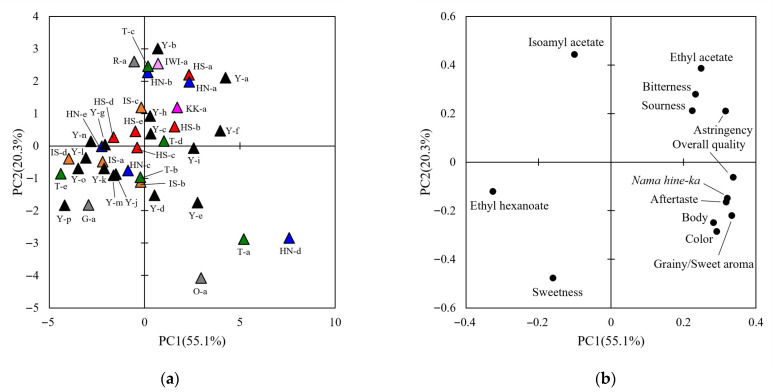
Principal component analysis of the sensory evaluation data for H29BY. (**a**) A score scatter plot of PC1 against PC2 for the sensory evaluation data in H29BY. Data for different sake samples are shown, and different colors indicate different rice cultivars. Numbers indicate the sample IDs of the sake samples (Table 1). (**b**) Loading plot of the sensory evaluation data in H29BY.

Sensory evaluations of the sake samples were performed for each BY, as described in the Materials and Methods section. For each year, 13 attributes of the sensory evaluation were obtained (Table S4). To determine the sensory evaluation attributes for subsequent analysis, a one-way analysis of variance was performed. This analysis showed significant differences between samples in 12 attributes for H28BY, excluding fatty acid smell, and in all attributes for H29BY (Table 3). Subsequently, a PCA was performed to overview the sensory evaluation results (Figure 1 and Figure 2). In H28BY, the explained variance ratios of PC1 and PC2 were 60.3% and 17.1%, respectively, showing a tendency for plots to gather according to the rice cultivar. Specifically, *Yumesasara* was positioned from the center to the first quadrant, *Ishikawasake*-68 or sake from Ishikawa Prefecture was in the second quadrant, and *Hyogonishiki* was positioned from the center to the fourth quadrant. *Yamadanishiki* was widely distributed, indicating not only the influence of rice cultivars but also the sake breweries. In H29BY, the explained variance ratio of PC1 and PC2 were 55.1% and 20.3%, respectively, showing less gathering according to the rice cultivars and sake breweries compared with H28BY. Loading plots showed common features across both years (Figure 1 and Figure 2). Bitterness and sourness were in one direction; body, aftertaste, and *nama hine*-*ka* were in another direction; and sweetness was in the other direction. Although components, such as ethyl hexanoate and isoamyl acetate, were part of *ginjo*-*ka*, they were in a different group in H29BY. These results suggest that the sake samples used in this study exhibited diverse qualities in each BY, as indicated by the sensory evaluations.

### 3.2. General and Comprehensive Analyses of Sake

**Figure 3:**
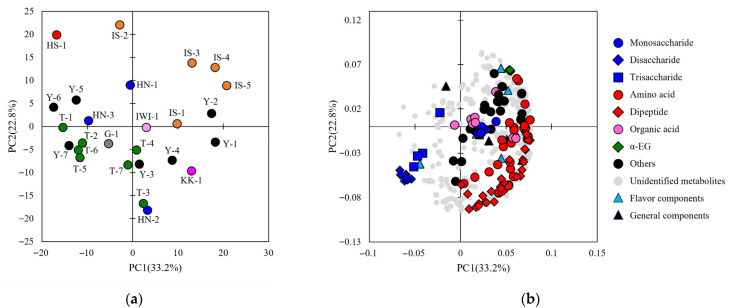
Principal component analysis of the metabolome, general properties, and aroma components of H28BY. (**a**) A score scatter plot of PC1 against PC2 for the integrated data of general properties, aroma components, and metabolome data in H28BY. Data for different sake samples are shown, and different colors indicate different rice cultivars. Numbers indicate the sample IDs of the sake samples (Table 1). (**b**) Loading plot of the metabolome data in H28BY. Different symbols are for peaks that matched the indicated compound groups in the in-house standard library or for analysis items.

**Figure 4:**
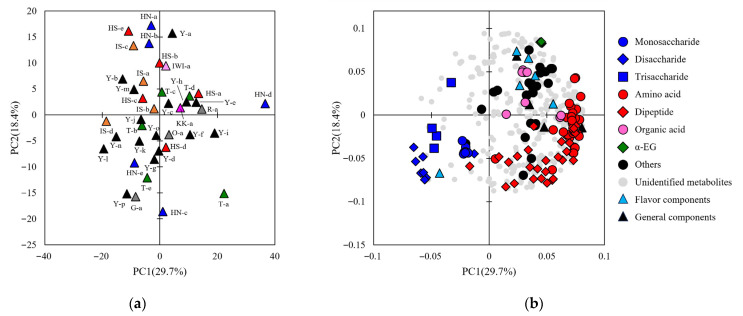
Principal component analysis of the metabolome, general properties, and aroma components of H29BY. (**a**) A score scatter plot of PC1 against PC2 for the integrated data of general properties, aroma components, and metabolome data in H29BY. Data for different sake samples are shown, and different colors indicate different rice cultivars. Numbers indicate the sample IDs of the sake samples (Table 1). (**b**) Loading plot of metabolome data in H29BY. The different symbols are for peaks that matched the indicated compound groups in the in-house standard library or for analysis items.

The analysis of the sake samples resulted in data for four general properties, six aroma components, and 430 peaks from the metabolome analysis (Tables S3 and S5). Of the metabolome peaks, 119 matched the RT and mass-to-charge ratio in the in-house library and were annotated as candidate compounds. The annotated peaks included sugars (monosaccharides, disaccharides, and trisaccharides), amino acids, dipeptides, organic acids, sugar alcohols, amines, alcohols, and esters (Table S5). To obtain an overview of the analytical data, a PCA was performed using the integrated data of the general properties, aroma components, and metabolome peaks (Figure 3 and Figure 4). In H28BY, the explained variance ratios of PC1 and PC2 were 33.2% and 22.8%, respectively, and in H29BY, they were 29.7% and 18.4%, respectively. The influence of rice cultivars and sake breweries was observed, resembling the results of a QDA plot. The similarity between the plots of analytical values and sensory evaluations suggests that the analytical data reflects the sensory evaluation results. The loading plots showed a consistent distribution across both years, with amino acids and peptides widely spread in one direction, forming a semicircle, whereas sugars (monosaccharides, disaccharides, and trisaccharides) were distributed in a separate group (Figure 3 and Figure 4). When the PCA was performed on the analytical data of H28BY and H29BY samples together, no bias was observed between the years, suggesting that the data were consistent across years (Figure S1). These results imply that the values obtained from both general and comprehensive analyses are related to sensory evaluation results.

### 3.3. Sake Metabolome Data Can Predict Sensory Evaluation Scores

Initially, to construct models predicting sensory evaluation scores only from the sake metabolome data, an OPLS analysis was performed with 430 peaks of sake metabolome data as explanatory variables and each sensory evaluation score as the objective variable. The number of latent variables, a hyperparameter in OPLS analysis, was determined as described in the Materials and Methods section. Models with *R*^2^ > 0.8 were constructed for seven attributes in H28BY and six attributes in H29BY (Table 4). The simulated goodness of fit for unknown data estimated through cross-validation (denoted *Q*^2^) is considered good if *Q*^2^ > 0.5 [29]. Models with *Q*^2^ > 0.5 were established for eight attributes in H28BY and eleven attributes in H29BY (Table 4). These findings indicate that constructing models that predict the sensory evaluation scores of sake aromas and tastes is possible from sake metabolome data. Notably, not only tastes, such as sweetness or body, but also aromas, such as *ginjo*-*ka* and *nama hine*-*ka*, can be predicted from liquid chromatography-based sake metabolome data.

### 3.4. Investigating the Correlation Between Sake Components and Sensory Evaluation Attributes

To investigate the important variables for each sensory evaluation attribute, VIPpred was calculated. Initially, to compare with general analytical items, prediction models for each sensory evaluation attribute were constructed using 440 variables, which included general properties (four items) and aroma components (six compounds), in addition to sake metabolome data (430 peaks). The models constructed using 440 variables showed a similar predictive performance to those constructed using only sake metabolome data (Table S6). Focusing on the VIPpred value of general and aroma components, ethyl hexanoate (F-6), one of the main components of *ginjo*-*ka*, had a VIPpred of >1.5 in both H28BY *ginjo*-*ka* and H29BY ethyl hexanoate, ranking high at 1 and 10, respectively (Tables S7 and S8). The total amount of free acids measured by alkaline neutralization titration, denoted as acidity (G-2), had a VIPpred of >1.5 in both H28BY and H29BY, ranking one as an explanatory variable (Tables S7 and S8). These results indicate that known correlations between components and sensory evaluation attributes were extracted, suggesting that predictive model construction is an adequate method to investigate the correlation between sake components and sensory evaluation attributes.

Next, VIPpred was calculated using the predictive model constructed solely from sake metabolome data. The number of extracted peaks with a relatively high VIPpred of >1.5 was 39–73 in H28BY and 43–80 in H29BY (Table 4). To investigate the correlation between the extracted peaks and each sensory evaluation attribute in detail, the correlation coefficients between the peaks and sensory evaluation scores were calculated, and their plus or minus signs were applied to VIPpred (Table S9). Moreover, the top VIPpred variables, along with their correlation coefficients and annotations, were listed for each model (Table S10). The results showed that monosaccharides containing glucose had a signed VIPpred value > +1.5 for sweetness in both H28BY and H29BY (Table S9), suggesting a positive involvement with sweetness, which is consistent with known correlations. Furthermore, the α-EG showing sweetness and bitterness had a signed VIPpred of >+1.5 for bitterness in both H28BY and H29BY (Table S9), indicating a positive involvement with bitterness, which is consistent with known correlations [30]. The results that known components related to sensory evaluation attributes were selected suggest that peaks with VIPpred values of >1.5 contain compounds directly affecting sensory evaluation attributes. Although indirect or unrelated effects need to be considered, the information from the prediction model suggests a correlation between sake components and sensory evaluation attributes.

### 3.5. Comparison Between the Prediction Models of Different Sensory Evaluation Data

To investigate the generalizability of the prediction models, sensory evaluation data from a year not used in constructing the model were predicted using the model as a validation set. The model constructed with H28BY data was used to predict the sensory evaluation scores for H29BY using the sake metabolome data of H29BY, and its generalization performance was evaluated. Conversely, the same approach was applied using the model built with the H29BY data. This evaluation of the generalization performance was performed for sensory evaluation attributes that shared terms across both years, with H28BY *ginjo*-*ka* corresponding to H29BY ethyl hexanoate and isoamyl acetate, respectively, because *ginjo-ka* is a complex aroma composed of various components including ethyl hexanoate, isoamyl acetate, and other esters and alcohols (Table 5). Predictions using the H28BY model for the H29BY data resulted in three attributes with *R*^2^ > 0.5. Similarly, predictions using the H29BY model for the H28BY data resulted in three attributes with *R*^2^ > 0.5.

To examine the generalization performance, variable selection was performed, selecting peaks common to both years with an absolute value of VIPpred >1.5 (Table 5 and Table S9). Because there were no common variables between the prediction models for H28BY *ginjo*-*ka* and H29BY isoamyl acetate, these were excluded from the subsequent trial. For other combinations, 12–35 peaks were commonly selected with an absolute value of VIPpred >1.5 for both years. Subsequently, prediction models were constructed using the selected variables (Table 5). Models with *Q*^2^ > 0.5 consisted of six attributes for H28BY and five attributes for H29BY, which was the same or slightly less than when all peaks were used (H28BY: six attributes and H29BY: seven attributes). The number of attributes with *R*^2^ > 0.5 in the validation set was four for H28BY and five for H29BY, which was slightly more than when all the peaks were used (H28BY: three attributes and H29BY: three attributes). These results suggest that selecting variables with consistently high VIPpred values across different sensory evaluation data sets allows for the construction of prediction models with equivalent or better generalization performance using substantially fewer variables—less than one-tenth of the original number.

## 4. Discussion

In this study, sensory evaluations were performed over two years using QDA on Japanese sake samples made from various rice cultivars at different sake breweries. In this study, the panelists were 19 individuals in H28BY and 26 in H29BY who were involved in sake production and research and can perform the sensory evaluation of sake. The data is considered to have sufficient reliability for statistical analysis since 10–12 panelists are recommended for QDA [31]. The sake samples were also analyzed using the sake metabolome analysis method to obtain metabolome data. Prediction models predicting sensory evaluation scores from the obtained metabolome data were constructed using an OPLS analysis, resulting in good models (*Q*^2^ > 0.5) for 8 attributes in H28BY and 11 attributes in H29BY (Table 4). Similarly, in other studies where prediction models were constructed for sensory evaluation attributes using QDA data, component data were obtained using GC–MS, with an analysis performed under three pretreatment and analysis conditions [16]. This method detected many nonvolatile and volatile components, totaling 244, but required specific pretreatments for each, with an analysis time of 25–105 min. The sake metabolome analysis method used in this study is a comprehensive nontargeted method using UPLC-Q–TOF-MS, which can be performed with a single simple pretreatment. It is a high-throughput method, taking only 30 min per sample, making it particularly useful in cases with a large number of samples. This method was recently applied to predict the sake brewing characteristics of rice using brown rice metabolome derived from extracts of brown rice, suggesting a wide range of potential applications [32].

The sake metabolome analysis method, despite being based on liquid chromatography, could predict not only taste-related sensory evaluation attributes, such as sweetness, but also aroma-related attributes, such as *ginjo*-*ka* and *nama hine*-*ka* (Table 4). In the sake metabolome analysis method, compound identification was possible through matching with an in-house library, with up to 296 compounds identified. In addition, it was capable of detecting volatile components, such as ethyl hexanoate and isoamyl acetate. However, the peak table used in this study did not include the major components of *ginjo*-*ka*, such as ethyl hexanoate or isoamyl acetate (Table S5). According to the comparison with the in-house library, isoamyl acetate was below the detection limit, while ethyl hexanoate is detectable at concentrations of at least 1 ppm or more, but it was likely not detected due to limitations in the peak picking process. This indicates that constructing prediction models for the sensory evaluation score using data from the sake metabolome analysis method is possible even without the peaks derived from the compounds that are major contributors to sensory evaluation attributes. The reason for this possibility might be that aroma components, such as ethyl hexanoate and isoamyl acetate, are produced through the metabolic pathways of yeast, leading to variations in related compounds, such as precursors, intermediates, or byproducts, with the synthesis of these components. Therefore, even if the specific components are not detected, the behavior of the related compounds can be reflected in the prediction model, enabling prediction. For example, the production of isoamyl acetate can be inhibited by unsaturated fatty acids, and the glucose concentration of sake mash is related to the production of ethyl hexanoate [33,34,35].

The model constructed in this study consists of not only annotated compounds related to sensory evaluation attributes but also provides information on compounds whose correlations have not been reported. For example, in the bitterness prediction model, α-EG was extracted with a VIPpred of >1.5 for both years (Table S9), showing a positive correlation with bitterness. The α-EG exhibits a sweet with a late bitterness taste [30]. In total, 15 extracted variables positively correlated with bitterness, which could be contributing to bitterness in sake. However, it is unclear whether the extracted variables directly affect specific aromas or tastes. Further investigation is needed to identify these compounds as potential contributors to taste and aroma. The list of extracted variables includes unknown peaks not matched with the in-house library. The sake metabolome analysis method used in this study employed a Q/TOF detection system, which provides RT and accurate mass information for peaks derived from compounds not included in the in-house library. Although the correlation between these unknown peaks and sensory evaluation attributes cannot be established, accumulating information on components not previously confirmed in sake represents valuable data, indicating the future significance and utility of this analytical method.

The performance of the prediction model constructed in this study (*R*^2^ = 0.45–0.99, *Q*^2^ = 0.25–0.83) (Table 4) showed comparable *R*^2^ values but lower *Q*^2^ values relative to another study using a comprehensive analysis (*R*^2^ = 0.96–0.98, *Q*^2^ = 0.91–0.96) [16]. There are three potential reasons for this.

Regarding the samples, in the other study, 40 samples of specially designated sake made from *Yamadanishiki*, with a rice polishing ratio of 35–70%, and ordinary sake were used, covering 13 prefectures as the sake-making area [16]. In this study, a specially designated sake made from different rice varieties was used, with 26 samples from five prefectures in H28BY (rice polishing ratio: 40–60%) and 40 samples from five prefectures in H29BY (rice polishing ratio: 35–85%) (Table 1). The two studies differed at least in terms of rice cultivars, the range of rice polishing ratios, and variety of sake breweries. The variety of samples can affect the ease of constructing prediction models. These differences might have contributed to variances in the predictive performance.

Regarding the analytical method, this study used UPLC-Q/TOF-MS, resulting in the use of 430 peaks (Table S5). By contrast, the other study used GC–MS, employing 86 nonvolatile components and 158 volatile components [16]. The explanatory variables used in this study were approximately twice the number used in the other study, which could have influenced the generalization performance of the prediction model. Both data sets included nonvolatile and volatile compounds, but due to the nature of each method, they are inclined to detect different types of compounds. This difference in compound types might have impacted the predictive performance. Determining the appropriate combination of sensory evaluation attributes and component analysis methods remains a challenge for future research.

Finally, regarding the model construction method, in machine learning, the predictive performance can be affected by hyperparameter tuning and the cross-validation method. In this study and the other study, the approaches used to determine latent variables in OPLS analysis and the methods of cross-validation may not have been the same, which could have led to differences in the predictive performance.

In this study, the generalization performance of the prediction models for sensory evaluation attributes was more strictly verified. First, the generalization performance of the prediction model was evaluated using *Q*^2^, which indicates the model’s fit to unknown data through cross-validation within the model. Additionally, the *R*^2^ of the validation set was calculated using sensory evaluation data from different years as unknown data, providing an additional assessment (Table 5). The results mostly showed a lower *R*^2^ of the validation set compared with *Q*^2^, suggesting that the *R*^2^ of the validation set might be a stricter measure for evaluating the generalization performance (Table 5). One possible reason for the lower generalization performance, as indicated by the *R*^2^ of the validation set, could be the data used. The sensory evaluation data from different years used in this study were independently performed each year, and there was no standardization of scales across years, even for the same sensory evaluation attributes. In this study, variable selection was performed to improve generalization performance. The results showed that a prediction model with an equivalent or better generalization performance could be constructed using significantly fewer variables—less than one-tenth of the original number (Table 5). Appropriate variable selection may lead to the construction of more practical prediction models, but determining the optimal method for variable selection remains a future challenge.

To clarify the correlation between the components of sake and its sensory characteristics, several problems must be solved. First, the number and variety of sake samples in the studies, including this study, are limited. This necessitates trials with diverse combinations and larger scales of sake samples to gain a more comprehensive understanding of the sensory characteristics of sake. Second, although the model construction employed a method commonly used in food metabolomics, attempting other machine learning methods containing more complex algorithms might provide additional insights. Shimofuji et al. reported that machine learning is not inherently superior to linear regression for estimating the components of *Junmai Ginjo*-*shu* and various explanatory variables, and regression analysis methods must be considered [36]. Lastly, the sake metabolome analysis method can perform a rapid and comprehensive metabolite analysis due to high-resolution TOF-MS. However, given that GC-based methods are often more suitable for analyzing volatile components, the time-consuming nature of the existing methods suggests a need for more high-throughput and comprehensive techniques to analyze volatile components.

## 5. Conclusions

In this study, prediction models were constructed to predict sensory evaluation scores obtained through a QDA from component analysis data of sake made not in laboratories but in sake breweries using various rice cultivars. Component analysis data were acquired not only through conventional sake analysis methods but also through a high-throughput and comprehensive analysis using the sake metabolome analysis method. The study showed that it was possible to construct prediction models even in the absence of primary aroma components, indicating the utility of this approach in understanding the sensory characteristics of sake. The important variables extracted through model construction provided information about both known and unknown components related to the sensory evaluation attributes and information about unidentified peaks. Comparing prediction models from different years allowed for the verification of the accuracy in predicting sensory evaluation scores. These results provide a new option for explaining the sensory characteristics of sake from its components and contribute to a deeper understanding. By elucidating the correlation between sake components and sensory characteristics, the relationship between the sake-making process and sensory evaluation characteristics also becomes easier to understand. This enables product development and quality control of sake to be carried out more logically and efficiently.

## Figures and Tables

**Table 1:** Sake samples used in this study.

No.	Sample ID	BY	Rice Cultivar	Rice Polishing Ratio (%)	Sake Brewery
1	Y-1	H28/2016	*Yamadanishiki*	60	Ishikawa-A
2	Y-2	H28/2016	*Yamadanishiki*	60	Hyogo-A
3	Y-3	H28/2016	*Yamadanishiki*	50	Hyogo-B
4	Y-4	H28/2016	*Yamadanishiki*	60	Kyoto-A
5	T-1	H28/2016	*Yumesasara* (*Tochigisake*-27)		Tochigi-A
6	T-2	H28/2016	*Yumesasara* (*Tochigisake*-27)		Tochigi-B
7	T-3	H28/2016	*Yumesasara* (*Tochigisake*-27)		Tochigi-C
8	T-4	H28/2016	*Yumesasara* (*Tochigisake*-27)	40	Tochigi-D
9	T-5	H28/2016	*Yumesasara* (*Tochigisake*-27)		Tochigi-E
10	T-6	H28/2016	*Yumesasara* (*Tochigisake*-27)	40	Tochigi-F
11	IS-1	H28/2016	*Ishikawasake*-68	50	Ishikawa-A
12	IS-2	H28/2016	*Ishikawasake*-68	40	Ishikawa-B
13	IS-3	H28/2016	*Ishikawasake*-68	50	Ishikawa-B
14	IS-4	H28/2016	*Ishikawasake*-68	50	Ishikawa-B
15	IS-5	H28/2016	*Ishikawasake*-68	50	Ishikawa-B
16	IWI-1	H28/2016	*Iwai*	60	Kyoto-A
17	KK-1	H28/2016	*Kyonokagayaki*	60	Kyoto-A
18	HN-1	H28/2016	*Hyogonishiki*	60	Hyogo-A
19	HN-2	H28/2016	*Hyogonishiki*	55	Hyogo-B
20	Y-5	H28/2016	*Yamadanishiki*	40	Hiroshima-A
21	HN-3	H28/2016	*Hyogonishiki*	40	Hiroshima-A
22	HS-1	H28/2016	*Hyogo Sake* 85	40	Hiroshima-A
23	G-1	H28/2016	*Ginnosato*	40	Hiroshima-A
24	Y-6	H28/2016	*Yamadanishiki*	40	Hiroshima-A
25	Y-7	H28/2016	*Yamadanishiki*	40	Hiroshima-A
26	T-7	H28/2016	*Yumesasara* (*Tochigisake*-27)	40	Hiroshima-A
27	HN-a	H29/2017	*Hyogonishiki*	70	Hyogo-C
28	Y-a	H29/2017	*Yamadanishiki*	60	Hyogo-C
29	IS-a	H29/2017	*Ishikawasake*-68		Ishikawa-C
30	Y-b	H29/2017	*Yamadanishiki*	50	Ishikawa-C
31	IS-b	H29/2017	*Ishikawasake*-68	50	Ishikawa-A
32	Y-c	H29/2017	*Yamadanishiki*	50	Ishikawa-A
33	IS-c	H29/2017	*Ishikawasake*-68	40	Ishikawa-B
34	Y-d	H29/2017	*Yamadanishiki*	40	Ishikawa-B
35	HN-b	H29/2017	*Hyogonishiki*	60	Hyogo-A
36	Y-e	H29/2017	*Yamadanishiki*	60	Hyogo-A
37	IWI-a	H29/2017	*Iwai*	60	Kyoto-A
38	KK-a	H29/2017	*Kyonokagayaki*	60	Kyoto-A
39	Y-f	H29/2017	*Yamadanishiki*	60	Kyoto-A
40	HS-a	H29/2017	*Hyogo Sake* 85	63	Hyogo-D
41	R-a	H29/2017	(*undisclosed*)	63	Hyogo-D
42	HN-c	H29/2017	*Hyogonishiki*	50	Hyogo-B
43	Y-g	H29/2017	*Yamadanishiki*	50	Hyogo-B
44	HS-b	H29/2017	*Hyogo Sake* 85	60	Hyogo-E
45	Y-h	H29/2017	*Yamadanishiki*	60	Hyogo-E
46	HN-d	H29/2017	*Hyogonishiki*	55	Hyogo-F
47	Y-i	H29/2017	*Yamadanishiki*	55	Hyogo-F
48	HS-c	H29/2017	*Hyogo Sake* 85	65	Hyogo-G
49	Y-j	H29/2017	*Yamadanishiki*	55	Hyogo-G
50	T-a	H29/2017	*Yumesasara* (*Tochigisake*-27)	55	Tochigi-G
51	O-a	H29/2017	*Omachi*	50	Tochigi-G
52	T-b	H29/2017	*Yumesasara* (*Tochigisake*-27)	40	Tochigi-D
53	Y-k	H29/2017	*Yamadanishiki*	40	Tochigi-D
54	T-c	H29/2017	*Yumesasara* (*Tochigisake*-27)	62	Tochigi-E
55	Y-l	H29/2017	*Yamadanishiki*	40	Tochigi-E
56	T-d	H29/2017	*Yumesasara* (*Tochigisake*-27)	55	Tochigi-B
57	Y-m	H29/2017	*Yamadanishiki*	43	Tochigi-B
58	Y-n	H29/2017	*Yamadanishiki*	40	Hiroshima-A
59	Y-o	H29/2017	*Yamadanishiki*	60	Hiroshima-A
60	HS-d	H29/2017	*Hyogo Sake* 85	60	Hiroshima-A
61	HS-e	H29/2017	*Hyogo Sake* 85	85	Hiroshima-A
62	IS-d	H29/2017	*Ishikawasake*-68	40	Hiroshima-A
63	Y-p	H29/2017	*Yamadanishiki*	40	Hiroshima-A
64	G-a	H29/2017	*Ginnosato*	35	Hiroshima-A
65	T-e	H29/2017	*Yumesasara* (*Tochigisake*-27)	40	Hiroshima-A
66	HN-e	H29/2017	*Hyogonishiki*	40	Hiroshima-A

**Table 2:** Evaluation attributes of sensory evaluation.

Evaluation Attributes	Score		
	H28BY	H29BY	
Appearance			
Color	-	0–5	(colorless–deep)
Odor			
* Ginjo-ka*	0–5	-	(none–strong)
Ethyl hexanoate	-	0–5	(none–strong)
Isoamyl acetate	-	0–5	(none–strong)
* Hine-ka*	0–5	-	(none–strong)
* Nama hine-ka*	0–5	0–5	(none–strong)
Ethyl acetate	-	0–5	(none–strong)
Fatty acid smell	0–5	-	(none–strong)
Grassy/aldehydic	0–5	-	(none–strong)
Diacetyl	0–5	-	(none–strong)
Grainy/sweet aroma	-	0–5	(none–strong)
Taste			
Sweetness	0–5	0–5	(none–strong)
Sourness	0–5	0–5	(none–strong)
Body	0–5	0–5	(thin–thick)
Bitterness	0–5	0–5	(none–strong)
Astringency	-	0–5	(none–strong)
Harsh taste/acrid taste	0–5	-	(none–strong)
Aftertaste	0–5	0–5	(light–heavy)
Overall quality	1–5	1–5	(excellent–faulty)

**Table 3:** Analysis of variance results of the sensory evaluation data.

Evaluation Attributes of Sensory Test	H28BY		H29BY	
	*F*-Value	*p*-Value	*F*-Value	*p*-Value
Appearance				
Color	-	-	24.5811	<0.0001
Odor				
* Ginjo-ka*	5.9915	<0.0001	-	-
Ethyl hexanoate	-	-	18.065	<0.0001
Isoamyl acetate	-	-	2.4013	<0.0001
* Hine-ka*	6.5311	<0.0001	-	-
* Nama hine-ka*	16.6516	<0.0001	9.1929	<0.0001
Ethyl acetate	-	-	4.1869	<0.0001
Fatty acid smell	1.0472	0.4016	-	-
Grassy/aldehydic	3.7718	<0.0001	-	-
Diacetyl	2.6687	<0.0001	-	-
Grainy/sweet aroma	-	-	7.653	<0.0001
Taste				
Sweetness	7.2706	<0.0001	9.4732	<0.0001
Sourness	3.4983	<0.0001	7.8414	<0.0001
Body	6.3101	<0.0001	6.519	<0.0001
Bitterness	1.6625	0.0205	4.0764	<0.0001
Astringency	-	-	2.8253	<0.0001
Harsh taste/acrid taste	2.6811	<0.0001	-	-
Aftertaste	4.2497	<0.0001	5.6017	<0.0001
Overall quality	9.5073	<0.0001	16.7304	<0.0001

**Table 4:** Results of modeling the sensory evaluation attributes.

Evaluation Attributes of Sensory Test	H28BY						H29BY					
	No. of Latent Variable	*R* ^2^	RMSE	*Q* ^2^	No. of Selected Variable	CV-ANOVA *p*-Value	No. of Latent Variable	*R* ^2^	RMSE	*Q* ^2^	No. of Selected Variable	CV-ANOVA *p*-Value
Color	1 + 3 + 0	0.876	0.24	0.672	61		1 + 4 + 0	0.919	0.20	0.691	74	1.7 × 10^−23^
*Ginjo-ka*						4.9 × 10^−14^						
Ethyl hexanoate							1 + 2 + 0	0.834	0.33	0.712	60	2.4 × 10^−28^
Isoamyl acetate							1 + 3 + 0	0.768	0.15	0.371	60	1.2 × 10^−8^
*Hine-ka*	1 + 4 + 0	0.965	0.09	0.800	62	1.0 × 10^−19^						
*Nama hine-ka*	1 + 5 + 0	0.990	0.09	0.834	73	9.5 × 10^−21^	1 + 2 + 0	0.826	0.27	0.646	50	2.4 × 10^−23^
Ethyl acetate							1 + 1 + 0	0.637	0.25	0.511	71	4.1 × 10^−17^
Fatty acid smell	-	-	-	-	-	-						
Grassy/aldehydic	1 + 2 + 0	0.697	0.26	0.252	56	1.7 × 10^−3^						
Diacetyl	1 + 0 + 0	0.508	0.19	0.389	70	9.7 × 10^−9^						
Grainy/sweet aroma							1 + 3 + 0	0.866	0.22	0.624	68	2.2 × 10^−20^
Sweetness	1 + 4 + 0	0.950	0.14	0.682	52	3.3 × 10^−13^	1 + 1 + 0	0.747	0.26	0.659	80	5.4 × 10^−26^
Sourness	1 + 5 + 0	0.959	0.09	0.675	73	9.5 × 10^−12^	1 + 6 + 0	0.917	0.14	0.594	52	5.6 × 10^−15^
Body	1 + 0 + 0	0.690	0.34	0.615	40	2.9 × 10^−16^	1 + 1 + 0	0.758	0.22	0.514	64	2.8 × 10^−17^
Bitterness	1 + 0 + 0	0.450	0.20	0.258	49	1.4 × 10^−5^	1 + 0 + 0	0.535	0.24	0.421	50	1.3 × 10^−14^
Astringency							1 + 0 + 0	0.585	0.21	0.508	43	9.7 × 10^−19^
Harsh taste/acrid taste	1 + 5 + 0	0.950	0.10	0.610	65	2.3 × 10^−9^						
Aftertaste	1 + 0 + 0	0.545	0.36	0.437	39	4.5 × 10^−10^	1 + 0 + 0	0.640	0.26	0.522	69	1.7 × 10^−19^
Overall quality	1 + 3 + 0	0.917	0.18	0.759	70	1.6 × 10^−18^	1 + 3 + 0	0.859	0.22	0.648	45	5.9 × 10^−22^

**Table 5:** Evaluation of the sensory evaluation models between brewing years.

Evaluation Attributes of Sensory Test		No. of Selected Variables in Both Years (Vippred > 1.5)	H28BY							H29BY						
For Model Construction	For Model Validation		No. of Latent Variables	*R*^2^ of Calibration Set	RMSE of Calibration Set	*Q*^2^ of Calibration Set	*R*^2^ of Validation Set	RMSE of Validation Set	CV-ANOVA *p*-Value	No. of Latent Variables	*R*^2^ of Calibration Set	RMSE of Calibration Set	*Q*^2^ of Calibration Set	*R*^2^ of Validation Set	RMSE of Validation Set	CV-ANOVA *p*-Value
H28BY *Ginjo-ka* *	H29BY Ethyl hexanoate	35	1 + 3 + 0	0.876	0.24	0.672	0.583	0.53	4.9 × 10^−14^	1 + 0 + 0	0.620	0.42	0.588	0.647	0.50	3.5 × 10^−15^
H28BY *Ginjo-ka* *	H29BY Isoamyl acetate	0	1 + 3 + 0	0.876	0.24	0.672	0.063	0.92	4.9 × 10^−14^	-	-	-	-	-	-	-
H28BY *Nama hine-ka*	H29BY *Nama hine-ka*	23	1 + 5 + 0	0.990	0.09	0.834	0.265	0.63	9.5 × 10^−21^	1 + 0 + 0	0.731	0.44	0.688	0.696	0.40	1.0 × 10^−19^
H28BY Sweetness	H29BY Sweetness	31	1 + 4 + 0	0.950	0.14	0.682	0.317	0.46	3.3 × 10^−13^	1 + 0 + 0	0.557	0.39	0.520	0.491	0.44	1.1 × 10^−12^
H28BY Sourness	H29BY Sourness	12	1 + 5 + 0	0.959	0.09	0.675	0.368	0.44	9.5 × 10^−12^	1 + 0 + 0	0.362	0.34	0.325	0.526	0.34	3.9 × 10^−7^
H28BY Body	H29BY Body	21	1 + 0 + 0	0.690	0.34	0.615	0.583	0.39	2.9 × 10^−16^	1 + 2 + 0	0.849	0.24	0.629	0.404	0.67	3.3 × 10^−14^
H28BY Bitterness	H29BY Bitterness	29	1 + 0 + 0	0.450	0.20	0.258	0.477	0.38	1.4 × 10^−5^	1 + 1 + 0	0.617	0.17	0.388	0.154	0.46	2.4 × 10^−8^
H28BY Aftertaste	H29BY Aftertaste	19	1 + 0 + 0	0.545	0.36	0.437	0.591	0.28	4.5 × 10^−10^	1 + 2 + 0	0.822	0.23	0.647	0.362	0.71	4.3 × 10^−10^
H28BY Overall quality	H29BY Overall quality	18	1 + 3 + 0	0.917	0.18	0.759	0.359	0.57	1.6 × 10^−18^	1 + 0 + 0	0.683	0.35	0.654	0.648	0.48	5.2 × 10^−18^
H29BY Ethyl hexanoate	H28BY *Ginjo-ka* *	35	1 + 2 + 0	0.834	0.33	0.712	0.539	0.50	2.4 × 10^−28^	1 + 1 + 0	0.748	0.41	0.700	0.592	0.47	3.3 × 10^−29^
H29BY Isoamyl acetate	H28BY *Ginjo-ka* *	0	1 + 3 + 0	0.768	0.15	0.371	0.001	0.77	1.2 × 10^−8^	-	-	-	-	-	-	-
H29BY *Nama hine-ka*	H28BY *Nama hine-ka*	23	1 + 2 + 0	0.826	0.27	0.646	0.558	0.56	2.4 × 10^−23^	1 + 0 + 0	0.702	0.34	0.688	0.731	0.47	2.5 × 10^−30^
H29BY Sweetness	H28BY Sweetness	31	1 + 1 + 0	0.747	0.26	0.659	0.387	0.53	5.4 × 10^−26^	1 + 0 + 0	0.505	0.36	0.474	0.558	0.46	4.8 × 10^−17^
H29BY Sourness	H28BY Sourness	12	1 + 6 + 0	0.917	0.14	0.594	0.450	0.31	5.6 × 10^−15^	1 + 0 + 0	0.525	0.33	0.467	0.348	0.36	1.0 × 10^−16^
H29BY Body	H28BY Body	21	1 + 1 + 0	0.758	0.22	0.514	0.578	0.42	2.8 × 10^−17^	1 + 0 + 0	0.600	0.28	0.573	0.597	0.45	2.5 × 10^−22^
H29BY Bitterness	H28BY Bitterness	29	1 + 0 + 0	0.535	0.24	0.421	0.317	0.34	1.3 × 10^−14^	1 + 0 + 0	0.471	0.25	0.438	0.431	0.36	2.3 × 10^−15^
H29BY Aftertaste	H28BY Aftertaste	19	1 + 0 + 0	0.640	0.26	0.522	0.429	0.41	1.7 × 10^−19^	1 + 0 + 0	0.611	0.27	0.599	0.454	0.40	6.1 × 10^−24^
H29BY Overall quality	H28BY Overall quality	18	1 + 3 + 0	0.859	0.22	0.648	0.496	0.58	5.9 × 10^−22^	1 + 1 + 0	0.703	0.32	0.659	0.650	0.50	8.7 × 10^−26^

* *Ginjo-ka* is a complex aroma composed of various components including ethyl hexanoate, isoamyl acetate, and other esters and alcohols.

## Data Availability

The original contributions presented in this study are included in the article/supplementary material.

## Supplementary Materials

The following supporting information can be downloaded at: https://www.mdpi.com/article/10.3390/metabo15080559/s1, Figure S1: Principal component analysis of the metabolome, general properties, and aroma components in H28BY and H29BY; Table S1: Preparation method of the training samples for the sensory evaluation in H28BY; Table S2: Preparation method of the training samples for the sensory evaluation in H29BY; Table S3: Data of the general properties and aroma components; Table S4: Sensory evaluation data; Table S5: Metabolome data; Table S6: Comparison of the explanatory variable set used for the construction of the prediction model; Table S7: VIPpred of the components for each prediction model; Table S8: Rank of VIPpred of the components for each prediction model; Table S9: VIPpred and correlation coefficients of the components for each prediction model; and Table S10: The top VIPpred variables for each prediction.

## Abbreviations

The following abbreviations are used in this manuscript:

CE	Capillary electrophoresis
TOF-MS	Time-of-flight mass spectrometry
GC	Gas chromatography
QDA	Quantitative descriptive analysis
OPLS	Orthogonal projections to latent structures
UPLC-Q	Ultrahigh-performance liquid chromatography quadrupole
BY	Brewing year
RT	Retention time
PCA	Principal component analysis
VIPpred	Variable importance for prediction predictive
