# Supplementary material for: Modeling the Sensory Characteristics of Japanese Sake Using the Sake Metabolome Analysis Method
Source: Metabolites. 2025 Aug 20;15(8):559. doi: 10.3390/metabo15080559 (PMC12388305; doi:10.3390/metabo15080559)
Supplement: Supplementary file 1 [file metabolites-15-00559-s001.zip › metabolites-3727420-supplementary/metabolites-3727420_SupplementaryInformation.pdf]

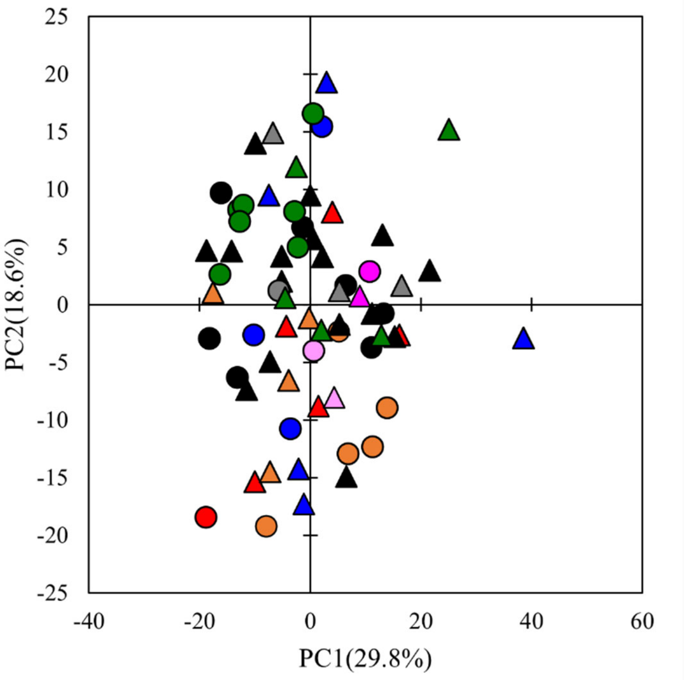

Figure S1. Principal component analysis of the metabolome, general properties, and aroma components in H28BY and H29BY. A score scatter plot of PC1 against PC2 for the integrated data of general properties, aroma components, and metabolome data in H28BY and H29BY. Circles indicate H28BY samples. Triangles indicate H29BY samples. Data for different sake samples are shown, and different colors indicate different rice cultivars.

Table S1. Preparation method of training samples for the sensory evaluation in H28BY

Evaluation attributes of sensory test	Sample for score 4	Sample for score 2	Sample for score 0 or Low
H28BY			
Ginjo-ka	Y-6	The mixture of the score 4 sample and the control sake in a 1:1 ratio	Control sake
Hine-ka	The mixture of a commercial aged sake and the control sake in a 1:1 ratio	-	Control sake
Nama hine-ka	Addition of isovaleraldehyde at a concentration of 790 μg/L to the control sake	The mixture of the score 4 sample and the control sake in a 1:1 ratio	Control sake
Fatty acid smell	Addition of hexanoic acid at a concentration of 13.8 mg/L to the control sake	The mixture of the score 4 sample and the control sake in a 1:1 ratio	Control sake
Grassy/aldehydic	Addition of acetaldehyde at a concentration of 66 mg/L to the control sake	The mixture of the score 4 sample and the control sake in a 1:1 ratio	Control sake
Diacetyl	Addition of diacetyl at a concentration of 498 μg/L to the control sake	The mixture of the score 4 sample and the control sake in a 1:1 ratio	Control sake
Sweetness	Add glucose to the control sake and adjust the glucose concentration to 4%	Add glucose to the control sake and adjust the glucose concentration to 2%	-
Sourness	Add citric acid to the control sake and adjust the acidity to 3.0	Add citric acid to the control sake and adjust the acidity to 1.5	-
Body	-	-	-
Bitterness	Addition of ferulic acid at a concentration of 750 μg/L to the control sake	Addition of ferulic acid at a concentration of 375 μg/L to the control sake	-
Harsh taste/acrid taste	-	-	-
Aftertaste	-	-	-
Overall quality	-	-	-

Table S2. Preparation method of training samples for the sensory evaluation in H29BY

Evaluation attributes of sensory test	Score 4	Score 2	Score 0 (or Low)
H29BY			
Color	-	-	-
Ethyl hexanoate	Add ethyl hexanoate to the control sake and adjust the ethyl hexanoate concentration to 14.5 mg/L	Add ethyl hexanoate to the control sake and adjust the ethyl hexanoate concentration to 7.25 mg/L	Control sake
Isoamyl acetate	Add isoamyl acetate to the control sake and adjust the isoamyl acetate concentration to 4.3 mg/L	Add isoamyl acetate to the control sake and adjust the isoamyl acetate concentration to 2.15 mg/L	Control sake
Nama hine-ka	Addition of isovaleraldehyde at a concentration of 790 μg/L to the control sake	Addition of isovaleraldehyde at a concentration of 395 μg/L to the control sake	Control sake
Ethyl acetate	Add ethyl acetate to the control sake and adjust the ethyl acetate concentration to 90.4 mg/L	Add ethyl acetate to the control sake and adjust the ethyl acetate concentration to 45.2 mg/L	Control sake
Grainy/Sweet aroma	-	-	-
Sweetness	Add glucose to the control sake and adjust the glucose concentration to 4.9%	-	-
Sourness	Add citric acid to the control sake and adjust the acidity to 2.5	Add citric acid to the control sake and adjust the acidity to 1.5	-
Body	-	-	-
Bitterness	Addition of ferulic acid at a concentration of 375 μg/L to the control sake	Addition of ferulic acid at a concentration of 187.5 μg/L to the control sake	-
Astringency	-	-	-
Aftertaste	-	-	-
Overall quality	-	-	-

Table S3. Data of general properties and aroma components

		General properties				Aroma components(ppm)					
		G-1	G-2	G-3	G-4	F-1	F-2	F-3	F-4	F-5	F-6
No.	Sample ID	Ethanol concentration (alcohol content)(%)	Acidity	Amino acid content	Sake meter value (nihonsyu-do)	Ethyl acetate (EtOAc)	n-propanol (nPrOH)	Isobutanol (iBuOH)	Isoamyl acetate (iAmOAc)	Isoamyl alcohol (iAmOH)	Ethyl hexanoate (EtOCap)
1	Y-1	17.3	1.8	1.5	2.9	60.4	100.8	45.5	2.8	140.2	2.9
2	Y-2	16.0	1.4	1.6	1.7	29.9	56.7	41.5	0.8	114.1	5.5
3	Y-3	16.6	1.7	1.2	1.8	41.4	104.3	28.0	1.6	110.6	6.7
4	Y-4	17.7	1.6	1.7	0.9	81.5	110.0	49.4	3.6	147.2	2.5
5	T-1	17.0	1.3	1.0	0.7	69.1	46.8	43.8	2.1	124.9	6.1
6	T-2	18.1	1.2	0.9	3.5	59.7	95.3	27.1	2.1	90.1	6.3
7	T-3	17.4	1.5	1.4	0.0	46.3	66.0	29.5	1.5	103.0	6.4
8	T-4	16.7	1.8	1.1	-4.7	72.0	92.6	39.8	2.5	115.8	2.5
9	T-5	16.9	1.8	1.1	4.2	67.4	91.7	36.4	2.8	110.5	3.2
10	T-6	17.2	1.3	0.9	4.3	49.8	84.0	24.8	2.4	90.8	4.2
11	IS-1	17.4	1.3	1.3	4.0	51.1	77.3	43.8	1.8	126.0	6.9
12	IS-2	16.6	1.3	0.8	2.1	70.8	63.2	47.6	2.5	128.5	0.8
13	IS-3	17.7	1.8	1.2	3.3	54.7	86.4	65.5	2.3	154.3	1.0
14	IS-4	17.8	1.8	1.4	1.6	48.1	82.7	65.9	2.1	154.9	1.0
15	IS-5	17.2	1.8	1.6	1.4	36.3	86.1	56.9	1.4	143.5	0.9
16	IWI-1	17.8	1.5	1.5	3.0	85.1	100.7	48.2	3.1	142.5	2.5
17	KK-1	18.5	1.7	1.9	1.1	82.7	135.7	51.7	3.2	155.4	1.9
18	HN-1	16.3	1.2	1.2	7.2	81.3	60.2	49.8	2.2	135.6	1.6
19	HN-2	16.8	1.5	1.5	1.3	61.4	101.4	28.8	2.4	112.5	6.2
20	Y-5	14.9	1.4	1.0	2.5	23.8	41.0	40.8	1.4	120.5	6.5
21	HN-3	14.8	1.4	1.1	3.8	25.3	40.1	37.8	1.5	114.7	6.3
22	HS-1	15.2	1.2	0.9	8.7	34.1	34.4	54.8	2.1	137.1	4.2
23	G-1	16.1	1.5	1.3	0.0	22.5	45.2	39.0	1.4	119.0	5.4
24	Y-6	17.3	1.2	0.7	3.6	23.2	43.6	33.8	0.8	102.2	9.0
25	Y-7	16.5	1.2	0.9	-2.0	19.9	44.2	27.3	0.9	84.9	10.4
26	T-7	14.5	1.3	1.3	1.0	21.4	31.8	35.7	1.1	123.2	8.1
27	HN-a	17.6	1.9	1.2	9.2	87.2	86.4	75.9	2.5	205.7	1.0
28	Y-a	18.2	1.8	1.3	8.3	87.4	105.9	67.6	2.9	175.3	1.3
29	IS-a	17.3	1.4	1.1	0.0	41.6	75.8	41.2	1.3	122.6	3.9
30	Y-b	16.0	1.2	0.9	3.3	27.2	67.0	29.2	1.4	105.5	5.0
31	IS-b	17.3	1.3	1.3	4.4	44.0	73.3	40.8	1.8	125.6	9.0
32	Y-c	17.5	1.7	1.5	2.1	59.2	89.3	43.3	2.9	145.4	6.0
33	IS-c	15.4	1.5	0.9	2.1	58.6	61.5	36.1	1.6	106.7	1.0
34	Y-d	15.1	1.2	1.3	0.2	24.2	47.5	31.9	0.8	101.4	2.7
35	HN-b	16.6	1.2	1.2	6.7	85.2	62.6	62.4	2.8	160.9	2.3
36	Y-e	16.6	1.2	1.6	3.1	49.2	61.7	48.1	1.1	132.6	5.1
37	IWI-a	17.7	1.6	1.5	1.2	90.4	94.0	54.4	3.3	163.2	2.1
38	KK-a	18.1	1.7	1.7	1.3	81.1	146.3	49.3	2.6	158.5	1.8
39	Y-f	17.8	1.8	1.9	2.1	74.9	106.0	49.0	2.2	145.3	2.5
40	HS-a	17.9	2.3	1.8	2.7	77.1	130.3	62.1	2.0	149.4	0.7
41	R-a	18.3	2.2	2.0	6.8	88.2	116.1	64.3	4.3	142.6	0.7
42	HN-c	16.4	1.7	1.6	1.6	60.9	88.5	26.4	2.6	113.2	6.6
43	Y-g	16.6	1.8	1.4	1.0	49.9	103.2	29.6	2.3	113.8	6.2
44	HS-b	17.0	1.3	1.6	4.1	47.0	74.3	74.0	1.7	167.1	1.9
45	Y-h	16.9	1.4	1.9	4.4	39.8	79.0	56.5	1.5	150.2	1.8
46	HN-d	17.2	1.6	3.5	3.1	77.2	102.4	50.7	2.0	157.0	1.0
47	Y-i	17.0	1.7	1.8	7.3	58.1	90.2	46.5	1.9	144.1	1.0
48	HS-c	15.9	1.4	1.3	0.0	50.8	50.7	52.6	1.7	132.7	5.4
49	Y-j	16.0	1.4	1.3	1.9	33.4	51.5	42.7	1.3	125.7	8.3
50	T-a	16.9	2.5	2.6	-2.2	36.8	115.9	45.8	1.1	152.4	1.1
51	O-a	16.5	1.2	1.3	0.0	46.3	81.3	37.1	0.7	117.1	5.7
52	T-b	16.6	1.5	1.1	-4.8	81.5	89.1	44.2	3.1	124.3	2.6
53	Y-k	16.9	1.7	1.0	-0.4	59.8	80.4	39.5	1.7	123.5	4.8
54	T-c	17.4	1.6	1.4	4.5	69.0	89.6	61.5	3.6	162.5	1.7
55	Y-l	17.5	1.2	0.9	3.3	36.5	78.6	25.2	1.8	97.0	6.0
56	T-d	18.2	1.6	1.7	4.1	62.7	98.9	54.0	2.4	156.7	3.2
57	Y-m	16.9	1.1	1.0	4.5	34.1	50.9	37.4	1.3	104.6	5.4
58	Y-n	16.3	1.4	1.0	0.3	14.0	55.0	36.3	0.2	109.9	6.2
59	Y-o	17.5	1.5	1.5	-5.8	17.1	68.4	46.0	0.3	127.0	6.0
60	HS-d	17.2	1.5	1.7	-2.1	18.7	49.6	46.5	0.4	127.5	7.3
61	HS-e	15.8	1.4	1.1	0.6	30.3	55.3	99.8	1.2	222.6	0.4
62	IS-d	15.9	1.3	0.9	-1.9	21.8	41.4	32.3	0.5	101.2	9.0
63	Y-p	17.0	1.4	1.1	-4.5	34.6	51.2	33.4	1.0	102.7	10.2
64	G-a	17.4	1.5	1.2	-6.1	35.4	54.1	37.6	1.3	114.5	11.2
65	T-e	15.6	1.5	1.4	-7.4	39.9	44.9	42.0	1.4	134.2	14.5
66	HN-e	17.2	1.5	1.2	2.6	40.5	38.7	39.3	1.4	124.5	11.3

Table S4. Sensory evaluation data

No.	Sample ID	Color	*Ginjo-ka*	Ethyl hexanoate	Isoamyl acetate	*Hine-ka*	*Nama hine-ka*	Ethyl acetate	Fatty acid smell	Grassy/aldehydic	Diacetyl	Grainy/Sweet aroma	Sweetness	Sourness	Body	Bitterness	Astringency	Harsh taste/acrid taste	Aftertaste	Overall quality
1	Y-1	-	1.49	-	-	0.89	2.74	-	0.98	1.19	0.91	-	2.50	2.59	3.36	1.90	-	2.37	3.03	3.63
2	Y-2	-	2.20	-	-	0.28	0.49	-	1.21	0.43	0.59	-	2.65	1.40	2.54	1.21	-	0.89	2.06	2.70
3	Y-3	-	2.82	-	-	0.00	0.47	-	0.89	0.27	0.30	-	1.92	1.90	2.07	1.59	-	1.27	1.66	2.32
4	Y-4	-	1.72	-	-	0.41	0.54	-	0.58	1.03	1.02	-	2.66	2.20	2.82	1.76	-	1.75	2.50	3.15
5	T-1	-	3.15	-	-	0.00	0.34	-	0.88	0.50	0.42	-	2.44	1.47	2.17	1.58	-	1.18	1.63	2.17
6	T-2	-	2.60	-	-	0.25	0.20	-	0.91	0.99	0.25	-	2.50	1.51	2.43	1.25	-	1.32	2.26	2.48
7	T-3	-	3.08	-	-	0.02	0.34	-	0.87	0.72	0.23	-	3.35	1.75	2.65	1.06	-	1.08	2.16	2.25
8	T-4	-	2.01	-	-	0.19	0.53	-	0.52	1.00	0.49	-	2.56	2.16	2.57	1.13	-	1.13	2.00	2.70
9	T-5	-	2.01	-	-	0.02	0.65	-	1.07	1.11	0.50	-	1.68	2.39	2.21	1.42	-	1.76	2.49	3.15
10	T-6	-	2.72	-	-	0.19	0.46	-	0.62	0.50	0.39	-	2.99	1.63	2.23	1.30	-	1.12	1.82	2.26
11	IS-1	-	2.18	-	-	0.36	1.77	-	1.08	1.28	0.41	-	3.29	1.75	3.04	1.53	-	1.67	2.52	3.12
12	IS-2	-	1.49	-	-	0.49	1.12	-	0.67	2.18	0.75	-	2.15	1.71	2.30	1.51	-	1.74	2.70	3.73
13	IS-3	-	1.29	-	-	1.30	2.78	-	1.03	1.52	0.77	-	2.42	2.54	3.60	1.82	-	2.14	2.89	4.11
14	IS-4	-	1.25	-	-	1.64	2.66	-	0.79	1.47	0.56	-	2.35	2.44	3.25	2.19	-	2.18	2.95	3.90
15	IS-5	-	1.33	-	-	1.32	2.75	-	1.11	1.42	1.07	-	2.49	2.58	3.44	1.84	-	1.96	3.31	4.14
16	IWI-1	-	1.44	-	-	0.79	0.42	-	0.51	1.09	1.17	-	2.17	2.27	2.45	1.31	-	1.34	2.59	3.26
17	KK-1	-	1.12	-	-	0.73	0.84	-	0.87	0.64	0.66	-	2.10	2.18	3.12	1.64	-	1.56	2.62	3.02
18	HN-1	-	2.17	-	-	0.01	0.56	-	0.46	1.03	0.57	-	1.88	1.79	2.13	1.64	-	1.66	1.92	2.79
19	HN-2	-	1.85	-	-	0.00	0.49	-	0.60	1.50	0.36	-	2.36	2.17	2.35	1.47	-	1.65	2.44	3.01
20	Y-5	-	2.40	-	-	0.14	0.64	-	0.67	0.32	0.39	-	1.29	2.57	1.73	1.68	-	1.38	1.65	3.00
21	HN-3	-	2.05	-	-	0.00	0.33	-	0.89	0.29	0.12	-	1.55	2.60	1.89	1.66	-	1.70	1.43	3.08
22	HS-1	-	1.18	-	-	0.32	0.62	-	0.60	1.21	0.46	-	1.24	2.53	1.83	1.49	-	2.01	2.11	3.50
23	G-1	-	2.23	-	-	0.44	0.63	-	0.61	0.97	0.49	-	1.44	2.34	1.74	1.67	-	1.64	1.63	3.09
24	Y-6	-	2.56	-	-	0.18	0.55	-	1.06	0.53	0.20	-	1.59	1.99	1.53	1.68	-	1.61	2.14	2.57
25	Y-7	-	3.59	-	-	0.01	0.19	-	0.40	0.52	0.26	-	3.37	1.54	2.40	1.32	-	0.68	1.68	1.71
26	T-7	-	2.91	-	-	0.00	0.31	-	1.16	0.74	0.41	-	2.15	1.25	1.24	1.01	-	0.69	1.17	2.42
27	HN-a	1.91	-	1.24	1.94	-	1.90	2.04	-	-	-	1.53	1.64	3.20	2.29	1.86	2.01	-	2.09	3.49
28	Y-a	1.85	-	1.33	1.83	-	2.13	2.24	-	-	-	1.44	2.01	2.99	2.84	2.74	2.63	-	2.85	3.26
29	IS-a	1.95	-	2.61	1.75	-	0.80	1.12	-	-	-	0.73	2.69	1.78	2.19	1.77	1.59	-	1.90	2.00
30	Y-b	2.07	-	1.83	2.27	-	0.57	2.23	-	-	-	0.94	1.70	2.91	2.28	1.95	2.10	-	2.23	2.52
31	IS-b	2.71	-	2.43	1.78	-	1.05	1.36	-	-	-	0.98	2.78	2.10	2.71	1.94	1.48	-	2.48	2.55
32	Y-c	2.19	-	1.94	1.77	-	0.73	1.34	-	-	-	0.95	2.28	2.46	2.62	1.95	2.07	-	2.32	2.56
33	IS-c	1.78	-	1.39	1.83	-	1.29	1.67	-	-	-	0.87	2.03	2.31	1.94	1.63	1.85	-	1.91	2.90
34	Y-d	2.09	-	1.42	1.47	-	2.23	1.49	-	-	-	1.27	2.86	1.82	2.40	1.67	1.60	-	2.19	3.03
35	HN-b	1.80	-	1.52	2.26	-	1.18	1.89	-	-	-	0.81	1.95	2.02	2.13	2.09	1.96	-	2.01	2.89
36	Y-e	3.42	-	1.26	1.38	-	1.95	1.45	-	-	-	2.00	2.67	1.86	2.55	2.18	2.06	-	2.52	3.29
37	IWI-a	1.81	-	1.79	2.31	-	0.50	2.16	-	-	-	0.70	2.08	2.53	2.57	2.08	2.12	-	2.48	2.78
38	KK-a	2.31	-	1.25	1.86	-	1.24	1.91	-	-	-	1.24	2.13	2.35	2.59	2.24	2.10	-	2.33	2.77
39	Y-f	2.64	-	0.95	1.39	-	1.92	2.05	-	-	-	1.66	1.90	2.48	2.53	2.37	2.30	-	2.95	3.41
40	HS-a	1.50	-	1.18	1.88	-	1.13	1.90	-	-	-	1.04	1.85	3.41	2.58	1.97	2.36	-	2.58	3.26
41	R-a	1.80	-	1.69	2.35	-	0.69	1.77	-	-	-	0.67	1.85	2.61	2.11	1.88	1.81	-	1.86	2.68
42	HN-c	2.09	-	1.88	1.52	-	0.82	1.55	-	-	-	1.12	2.58	2.13	2.20	1.49	1.44	-	2.20	2.57
43	Y-g	1.59	-	2.53	1.83	-	0.59	1.06	-	-	-	0.55	2.61	1.98	2.08	1.78	1.74	-	2.21	2.17
44	HS-b	2.62	-	1.41	1.62	-	1.11	1.52	-	-	-	1.26	1.93	2.18	2.17	2.31	2.02	-	2.44	3.18
45	Y-h	2.34	-	1.70	1.73	-	0.88	1.66	-	-	-	1.04	1.90	2.04	2.22	2.13	1.83	-	2.23	2.57
46	HN-d	4.55	-	0.80	1.18	-	2.61	1.73	-	-	-	2.95	2.42	2.61	3.96	2.58	2.41	-	3.61	3.81
47	Y-i	2.72	-	1.33	1.52	-	1.87	1.71	-	-	-	1.34	2.03	2.31	2.63	2.16	2.03	-	2.59	3.23
48	HS-c	2.02	-	1.56	1.69	-	0.91	1.30	-	-	-	1.33	2.11	1.78	2.09	1.68	1.72	-	1.97	3.07
49	Y-j	2.05	-	2.94	1.44	-	0.89	1.02	-	-	-	1.03	2.41	2.07	2.16	1.84	1.57	-	1.92	2.51
50	T-a	3.88	-	1.03	1.13	-	2.06	1.17	-	-	-	2.31	2.63	3.70	3.62	1.74	2.23	-	2.97	3.68
51	O-a	3.12	-	1.14	1.25	-	1.83	1.36	-	-	-	2.39	3.23	1.69	3.01	1.06	1.99	-	2.98	4.28
52	T-b	2.51	-	1.98	2.35	-	1.24	1.32	-	-	-	1.21	3.22	2.27	2.73	1.65	1.46	-	2.72	2.48
53	Y-k	1.78	-	2.22	1.51	-	0.39	0.97	-	-	-	0.29	2.83	2.53	2.23	1.59	1.51	-	2.06	2.34
54	T-c	1.94	-	1.46	2.19	-	0.38	1.94	-	-	-	0.90	1.78	1.97	2.29	2.19	2.04	-	2.24	2.52
55	Y-l	1.25	-	2.72	1.90	-	0.49	1.10	-	-	-	0.50	3.07	1.77	2.18	1.59	1.61	-	1.98	1.96
56	T-d	2.32	-	1.78	1.80	-	1.13	1.48	-	-	-	0.97	2.54	2.44	2.61	2.04	2.13	-	2.63	2.64
57	Y-m	1.88	-	1.98	1.54	-	0.93	0.98	-	-	-	0.93	2.47	1.75	2.16	1.65	1.53	-	1.97	2.39
58	Y-n	1.48	-	2.51	1.51	-	0.25	0.95	-	-	-	0.43	2.34	2.05	1.79	1.67	1.66	-	1.69	2.31
59	Y-o	1.83	-	3.13	1.63	-	0.46	0.97	-	-	-	0.46	2.67	1.88	1.92	1.46	1.44	-	1.67	1.99
60	HS-d	2.07	-	2.93	1.73	-	0.30	1.21	-	-	-	0.70	2.37	2.05	2.14	2.03	1.82	-	2.20	2.13
61	HS-e	2.39	-	1.47	1.59	-	0.85	1.34	-	-	-	1.07	1.90	2.16	1.73	1.70	1.71	-	1.85	2.92
62	IS-d	1.32	-	3.58	1.86	-	0.47	0.95	-	-	-	0.50	2.97	1.91	2.04	1.62	1.47	-	1.61	1.77
63	Y-p	1.21	-	3.64	1.84	-	0.26	0.97	-	-	-	0.15	3.65	1.66	2.53	1.30	1.29	-	2.24	1.90
64	G-a	2.10	-	3.52	1.81	-	0.39	1.03	-	-	-	0.60	3.59	1.89	2.45	1.49	1.56	-	2.17	2.02
65	T-e	1.31	-	3.42	1.67	-	0.43	0.97	-	-	-	0.45	2.88	1.85	1.81	1.28	1.26	-	1.68	1.84
66	HN-e	1.56	-	3.15	1.54	-	0.33	0.75	-	-	-	0.60	2.50	2.30	2.32	2.10	1.92	-	1.78	1.91

Table S5. Metabolome data

No.	Sample ID	Peak-ID	Peak-2382	Peak-3587	Peak-1084	Peak-407	Peak-5919	Peak-4142	Peak-6853	Peak-4436	Peak-3021	Peak-1693	Peak-881	Peak-3537	Peak-6948	Peak-888
		Retention Time	1.2872	1.5553	1.558	1.3355	1.6713	7.6163	7.8903	1.559	1.3183	1.2152	1.2597	1.3375	1.7198	1.7158
		m/z	102.0557	447.1703	285.1174	69.0339	244.1276	325.1095	149.061	123.0657	109.0292	88.0402	74.0603	117.0555	255.1081	75.0444
		Candidate compound	f/L-Glutami			tol_f/D-Ara			nylalanine_f		Glucose_f_3	L-Aspartic a	hreonine_f_	f/Ribitol_f/X		
No.	Sample ID	Replicate														
1	Y-1	1	1547.4974	914.584	1287.2798	1575.7665	2331.167	3518.2817	851.2971	1027.4669	1360.8977	1099.2903	916.0874	1034.3589	20336.592	704.967
2	Y-2	1	2000.8152	767.8319	1089.2488	1718.1484	2716.4082	3047.5537	920.789	887.7679	1323.8108	1939.049	1146.9175	904.0319	16913.455	652.3877
3	Y-3	1	1164.8179	801.9333	889.0714	1704.3579	2186.6414	2269.9521	648.9229	648.6508	1368.4998	962.4644	597.2244	1019.83	25159.639	770.7982
4	Y-4	1	1750.2112	1106.9106	1312.8413	1606.0146	3039.2898	3577.3794	807.021	1013.5336	990.6973	923.1079	769.133	1201.0776	27238.734	817.4789
5	T-1	1	1163.3591	649.9783	679.098	1362.7327	1491.283	2041.7076	609.4467	490.2433	1183.3438	783.8572	483.0904	659.3981	23969.217	734.3079
6	T-2	1	854.8464	490.8874	494.6992	1397.6301	1751.4816	1383.657	530.4461	369.0785	1433.0697	698.8298	411.8257	671.4382	19553.566	625.3306
7	T-3	1	1495.0537	558.9619	619.8107	1612.9381	2293.3232	1019.9279	867.8364	446.9395	1361.6731	1111.4491	733.8426	884.0448	15668.63	568.7764
8	T-4	1	989.9849	775.574	835.769	1619.6667	1979.2932	1592.0449	671.4097	634.1136	1659.761	836.9684	541.7152	910.0902	23078.615	702.3229
9	T-5	1	1141.6624	397.4846	401.7283	1411.4369	1455.4802	1438.1614	631.7585	291.0475	1261.8873	761.1782	563.0623	972.5042	19745.205	638.3734
10	T-6	1	914.2872	491.817	505.7527	1509.144	1567.5078	1229.4419	550.8818	371.9175	1577.2112	805.7888	461.6122	836.4305	15464.921	548.1719
11	IS-1	1	1569.1147	916.6261	1148.485	1601.948	1714.9796	3262.4556	759.2585	899.3287	1311.3007	1122.7549	753.4878	943.2052	23446.551	730.9281
12	IS-2	1	887.165	1358.6287	1893.8838	1776.832	1210.3398	3752.873	377.8197	1492.061	1450.5178	836.0779	404.6906	1185.5558	22591.852	728.8145
13	IS-3	1	1342.1978	626.8394	874.4415	1540.2733	2049.3694	3780.4902	579.1024	719.1068	1421.885	1014.5204	757.4523	1208.8457	19676.363	668.0219
14	IS-4	1	1529.9397	560.5926	789.1591	1547.9725	1822.6968	3898.762	671.5401	624.9681	1363.6754	1010.8263	836.0356	1167.585	20685.529	666.0005
15	IS-5	1	1644.1417	568.2502	826.6771	1512.6855	2140.188	3540.9395	731.4845	647.0898	1329.5624	1122.9563	920.1285	1119.201	19727.102	676.5138
16	IWI-1	1	1773.8739	1181.5072	1436.1644	1453.8462	2532.8667	3874.813	692.9365	1076.5881	829.1713	838.9814	706.0074	1128.5133	26577.535	806.2174
17	KK-1	1	2200.8838	1042.1211	1284.9985	1379.387	3311.4199	3979.7588	881.9892	978.8708	808.1347	1028.3171	914.8399	957.5256	27077.699	832.2366
18	HN-1	1	1521.026	944.0207	1339.9897	1385.6534	2209.8728	4550.2041	531.7702	1073.0341	1126.6057	889.3503	648.5686	911.2887	23941.557	753.7265
19	HN-2	1	1457.7627	828.7358	907.2553	1616.1327	2435.6274	1563.557	890.619	669.5613	1205.1478	1008.287	704.4504	999.6801	23107.441	705.3737
20	Y-5	1	1443.0145	915.2183	1083.4288	1199.2805	1668.8242	3595.1646	558.9337	828.6611	644.426	812.2692	587.3494	1022.2076	27212.356	807.9136
21	HN-3	1	1518.6754	930.9327	1069.4294	1278.5863	1722.0916	3225.4148	626.9005	783.4492	738.5016	888.3547	653.861	1049.7039	28365.078	826.2184
22	HS-1	1	970.1495	1038.2272	1246.5848	1329.9178	1006.0555	4476.6484	391.7856	966.0915	707.2696	530.1301	319.9596	1099.4783	27940.092	834.5724
23	G-1	1	1607.6125	923.4835	1088.9335	1246.0359	1738.1089	3318.3408	699.0649	804.6415	730.3845	939.4028	699.9352	993.0955	28119.457	819.5558
24	Y-6	1	1061.5593	765.1557	850.0024	1308.6941	1831.3215	3572.1909	561.665	624.4902	909.1398	668.8219	397.7729	714.9176	25004.502	753.4991
25	Y-7	1	877.0787	211.9122	206.4903	1620.4534	1258.8966	1418.204	603.9463	135.6112	1547.3679	761.8142	417.8032	484.7218	13214.561	486.1981
26	T-7	1	1561.0985	751.8554	852.4915	1687.939	2164.2905	1893.2837	862.7424	616.2609	1230.7505	979.6746	590.2557	1250.4119	23776.736	733.3278
1	Y-1	2	1565.486	911.585	1323.0416	1498.1223	2223.7043	3394.6433	840.981	1020.0852	1293.6896	1080.6987	932.7861	988.2734	20361.443	688.3712
2	Y-2	2	1967.1737	766.6649	1122.0695	1677.1807	2579.2153	2993.355	903.6746	884.2485	1268.7245	1861.382	1148.38	872.9178	17256.354	622.7462
3	Y-3	2	1146.0211	782.0296	905.9543	1669.3911	2093.5759	2235.7668	629.0458	651.2469	1292.1429	908.3022	598.2719	977.7183	25132.44	736.0156
[illegible]	[illegible]	[illegible]	[illegible]	[illegible]	[illegible]	[illegible]	[illegible]	[illegible]	[illegible]	[illegible]	[illegible]	[illegible]	[illegible]	[illegible]	[illegible]	[illegible]

Table S6. Comparison of explanatory variable set used for prediction model construction

Evaluation attributes of sensory test	R^2Y			Q^2			RMSEE			CV-ANOVA *p*-value		
	Metabolome	Metabolome + General analysis	General analysis	Metabolome	Metabolome + General analysis	General analysis	Metabolome	Metabolome + General analysis	General analysis	Metabolome	Metabolome + General analysis	General analysis
**H28BY**												
Ginjo-ka	0.876	0.881	0.783	0.672	0.685	0.692	0.243	0.238	0.317	4.9E-14	1.3E-14	5.8E-18
Hine-ka	0.965	0.958	0.925	0.800	0.810	0.820	0.088	0.097	0.129	1.0E-19	2.0E-20	3.5E-21
Nama hine-ka	0.990	0.991	0.763	0.834	0.842	0.609	0.086	0.082	0.422	9.5E-21	1.7E-21	1.6E-11
Grassy/aldehydic	0.697	0.700	0.323	0.252	0.264	0.206	0.259	0.258	0.382	1.7E-03	1.0E-03	1.8E-04
Diacetyl	0.508	0.521	0.599	0.389	0.404	0.524	0.193	0.190	0.174	9.7E-09	3.7E-09	7.9E-13
Sweetness	0.950	0.952	0.414	0.682	0.697	0.135	0.136	0.134	0.457	3.3E-13	7.0E-14	3.0E-02
Sourness	0.959	0.958	0.705	0.675	0.672	0.400	0.090	0.090	0.238	9.5E-12	1.3E-11	8.0E-05
Body	0.690	0.700	0.762	0.615	0.626	0.583	0.337	0.331	0.299	2.9E-16	9.3E-17	7.6E-12
Bitterness	0.450	0.452	0.494	0.258	0.262	0.238	0.203	0.203	0.196	1.4E-05	1.1E-05	4.7E-04
Harsh taste/acrid taste	0.950	0.954	0.754	0.610	0.646	0.585	0.101	0.097	0.221	2.3E-09	1.3E-10	1.1E-10
Aftertaste	0.545	0.560	0.603	0.437	0.454	0.531	0.362	0.357	0.339	4.5E-10	1.4E-10	4.5E-13
Overall quality	0.917	0.920	0.772	0.759	0.760	0.676	0.180	0.178	0.296	1.6E-18	1.6E-18	3.5E-17
**H29BY**												
Color	0.919	0.921	0.660	0.691	0.670	0.470	0.199	0.196	0.402	1.7E-23	5.2E-22	1.0E-13
Ethyl hexanoate	0.834	0.839	0.841	0.712	0.716	0.790	0.331	0.326	0.324	2.4E-28	1.1E-28	4.7E-36
Isoamyl acetate	0.768	0.776	0.604	0.371	0.401	0.463	0.149	0.146	0.194	1.2E-08	9.5E-10	2.2E-13
Nama hine-ka	0.826	0.826	0.613	0.646	0.646	0.434	0.269	0.268	0.400	2.4E-23	2.2E-23	3.5E-12
Ethyl acetate	0.637	0.644	0.652	0.511	0.521	0.547	0.251	0.249	0.246	4.1E-17	1.3E-17	5.5E-19
Grainy/Sweet aroma	0.866	0.865	0.640	0.624	0.622	0.464	0.217	0.217	0.353	2.2E-20	2.7E-20	1.9E-13
Sweetness	0.747	0.751	0.629	0.659	0.665	0.528	0.257	0.255	0.311	5.4E-26	2.0E-26	5.3E-18
Sourness	0.917	0.919	0.720	0.594	0.596	0.542	0.140	0.138	0.253	5.6E-15	4.2E-15	8.0E-16
Body	0.758	0.760	0.584	0.514	0.516	0.239	0.217	0.216	0.286	2.8E-17	2.3E-17	2.1E-05
Bitterness	0.535	0.541	0.622	0.421	0.427	0.416	0.237	0.236	0.215	1.3E-14	6.9E-15	2.0E-11
Astringency	0.585	0.592	0.534	0.508	0.515	0.470	0.208	0.207	0.221	9.7E-19	4.3E-19	7.4E-17
Aftertaste	0.640	0.640	0.616	0.522	0.522	0.372	0.257	0.256	0.267	1.7E-19	1.8E-19	9.7E-10
Overall quality	0.859	0.860	0.649	0.648	0.651	0.511	0.225	0.225	0.354	5.9E-22	3.9E-22	1.2E-15

Table S7. VIPpred of components for each prediction model

VIPpred	G-1 Ethanol concentration (alcohol content)	G-2 Acidity	G-3 Amino acid content	G-4 Sake meter value (nihonsyu-do)	F-1 Ethyl acetate (EtOAc)	F-2 n-propanol (nPrOH)	F-3 Isobutanol (iBuOH)	F-4 Isoamyl acetate (iAmOAc)	F-5 Isoamyl alcohol (iAmOH)	F-6 Ethyl hexanoate (EtOCap)
**H28BY**										
Ginjo-ka	0.65	1.26	0.92	0.51	1.05	1.04	1.88	1.32	1.95	2.00
Hine-ka	1.00	1.23	0.86	0.03	0.29	0.77	1.70	0.46	1.49	1.42
Nama hine-ka	0.76	1.27	0.64	0.14	0.14	0.60	1.53	0.29	1.35	1.23
Grassy/aldehydic	0.79	1.10	0.20	0.20	1.21	0.93	1.48	1.28	1.34	2.01
Diacetyl	0.58	1.01	1.28	0.04	0.52	0.83	1.33	0.50	1.43	1.22
Sweetness	1.34	0.05	0.62	1.15	0.56	0.88	0.53	0.13	0.55	0.20
Sourness	0.20	2.22	0.74	0.70	0.12	0.86	1.78	1.12	1.84	1.67
Body	0.75	1.09	1.27	0.33	0.48	0.96	0.97	0.38	1.04	1.03
Bitterness	0.32	0.81	0.84	0.40	0.31	0.41	1.72	0.38	1.66	1.35
Harsh taste/acrid taste	0.84	1.40	0.44	1.25	0.79	0.90	1.92	1.22	1.88	1.87
Aftertaste	0.69	1.05	1.16	0.13	0.49	0.86	1.23	0.43	1.24	1.21
Overall quality	0.36	1.24	0.61	0.53	0.49	0.55	1.90	0.81	1.79	1.80
**H29BY**										
Color	0.06	0.37	1.70	0.24	0.34	0.79	0.27	0.01	0.59	0.83
Ethyl hexanoate	0.54	0.80	1.02	1.33	1.50	1.42	1.05	1.11	1.20	1.84
Isoamyl acetate	0.74	0.20	1.32	0.70	1.49	0.44	0.70	1.96	0.35	0.60
Nama hine-ka	0.10	0.45	1.26	0.78	0.85	0.88	0.36	0.25	0.62	1.17
Ethyl acetate	0.83	0.70	0.59	1.56	1.63	1.26	1.56	1.32	1.57	1.87
Grainy/Sweet aroma	0.05	0.37	1.56	0.71	0.67	0.87	0.62	0.13	0.91	1.16
Sweetness	0.81	0.51	0.36	1.44	1.10	0.87	1.81	1.00	1.67	1.58
Sourness	1.25	2.48	1.28	1.00	1.50	1.97	0.98	1.15	1.37	1.66
Body	0.32	0.66	1.54	0.22	0.75	0.92	0.09	0.33	0.27	0.61
Bitterness	0.85	0.81	1.48	1.01	1.09	1.17	1.14	0.79	1.27	1.33
Astringency	0.81	0.90	1.48	0.95	1.14	1.24	0.99	0.80	1.15	1.34
Aftertaste	0.64	0.90	1.65	0.53	0.91	1.14	0.42	0.57	0.67	0.93
Overall quality	0.22	0.63	1.21	1.14	1.07	1.14	1.03	0.52	1.20	1.66

Table S8. Rank of VIPpred of components for each prediction model

Rank of VIPpred	G-1 Ethanol concentration (alcohol content)	G-2 Acidity	G-3 Amino acid content	G-4 Sake meter value (nihonsyu-do)	F-1 Ethyl acetate (EtOAc)	F-2 n-propanol (nPrOH)	F-3 Isobutanol (iBuOH)	F-4 Isoamyl acetate (iAmOAc)	F-5 Isoamyl alcohol (iAmOH)	F-6 Ethyl caproate (EtOCap)
**H28BY**										
Ginjo-ka	252	111	195	293	164	168	5	99	4	1
Hine-ka	177	122	213	436	379	236	22	317	74	85
Nama hine-ka	226	117	257	409	410	276	66	366	103	128
Grassy/aldehydic	219	151	395	393	106	196	58	90	81	6
Diacetyl	300	195	131	428	318	241	115	319	77	142
Sweetness	76	428	289	122	308	174	315	405	312	392
Sourness	378	1	217	224	399	188	16	147	8	47
Body	255	175	115	383	344	206	202	368	187	190
Bitter	348	231	222	327	353	320	24	330	31	82
Harsh taste/acrid taste	197	82	308	102	209	183	16	109	22	23
Aftertaste	267	182	159	422	338	219	141	357	138	145
Overall quality	331	115	259	286	296	281	7	212	27	20
**H29BY**										
Color	420	335	34	365	344	218	357	436	271	212
Ethyl hexanoate	290	238	173	101	60	81	167	151	132	10
Isoamyl acetate	248	392	95	259	64	321	260	2	343	285
Nama hine-ka	421	323	117	254	234	210	351	378	287	134
Ethyl acetate	203	251	275	67	49	98	65	88	62	28
Grainy/Sweet aroma	428	335	49	250	256	216	258	403	209	143
Sweetness	180	260	306	85	124	171	34	144	48	71
Sourness	113	1	110	157	51	11	164	124	81	36
Body	356	261	55	385	232	191	416	355	368	269
Bitter	240	250	59	189	167	144	153	255	112	85
Astringency	241	226	51	209	162	126	200	246	156	95
Aftertaste	284	199	12	311	197	148	342	303	275	189
Overall quality	385	276	131	146	165	147	182	300	133	28

Table S9. VIPpred and correlation coefficients of components for each prediction model

	H28BY													H29BY												
Peak-ID	Ginjo-ka	Hine-ka	Nama hine-ka	Fatty acid smell	Grassy/aldehydic	Diacetyl	Sweetness	Sourness	Body	Bitterness	Harsh taste/acrid taste	Aftertaste	Overall quality	Color	Ethyl hexanoate	Isoamyl acetate	Nama hine-ka	Ethyl acetate	Grainy/Sweet aroma	Sweetness	Sourness	Body	Bitterness	Astringency	Aftertaste	Overall quality
Peak-4933	0.10	-0.40	0.16	0.77	0.26	-0.21	0.82	-0.51	0.68	-0.11	0.18	0.49	-0.21	0.90	-0.97	-0.84	0.94	0.53	1.05	-0.14	0.33	0.74	0.49	0.58	0.65	1.07
Peak-966	0.10	-0.08	0.34	0.89	0.35	0.16	1.32	-0.96	0.78	-0.04	-0.04	0.59	-0.16	1.17	-0.77	-1.11	0.88	0.32	1.17	0.03	0.01	0.87	0.59	0.63	0.75	0.96
Peak-480	0.12	-0.72	-0.15	0.35	-0.01	-0.27	0.01	-0.08	0.31	-0.13	0.23	-0.20	-0.27	0.54	-1.09	-0.33	0.92	0.76	0.84	-0.32	0.69	0.56	0.42	0.52	0.50	1.08
Peak-2387	0.05	-0.34	0.19	0.80	0.31	-0.17	0.85	-0.54	0.71	-0.07	0.21	0.53	-0.17	0.90	-0.94	-0.83	0.93	0.50	1.04	-0.11	0.29	0.74	0.47	0.56	0.64	1.04
Peak-4551	0.11	-0.39	0.17	0.79	0.23	-0.22	0.83	-0.51	0.69	-0.11	0.20	0.50	-0.22	0.90	-0.96	-0.85	0.93	0.50	1.05	-0.11	0.32	0.74	0.48	0.57	0.65	1.07
Peak-3028	-0.01	0.49	0.53	0.84	0.30	0.02	1.55	-1.05	0.72	-0.10	-0.08	0.58	-0.02	1.21	-0.06	-1.41	0.43	-0.29	0.92	0.26	-0.57	0.74	0.47	0.43	0.63	0.37
Peak-1040	0.15	-0.37	0.19	0.78	0.23	-0.25	0.94	-0.61	0.68	-0.12	0.19	0.49	-0.25	0.95	-0.98	-0.90	0.95	0.52	1.09	-0.16	0.31	0.74	0.51	0.59	0.65	1.10
Peak-965	0.04	-0.55	-0.02	0.35	0.25	-0.08	0.00	-0.09	0.34	-0.03	0.36	0.26	-0.08	0.43	-1.10	-0.10	0.86	0.83	0.74	-0.38	0.68	0.48	0.38	0.49	0.43	1.03
Peak-6093	1.03	-0.93	-0.80	0.50	-0.80	-0.80	-0.21	-0.76	-0.69	-1.02	-1.16	-0.83	-0.80	-0.10	0.08	-0.65	0.06	0.04	-0.10	0.00	0.47	-0.10	-0.08	0.04	-0.07	-0.04
Peak-1767	0.69	-0.24	-0.18	0.13	-0.35	-0.49	0.79	-1.15	-0.06	-0.61	-0.83	-0.24	-0.49	0.89	0.19	-1.45	0.23	-0.37	0.57	0.26	-0.31	0.48	0.28	0.25	0.40	-0.07
Peak-174	-0.04	-0.52	0.08	0.55	0.16	-0.16	0.31	-0.02	0.49	-0.06	0.37	0.36	-0.16	0.58	-1.05	-0.38	0.88	0.70	0.85	-0.28	0.58	0.55	0.40	0.50	0.49	1.05
Peak-570	-0.06	0.55	0.73	0.65	0.29	0.49	0.66	0.13	0.53	0.57	0.03	0.55	0.49	1.01	-0.77	-0.39	0.88	0.83	0.91	-0.55	-0.01	0.85	0.98	0.95	0.87	0.69
Peak-402	-0.21	0.65	0.83	0.66	0.48	0.66	0.59	0.09	0.57	0.65	0.16	0.60	0.66	0.95	-0.79	-0.27	0.85	0.88	0.85	-0.61	-0.06	0.78	0.95	0.93	0.82	0.69
Peak-3277	-0.13	0.60	0.82	0.70	0.40	0.58	0.65	0.00	0.58	0.62	0.10	0.60	0.58	1.00	-0.79	-0.33	0.88	0.87	0.90	-0.59	0.08	0.84	0.98	0.96	0.86	0.71
Peak-172	-0.13	0.66	0.93	0.72	0.53	0.64	0.69	-0.16	0.61	0.69	0.11	0.65	0.64	1.00	-0.77	-0.36	0.93	0.84	0.92	-0.51	-0.04	0.89	0.95	0.93	0.85	0.69
Peak-5000	-0.17	0.67	0.93	0.84	0.48	0.62	0.78	0.04	0.72	0.67	0.14	0.72	0.62	1.08	-0.83	-0.41	0.95	0.88	0.98	-0.57	0.12	0.94	1.06	1.04	0.96	0.75
Peak-3345	0.34	0.32	0.68	0.87	0.30	-0.17	0.87	-1.16	0.60	-0.29	-0.24	0.57	0.17	0.89	-0.74	-0.20	0.86	0.76	0.85	-0.58	0.95	1.00	1.20	1.18	1.14	0.75
Peak-4869	-0.40	0.64	0.63	1.23	0.04	0.31	0.68	0.66	1.11	0.38	0.30	0.90	0.31	1.22	-0.19	-1.82	0.65	-0.48	0.98	0.64	0.77	1.18	0.60	0.66	1.10	0.46
Peak-3082	-0.69	1.35	1.66	1.61	1.11	1.04	1.34	0.45	1.50	0.99	0.83	1.42	1.04	1.36	-0.91	-0.89	1.23	0.57	1.18	-0.11	0.69	1.54	1.30	1.35	1.55	0.90
Peak-7242	-0.75	1.39	1.70	1.63	1.18	1.10	1.34	0.48	1.54	1.04	0.88	1.47	1.10	1.35	-0.94	-0.85	1.24	0.63	1.18	-0.16	0.71	1.53	1.31	1.36	1.55	0.91
Peak-2401	-1.39	1.70	1.66	1.41	1.55	1.58	0.52	0.99	1.48	1.48	1.14	1.56	1.58	1.66	-1.39	-1.05	1.59	0.98	1.63	-0.48	1.62	1.63	1.49	1.57	1.66	1.53
Peak-1074	1.11	-0.83	-0.41	0.08	-0.74	-0.83	0.55	-0.54	-0.22	-1.05	-0.86	-0.49	-0.83	0.74	0.40	-1.25	0.24	-0.85	0.41	0.96	-0.11	0.98	0.30	0.33	0.82	-0.23
Peak-5155	0.32	-0.28	-0.32	0.59	-0.59	0.58	0.86	-0.23	0.44	-0.47	-0.53	0.16	-0.58	1.29	-0.36	-1.39	0.72	0.22	1.08	0.37	0.83	1.29	0.87	0.90	1.28	0.55
Peak-747	-0.03	-0.41	-0.73	-0.11	-0.61	-0.57	-0.14	0.30	0.06	-0.51	-0.19	0.21	-0.57	-0.32	0.35	-0.21	-0.52	-0.58	-0.34	0.29	0.46	-0.26	-0.23	-0.22	-0.07	-0.25
Peak-2845	-1.26	1.42	1.49	0.68	1.92	1.67	0.14	0.82	0.81	1.23	1.28	1.01	1.67	1.09	-1.23	-0.07	1.21	1.16	1.04	-0.66	0.94	1.08	1.13	1.18	1.10	1.04
Peak-4577	-1.22	1.33	0.77	1.15	0.65	0.90	0.77	0.33	1.24	1.16	0.53	1.26	0.90	1.65	-1.08	-0.90	1.31	0.91	1.58	-0.58	0.72	1.48	1.49	1.43	1.45	1.14
Peak-894	-1.49	1.71	1.53	1.39	1.19	1.60	0.26	1.16	1.44	1.57	1.16	1.52	1.60	1.63	-1.34	-0.95	1.34	1.18	1.59	-0.97	1.15	1.38	1.75	1.71	1.63	1.40
Peak-5848	-1.02	1.46	1.58	1.45	1.12	1.28	0.68	0.59	1.43	1.44	0.83	1.51	1.28	1.89	-1.51	-1.31	1.96	1.06	1.94	-0.36	1.11	1.80	1.42	1.51	1.61	1.72
Peak-2647	-0.02	0.41	0.44	1.40	-0.15	0.08	1.43	-0.79	1.18	-0.30	-0.42	0.95	-0.08	1.47	-0.53	-1.72	1.10	0.04	1.26	0.39	-0.18	1.44	0.96	1.02	1.38	0.77
Peak-169	-0.06	0.23	0.38	1.29	-0.52	0.08	0.85	-0.21	1.02	-0.33	-0.18	0.82	-0.08	1.70	-0.76	-1.74	1.31	0.24	1.61	-0.06	0.60	1.61	1.26	1.28	1.55	1.09
Peak-5728	-1.06	1.48	1.61	1.44	1.19	1.32	0.68	0.66	1.44	1.45	0.89	1.52	1.32	1.88	-1.47	-1.27	1.93	1.03	1.92	-0.33	1.12	1.81	1.42	1.50	1.61	1.67
Peak-2456	-0.17	0.57	0.60	1.02	-0.02	0.19	0.84	0.53	0.89	0.13	0.11	0.66	0.19	1.25	-0.47	-1.24	0.89	0.11	1.05	-0.10	0.38	1.36	1.15	1.15	1.43	0.57
Peak-2822	0.00	0.28	0.48	1.30	-0.43	0.04	0.76	-0.08	1.03	-0.41	-0.08	0.85	0.04	1.67	-0.79	-1.63	1.31	0.29	1.57	-0.02	0.58	1.60	1.29	1.31	1.56	1.08
Peak-5382	-0.75	0.80	0.67	-0.67	0.15	0.60	0.19	1.45	0.71	0.47	0.92	0.61	0.60	1.09	-0.84	-0.55	0.70	0.81	1.00	-0.83	1.12	1.04	1.53	1.44	1.39	0.77
Peak-2213	-1.21	1.67	1.72	1.61	1.14	1.40	0.73	1.09	1.61	1.46	1.11	1.63	1.40	1.72	-1.54	-1.25	1.74	1.06	1.69	-0.51	1.50	1.65	1.53	1.65	1.73	1.67
Peak-5682	-1.50	1.25	1.42	-0.97	1.49	1.46	0.33	2.09	1.18	1.09	1.80	1.18	1.46	-0.30	-0.90	1.36	0.02	1.35	-0.23	-1.38	1.31	-0.16	0.83	0.80	0.40	0.37
Peak-3547	-1.77	1.39	1.45	-0.90	1.93	1.77	-0.04	1.82	1.17	1.45	1.76	1.32	1.77	-0.56	-0.75	1.30	0.04	1.14	-0.42	-1.06	0.73	-0.51	0.21	0.25	-0.15	0.33
Peak-3490	-1.75	1.34	1.43	-0.88	1.95	1.76	-0.02	1.81	1.15	1.42	1.79	1.30	1.76	-0.61	-0.72	1.37	-0.09	1.13	-0.47	-1.06	0.79	-0.56	0.16	0.20	-0.20	0.30
Peak-1693	-0.15	0.45	0.55	1.45	-0.10	0.18	0.87	-0.59	1.21	-0.71	-0.29	1.11	0.18	1.76	-0.91	-1.92	1.57	0.20	1.70	0.31	0.45	1.71	1.09	1.20	1.54	1.30
Peak-814	-0.19	0.49	0.58	1.42	-0.07	0.23	0.82	-0.56	1.19	-0.78	-0.26	1.11	0.23	1.73	-0.95	-1.86	1.60	0.25	1.69	0.28	0.43	1.68	1.08	1.19	1.52	1.33
Peak-2505	-1.54	1.23	1.39	1.25	1.73	1.50	0.57	1.38	1.42	1.30	1.46	1.46	1.50	0.67	-1.47	0.50	0.93	1.51	0.71	-1.10	1.42	0.75	1.21	1.28	1.06	1.13
Peak-3348	-0.20	0.52	0.59	1.43	-0.01	0.26	0.83	-0.62	1.21	-0.80	-0.27	1.14	0.26	1.75	-0.96	-1.92	1.63	0.24	1.72	0.32	0.39	1.69	1.05	1.17	1.50	1.36
Peak-2611	0.58	-0.87	-1.14	-0.49	-0.99	-0.99	0.06	-0.08	-0.51	-1.05	-0.94	-0.73	-0.99	-0.30	0.34	0.15	-0.79	-0.37	-0.54	-0.06	0.77	-0.31	0.08	-0.06	-0.12	-0.49
Peak-5769	-0.15	0.44	0.52	1.35	-0.01	0.21	0.79	-0.73	1.13	-0.75	-0.34	1.07	0.21	1.76	-0.95	-1.86	1.66	0.23	1.74	0.37	0.39	1.73	1.01	1.13	1.47	1.34
Peak-6612	0.42	-0.34	0.18	0.43	-0.50	-0.19	0.25	0.51	0.21	0.31	0.15	-0.01	-0.19	0.83	-0.19	-0.89	0.58	0.10	0.76	-0.09	0.42	1.00	1.01	0.95	1.10	0.38
Peak-5409	0.45	-0.39	-0.35	0.58	-0.78	0.66	0.73	-0.51	0.38	-0.47	-0.64	-0.10	-0.66	0.36	0.27	-1.36	-0.01	-0.74	0.13	0.60	-0.38	0.41	-0.23	-0.28	0.64	-0.06
Peak-7920	-0.97	1.27	1.59	1.41	1.21	1.23	0.80	0.63	1.41	1.28	0.91	1.42	1.23	0.97	-1.23	-1.05	1.28	0.97	1.06	-0.61	1.32	0.86	0.97	1.10	1.04	1.39
Peak-3226	-0.37	0.15	-0.08	0.77	-0.37	0.11	0.48	-0.03	0.74	-0.24	-0.15	0.57	-0.11	1.00	-0.86	-1.12	0.85	0.37	1.01	-0.26	0.51	0.88	1.01	1.07	1.20	1.02
Peak-881	-0.91	1.12	1.05	1.55	0.20	0.83	0.52	0.83	1.44	1.14	0.72	1.37	0.83	1.71	-1.08	-1.39	1.40	0.65	1.67	-0.33	0.91	1.59	1.50	1.50	1.65	1.28
Peak-2522	-0.91	1.17	0.99	1.58	0.52	0.80	0.74	0.31	1.50	1.06	0.40	1.42	0.80	1.44	-0.56	-1.95	1.21	0.19	1.30	0.64	-0.20	1.44	0.75	0.86	1.30	0.89
Peak-4116	-0.87	1.12	1.08	1.60	0.29	0.82	0.65	0.65	1.49	1.14	0.65	1.42	0.82	1.72	-1.01	-1.36	1.40	0.59	1.66	-0.24	0.80	1.63	1.44	1.44	1.60	1.22
Peak-4813	-0.07	0.08	0.19	0.63	-0.56	0.06	0.10	0.65	0.46	0.14	0.22	0.31	0.06	1.09	-0.53	-0.99	0.73	0.49	1.03	-0.58	0.69	1.03	1.39	1.29	1.30	0.72
Peak-8418	-1.30	1.62	1.50	1.34	1.73	1.42	0.89	0.37	1.45	1.32	1.00	1.52	1.42	1.49	-1.39	-0.91	1.51	0.83	1.38	-0.17	0.98	1.29	0.89	1.06	1.17	1.39
Peak-606	-1.15	1.63	1.20	1.31	0.75	1.11	0.74	0.54	1.33	1.33	0.75	1.38	1.11	1.41	-0.49	-1.29	0.89	0.42	1.21	-0.33	0.38	1.23	1.28	1.18	1.25	0.64
Peak-972	-0.73	0.64	0.28	0.83	-0.45	0.49	-0.23	0.72	0.74	0.81	0.37	0.72	0.49	1.34	-0.78	-1.26	0.88	0.74	1.33	-0.90	0.83	1.02	1.57	1.45	1.36	1.04
Peak-2735	-0.13	-0.31	-0.41	-0.70	0.12	0.10	-0.79	0.48	-0.63	-0.15	0.21	-0.55	0.10	-0.19	0.33	0.30	-0.64	0.24	-0.27	-0.81	-0.60	-0.57	0.28	-0.09	-0.22	-0.30
Peak-2382	-0.83	0.78	0.44	1.05	-0.33	0.59	-0.11	0.69	0.95	0.97	0.41	0.93	0.59	1.52	-0.93	-1.39	1.07	0.77	1.51	-0.81	0.88	1.20	1.63	1.54	1.49	1.20
Peak-6727	-0.82	0.65	0.19	0.79	-0.39	0.52	-0.33	0.58	0.73	0.89	0.29	0.73	0.52	1.34	-0.81	-1.28	0.85	0.81	1.35	-1.00	0.85	0.95	1.60	1.46	1.33	1.08
Peak-2443	-0.27	0.58	0.53	1.35	0.67	0.16	1.86	-1.08	1.28	-0.44	-0.55	1.11	0.16	1.48	-0.91	-1.02	1.39	0.54	1.37	-0.03	0.81	1.63	1.25	1.31	1.53	1.06
Peak-1620	-0.24	0.55	1.04	0.99	1.60	0.53	1.62	-0.47	1.03	-0.31	0.20	0.94	0.53	-0.27	0.17	0.38	-0.16	-0.55	-0.52	1.27	-0.36	0.27	-0.89	-0.64	0.24	-0.46
Peak-1994	0.28	0.24	0.68	0.77	1.25	0.00	1.97	-1.40	0.75	-0.12	-0.42	0.62	0.00	-0.10	0.04	0.28	0.48	-0.63	-0.28	1.56	-0.43	0.54	-0.96	-0.64	0.13	-0.21
Peak-7820	0.35	0.18	0.64	0.77	1.17	-0.06	1.99	-1.37	0.73	-0.19	-0.47	0.59	-0.06	-0.07	0.10	0.22	0.45	-0.72	-0.29	1.65	-0.51	0.56	-0.98	-0.66	0.12	-0.25
Peak-4559	0.41	0.08	0.52	0.67	1.11	-0.16	1.98	-1.46	0.65	-0.28	-0.61	0.50	-0.16	-0.10	0.10	0.16	0.45	-0.74	-0.29	1.65	-0.51	0.54	-1.01	-0.68	0.14	-0.24
Peak-4312	-0.06	-0.21	-0.24	0.66	-0.69	0.30	0.19	0.16	0.49	-0.12	-0.27	-0.27	-0.30	0.66	-0.01	-1.39	0.16	-0.47	0.43	-0.33	0.21	0.59	0.52	0.55	0.85	0.18
Peak-2183	0.42	0.11	0.56	0.75	1.10	-0.15	2.01	-1.53	0.70	-0.25	-0.58	0.54	-0.15	-0.15	0.15	0.21	0.40	-0.76	-0.35	1.66	-0.54	0.50	-1.03	-0.71	0.17	-0.30
Peak-3021	0.43	0.11	0.55	0.72	1.08	-0.17	2.01	-1.52	0.68	-0.27	-0.60	0.52	-0.17	-0.15	0.15	0.21	0.41	-0.77	-0.33	1.68	-0.53	0.51	-1.04	-0.72	0.18	-0.28
Peak-6292	0.38	0.17	0.61	0.76	1.12	-0.10	1.98	-1.36	0.72	-0.22	-0.53	0.56	-0.10	-0.06	0.12	0.12	0.44	-0.77	-0.28	1.67	-0.61	0.52	-1.02	-0.70	0.15	-0.25
Peak-1126	0.35	0.16	0.61	0.76	1.17	-0.09	2.00	-1.45	0.73	-0.21	-0.50	0.58	-0.09	-0.15	0.18	0.22	0.38	-0.79	-0.36	1.69	-0.58	0.49	-1.05	-0.73	0.19	-0.31
Peak-2248	0.44	0.15	0.59	0.73	1.05	-0.15	1.98	-1.45	0.68	-0.26	-0.57	0.52	-0.15	-0.10	0.13	0.16	0.44	-0.76	-0.29	1.66	-0.55	0.54	-1.01	-0.69	0.15	-0.26
Peak-963	0.45	0.08	0.51	0.71	1.06	-0.20	2.02	-1.57	0.67	-0.26	-0.66	0.52	-0.20	-0.22	0.13	0.34	0.38	-0.71	-0.37	1.61	-0.37	0.46	-1.05	-0.72	0.21	-0.28
Peak-33	0.41	0.17	0.60	0.74	1.11	-0.12	2.02	-1.49	0.70	-0.21	-0.58	0.55	-0.12	-0.06	0.08	0.11	0.52	-0.74	-0.23	1.67	-0.51	0.57	-1.00	-0.67	0.13	-0.17
Peak-5869	0.24	0.33	0.76	0.89	1.25	0.05	2.03	-1.35	0.85	-0.06	-0.39	0.71	-0.05	-0.05	0.00	-0.03	0.59	-0.67	-0.17	1.63	-0.35	0.68	-0.88	-0.54	0.01	-0.13
Peak-1406	0.43	0.14	0.57	0.73	1.09	-0.15	2.03	-1.51	0.69	-0.25	-0.59	0.53	-0.15	-0.08	0.13	0.13	0.47	-0.78	-0.28	1.70	-0.53	0.56	-1.02	-0.69	0.13	-0.24
Peak-561	0.08	0.38	0.76	0.94	1.37	0.17	1.96	-1.21	0.93	-0.05	-0.39	0.79	0.17	-0.29	0.01	0.69	0.32	-0.47	-0.44	1.36	-0.02	0.43	-0.89	-0.58	0.14	-0.28
Peak-4212	0.40	0.17	0.59	0.74	1.11	-0.13	2.02	-1.50	0.70	-0.23	-0.58	0.55	-0.13	-0.03	0.07	0.10	0.51	-0.73	-0.23	1.67	-0.53	0.59	-0.97	-0.65	0.09	-0.19
Peak-241	0.19	0.22	0.80	0.79	1.12	-0.22	1.53	-0.91	0.73	-0.05	-0.08	0.63	0.22	0.58	-0.73	-0.17	1.06	-0.42	0.57	0.21	0.20	0.78	-0.24	0.41	0.52	0.73
Peak-6123	0.36	-0.43	-0.58	0.04	-0.79	0.50	-0.18	-1.06	-0.14	-0.12	-0.97	-0.11	-0.50	-0.82	0.17	1.03	-0.51	0.29	-0.49	-0.53	1.08	-0.59	0.13	0.19	-0.44	-0.23
Peak-729	0.07	0.33	0.77	0.93	1.39	0.17	1.92	-1.20	0.92	-0.05	-0.31	0.79	0.17	-0.31	0.03	0.73	0.28	-0.46	-0.49	1.33	-0.11	0.38	-0.90	-0.60	0.18	-0.33
Peak-1708	-0.01	-0.46	-0.83	-0.43	-1.16	0.33	-1.17	0.18	-0.46	-0.16	-0.45	-0.37	-0.33	-0.84	0.21	0.97	-0.69	0.37	-0.49	-0.84	1.01	-0.77	0.02	0.11	-0.47	-0.26
Peak-3311	-1.32	0.99	1.15	0.94	1.77	1.51	-0.06	1.06	1.05	1.07	1.05	1.12	1.51	-0.61	0.14	1.53	-0.17	0.30	-0.71	-0.12	0.82	-0.07	-0.32	0.17	0.15	-0.38
Peak-7115	-1.54	0.97	0.92	0.64	1.40	1.62	-0.86	1.73	0.79	1.20	1.29	0.93	1.62	-0.49	-0.48	1.71	-0.15	0.89	-0.52	-0.70	1.37	-0.14	0.25	0.30	0.07	0.09
Peak-2255	-0.91	0.76	0.85	0.91	1.70	1.06	0.58	-0.09	0.98	-0.77	0.30	1.01	1.06	-0.57	-0.40	1.73	0.07	0.52	-0.53	-0.02	1.18	0.07	-0.22	0.04	0.05	0.07
Peak-407	0.02	0.17	0.37	0.98	1.02	0.04	1.70	-1.44	0.96	-0.22	-0.70	0.85	0.04	-0.53	0.15	1.24	0.09	0.13	-0.44	0.41	1.04	0.15	-0.40	0.22	0.10	0.14
Peak-3537	-1.60	1.01	0.89	0.65	1.39	1.61	-0.82	1.62	0.80	1.24	1.28	0.95	1.61	-0.49	-0.56	1.76	0.13	0.99	-0.48	-0.82	1.45	-0.15	0.33	0.37	0.11	0.01
Peak-5907	-1.58	1.00	0.89	0.65	1.40	1.60	-0.80	1.59	0.80	1.22	1.25	0.95	1.60	-0.47	-0.55	1.76	0.12	0.98	-0.48	-0.80	1.45	-0.12	0.34	0.38	0.12	0.03
Peak-5533	-1.42	0.62	0.53	-0.03	1.12	1.34	-1.22	1.29	-0.19	1.08	1.40	0.44	1.34	0.24	-0.93	1.13	0.19	1.55	0.30	-1.78	1.65	0.07	1.28	1.14	0.60	0.62
Peak-1085	-0.71	0.62	1.01	1.04	1.84	0.83	1.32	-0.68	1.13	-0.72	0.55	1.14	0.83	0.94	-1.60	0.63	1.52	1.32	0.97	-0.36	1.32	1.20	0.77	0.99	0.98	1.31
Peak-362	0.65	-0.73	-0.45	-0.16	-0.57	-0.66	0.49	-0.40	-0.01	-0.67	-0.61	-0.24	-0.66	-0.56	0.79	-0.39	-0.69	-0.70	-0.70	0.29	-0.53	-0.32	-0.11	-0.15	-0.01	-0.72
Peak-3845	-1.59	1.06	1.07	0.70	1.76	1.58	-0.20	0.82	0.91	1.39	1.35	1.10	1.58	0.62	-1.28	0.92	0.67	1.67	0.60	-1.53	1.80	0.51	1.34	1.30	0.90	0.95
Peak-194	-1.19	0.92	0.14	-0.56	0.06	0.58	0.02	0.82	0.72	0.85	0.46	0.75	0.58	1.02	-0.88	-0.46	0.69	0.83	1.04	-0.76	0.90	0.89	1.26	1.17	1.09	0.81
Peak-1220	-0.47	0.72	0.49	1.45	-0.10	0.23	0.96	-0.17	1.32	0.71	-0.21	1.17	0.23	1.69	-0.84	-1.54	1.33	0.44	1.57	-0.10	0.60	1.64	1.40	1.40	1.61	1.06
Peak-4487	-0.94	0.64	0.98	1.01	1.85	0.97	1.00	-0.38	1.13	0.88	0.80	1.17	0.97	0.93	-1.73	0.80	1.45	1.62	0.99	-0.82	1.63	1.12	1.07	1.23	1.08	1.40
Peak-1447	-1.00	0.56	0.82	0.83	1.71	0.96	0.70	-0.27	0.97	0.90	0.88	1.06	0.96	0.85	-1.74	0.89	1.39	1.71	0.96	-0.98	1.63	1.02	1.13	1.25	1.05	1.41
Peak-2577	-0.99	0.62	0.93	0.96	1.84	1.00	0.91	-0.34	1.09	0.90	0.84	1.15	1.00	0.97	-1.76	0.79	1.44	1.69	1.03	-0.94	1.73	1.13	1.18	1.32	1.15	1.42
Peak-4922	-0.23	0.09	-0.09	0.90	-0.27	0.19	0.81	-0.30	0.82	-0.17	-0.36	0.63	-0.19	1.36	-0.06	-1.87	0.71	-0.46	0.98	0.65	0.22	1.26	0.68	0.72	1.13	0.30
Peak-2450	-1.23	1.20	1.04	-0.59	2.02	1.39	0.38	0.44	0.79	0.86	0.97	0.91	1.39	0.92	-1.14	0.62	0.89	1.04	0.89	-0.51	1.02	1.06	0.98	1.03	1.02	0.82
Peak-4570	-1.45	1.71	1.55	1.21	1.73	1.61	0.59	0.53	1.32	1.49	1.16	1.46	1.61	1.56	-0.94	-0.66	1.39	0.73	1.47	-0.16	0.84	1.60	1.19	1.20	1.33	1.01
Peak-5474	0.48	0.06	0.35	1.14	-0.12	0.30	1.48	-0.75	0.87	-0.21	-0.68	0.58	-0.30	0.17	0.30	-1.22	-0.03	-0.86	-0.12	0.96	0.04	0.34	-0.24	-0.07	0.37	-0.14
Peak-1920	0.01	0.51	0.63	1.46	0.18	0.02	1.54	-0.46	1.26	-0.20	-0.32	1.00	0.02	1.55	-0.53	-1.75	1.30	0.11	1.36	0.61	0.26	1.69	0.94	1.03	1.48	0.83
Peak-5410	0.11	0.68	0.81	1.27	0.48	0.19	1.41	-0.05	1.18	0.28	-0.23	0.98	0.19	-0.05	-0.28	-0.40	0.42	0.13	-0.06	0.60	0.38	0.53	-0.01	0.21	0.52	0.18
Peak-7635	-0.71	1.30	1.35	1.67	0.74	0.79	1.33	0.33	1.59	0.97	0.54	1.47	0.79	1.12	-0.63	-1.78	1.15	0.03	0.91	0.55	0.16	1.22	0.60	0.78	1.16	0.81
Peak-4512	-0.86	1.47	1.44	1.68	0.89	1.05	0.97	0.19	1.58	1.25	0.55	1.56	1.05	1.73	-0.84	-1.44	1.49	0.37	1.64	0.14	0.58	1.75	1.21	1.25	1.52	1.10
Peak-2209	-0.40	1.00	1.00	1.63	0.58	0.46	1.59	-0.38	1.50	0.70	-0.09	1.34	0.46	0.55	-0.45	-0.90	0.67	0.00	0.34	0.42	0.65	0.73	-0.25	0.44	0.72	0.47
Peak-2520	-1.04	0.80	1.01	0.74	0.76	1.21	-0.53	2.00	0.80	1.05	1.22	0.89	1.21	0.72	-0.42	-0.93	0.54	-0.31	0.76	-0.30	0.36	0.52	0.64	0.58	0.57	0.55
Peak-5601	0.88	-0.88	-0.69	-1.15	-0.97	-0.57	-1.36	0.24	-1.23	-0.73	-0.28	-1.15	-0.57	-0.81	0.24	0.96	-0.44	0.15	-0.41	-0.40	-0.32	-0.82	0.44	-0.54	-0.82	-0.16
Peak-2054	0.07	0.24	0.60	0.60	1.39	-0.27	1.34	-0.76	0.58	-0.22	-0.11	0.46	0.27	0.87	-1.20	0.20	1.41	0.60	0.91	0.47	0.99	1.44	0.44	0.72	1.05	1.07
Peak-585	-0.14	-0.39	-0.34	-0.74	0.17	-0.16	-0.60	0.22	-0.63	-0.08	0.36	-0.51	0.16	0.74	-0.37	0.10	0.43	0.32	0.55	0.05	-0.07	0.50	0.24	-0.24	0.25	0.31
Peak-7964	0.57	0.06	0.13	0.91	-0.17	-0.48	1.50	-0.74	0.69	-0.49	-0.76	0.36	-0.48	0.45	0.05	-1.32	0.26	-0.79	0.24	0.96	0.06	0.57	-0.06	-0.11	0.59	0.18
Peak-2081	0.12	0.27	0.53	0.40	1.45	-0.42	0.93	-0.47	0.44	-0.14	0.10	0.37	0.42	0.88	-1.26	-0.17	1.39	0.76	0.98	0.22	1.04	1.33	0.52	0.77	1.00	1.16
Peak-887	0.18	0.04	0.22	0.28	1.05	-0.02	1.08	-0.80	0.28	-0.46	-0.26	0.16	-0.02	0.91	-1.17	-0.12	1.38	0.55	0.94	0.49	0.74	1.37	-0.38	0.66	0.98	1.10
Peak-89	0.11	0.20	0.44	0.48	1.31	-0.23	1.21	-0.90	0.46	-0.26	-0.24	0.35	0.23	0.89	-1.14	-0.23	1.32	0.55	0.89	0.47	0.97	1.40	-0.43	0.70	1.03	1.01
Peak-4311	1.10	-0.70	-0.23	0.24	-0.23	-0.81	1.22	-1.30	-0.01	-0.92	-1.00	-0.26	-0.81	-0.89	1.31	-0.25	-0.88	-1.22	-1.11	0.99	-0.90	-0.43	-0.75	-0.73	-0.44	-1.22
Peak-3294	-0.63	1.27	1.30	1.72	0.74	0.76	1.37	0.08	1.61	0.93	0.34	1.48	0.76	1.75	-1.12	-1.71	1.68	0.44	1.66	0.18	0.73	1.81	1.23	1.36	1.70	1.38
Peak-3307	-1.24	0.49	-0.03	-0.31	0.03	0.86	-1.55	1.49	-0.09	0.95	0.99	0.15	0.86	-0.42	-0.43	1.19	-0.61	1.29	-0.29	-1.94	0.83	-0.85	0.80	0.57	-0.14	0.08
Peak-7674	-1.23	0.48	0.01	-0.28	0.05	0.89	-1.61	1.52	-0.09	0.95	0.99	0.15	0.89	-0.47	-0.21	1.13	-0.77	1.08	-0.42	-1.79	0.71	-0.92	0.67	0.43	-0.23	-0.14
Peak-3159	-1.30	1.40	1.06	1.35	0.62	1.14	0.14	0.60	1.37	1.45	0.72	1.43	1.14	1.37	-1.17	-0.90	1.25	1.18	1.45	-1.05	1.47	1.24	1.72	1.65	1.51	1.31
Peak-7224	-1.29	0.54	0.06	-0.19	0.12	0.91	-1.48	1.44	-0.02	1.01	1.02	0.25	0.91	-0.45	-0.32	1.12	-0.65	1.17	-0.36	-1.81	0.84	-0.83	0.74	0.51	-0.15	-0.06
Peak-2530	-0.73	1.16	1.08	1.65	0.63	0.69	1.30	-0.05	1.56	1.02	0.28	1.47	0.69	1.53	-0.88	-1.34	1.24	0.44	1.32	-0.06	0.50	1.51	1.28	1.33	1.57	1.00
Peak-3405	-1.73	1.89	1.90	1.28	1.67	1.98	-0.03	1.73	1.40	1.73	1.85	1.56	1.98	1.50	-1.84	-0.44	1.67	1.66	1.68	-1.24	1.71	1.36	1.75	1.78	1.59	1.89
Peak-482	-1.69	1.90	1.94	1.26	1.67	1.96	0.00	1.69	1.37	1.71	1.88	1.54	1.96	1.45	-1.80	-0.41	1.65	1.64	1.64	-1.22	1.71	1.34	1.73	1.75	1.56	1.85
Peak-3958	-0.31	0.77	0.93	0.63	1.17	0.61	0.92	0.30	0.64	0.12	0.25	0.56	0.61	0.67	0.45	-1.10	0.22	-0.83	0.22	1.02	0.57	0.95	-0.14	-0.22	0.69	-0.36
Peak-3895	-0.38	0.90	1.05	0.72	1.21	0.71	0.89	0.34	0.73	0.24	0.33	0.66	0.71	0.62	0.51	-1.08	-0.15	-0.91	0.14	1.10	0.63	0.88	-0.03	-0.12	0.62	-0.42
Peak-4670	1.76	-1.60	-1.54	-1.06	-1.28	-1.82	0.45	-1.63	-1.20	-1.83	-1.87	-1.42	-1.82	-1.08	1.91	-0.13	-1.44	-2.01	-1.44	1.74	-1.35	-0.82	-1.64	-1.62	-1.18	-1.88
Peak-1042	1.54	-1.59	-1.72	-1.34	-1.75	-1.67	-0.56	-1.03	-1.47	-1.60	-1.60	-1.60	-1.67	-1.42	1.93	0.15	-1.89	-1.56	-1.59	0.77	-1.49	-1.50	-1.39	-1.54	-1.49	-1.90
Peak-7307	-1.32	1.08	0.53	-0.46	0.31	0.99	-0.45	1.68	0.63	0.93	1.02	0.68	0.99	1.03	-1.27	-0.64	1.06	1.06	1.26	-0.87	2.07	0.87	1.12	1.16	1.02	1.35
Peak-7797	1.73	-1.52	-1.52	-1.06	-1.30	-1.77	0.38	-1.61	-1.21	-1.78	-1.86	-1.42	-1.77	-1.09	1.88	-0.15	-1.51	-1.90	-1.43	1.52	-1.39	-0.94	-1.56	-1.57	-1.22	-1.87
Peak-6341	1.68	-1.63	-1.69	-1.29	-1.68	-1.78	-0.23	-1.25	-1.43	-1.72	-1.75	-1.60	-1.78	-1.32	1.99	0.01	-1.79	-1.81	-1.57	1.15	-1.50	-1.31	-1.53	-1.62	-1.42	-1.94
Peak-3224	-1.48	1.63	1.37	1.23	1.64	1.45	0.80	0.52	1.36	1.31	1.11	1.43	1.45	0.72	-0.83	0.42	0.69	0.84	0.58	-0.42	1.34	0.84	0.77	0.82	0.80	0.55
Peak-1477	1.77	-1.56	-1.55	-1.08	-1.37	-1.80	0.37	-1.55	-1.23	-1.78	-1.92	-1.44	-1.80	-1.16	1.94	-0.05	-1.59	-1.92	-1.54	1.51	-1.25	-0.98	-1.58	-1.59	-1.24	-1.95
Peak-7757	1.73	-1.64	-1.64	-1.19	-1.51	-1.82	0.10	-1.41	-1.33	-1.80	-1.81	-1.53	-1.82	-1.21	2.00	-0.08	-1.64	-1.97	-1.54	1.50	-1.51	-1.06	-1.61	-1.64	-1.30	-1.97
Peak-5865	1.45	-1.58	-1.69	-1.24	-1.35	-1.68	-0.09	-1.37	-1.32	-1.63	-1.68	-1.47	-1.68	-1.20	1.81	0.17	-1.69	-1.59	-1.53	1.03	-1.21	-1.17	-1.37	-1.45	-1.28	-1.90
Peak-6840	-1.19	0.17	-0.08	-0.14	0.50	0.76	-1.07	0.80	-0.08	0.84	0.90	0.28	0.76	0.01	-0.76	1.27	-0.20	1.51	0.00	-1.87	1.16	-0.41	0.97	0.80	-0.15	0.30
Peak-5940	1.27	-1.47	-1.62	-1.18	-1.29	-1.54	-0.17	-1.08	-1.23	-1.51	-1.51	-1.37	-1.54	-1.23	1.74	0.19	-1.66	-1.49	-1.51	0.92	-1.13	-1.16	-1.27	-1.36	-1.21	-1.84
Peak-6849	-1.17	0.15	-0.10	-0.18	0.49	0.74	-1.10	0.81	-0.05	0.83	0.90	0.25	0.74	0.02	-0.74	1.31	-0.21	1.49	-0.03	-1.84	1.15	-0.41	0.95	0.77	-0.14	0.27
Peak-3587	-1.21	0.21	-0.06	-0.14	0.51	0.78	-1.07	0.84	-0.10	0.87	0.93	0.30	0.78	0.04	-0.82	1.29	0.14	1.56	0.05	-1.92	1.24	-0.36	1.03	0.86	0.21	0.37
Peak-1084	-1.43	0.54	0.33	-0.15	0.85	1.08	-0.86	0.89	0.39	1.18	1.21	0.62	1.08	0.22	-1.19	1.32	0.24	1.87	0.34	-2.06	1.39	-0.11	1.28	1.13	0.46	0.77
Peak-7039	-1.44	0.54	0.36	-0.18	0.85	1.11	-0.93	0.93	0.41	1.20	1.24	0.64	1.11	0.25	-1.15	1.23	0.21	1.83	0.34	-2.04	1.31	-0.12	1.26	1.10	0.44	0.72
Peak-4436	-1.48	0.63	0.45	-0.25	0.93	1.16	-0.79	0.88	0.49	1.27	1.28	0.72	1.16	0.25	-1.28	1.32	0.36	1.93	0.41	-2.07	1.43	0.02	1.33	1.20	0.54	0.87
Peak-5238	1.75	-1.59	-1.53	-1.06	-1.27	-1.78	0.37	-1.56	-1.21	-1.83	-1.87	-1.43	-1.78	-1.02	1.89	-0.16	-1.42	-2.01	-1.41	1.75	-1.34	-0.79	-1.64	-1.61	-1.16	-1.87
Peak-8120	1.75	-1.43	-1.38	-0.89	-1.21	-1.75	0.63	-1.68	-1.05	-1.75	-1.92	-1.28	-1.75	-1.03	1.90	-0.15	-1.44	-1.97	-1.39	1.66	-1.41	-0.79	-1.54	-1.53	-1.11	-1.86
Peak-8065	1.74	-1.45	-1.41	-0.92	-1.19	-1.75	0.61	-1.70	-1.08	-1.76	-1.93	-1.31	-1.75	-1.08	1.92	-0.13	-1.47	-1.97	-1.43	1.64	-1.42	-0.83	-1.57	-1.56	-1.15	-1.88
Peak-3217	-1.37	0.99	0.89	-0.63	0.67	1.25	-0.51	1.52	0.78	1.23	1.45	0.90	1.25	1.11	-1.39	-0.31	1.05	1.42	1.29	-1.33	1.10	0.82	1.49	1.43	1.14	1.39
Peak-746	0.84	-0.83	-0.80	0.07	-1.00	-1.05	0.54	-0.34	-0.12	-1.08	-0.87	-0.43	-1.05	0.44	0.38	-1.13	-0.13	-0.91	0.14	0.84	0.28	0.56	-0.12	-0.17	0.62	-0.26
Peak-5021	-1.23	1.40	1.27	1.52	0.95	1.19	0.57	0.26	1.53	1.49	0.75	1.57	1.19	1.77	-1.29	-1.36	1.58	0.97	1.70	-0.59	1.29	1.56	1.56	1.59	1.63	1.49
Peak-5919	-0.59	0.50	0.11	0.98	-0.32	0.12	0.41	0.14	0.95	0.53	-0.02	0.82	0.12	1.26	-0.80	-1.22	0.90	0.42	1.17	-0.36	1.07	1.15	1.30	1.30	1.43	0.96
Peak-6043	-0.64	0.53	0.14	0.99	-0.29	0.17	0.37	0.19	0.96	0.58	0.03	0.84	0.17	1.29	-0.83	-1.19	0.93	0.45	1.21	-0.37	1.06	1.18	1.33	1.32	1.45	0.99
Peak-1402	-0.58	0.99	0.55	-0.74	0.35	0.34	1.28	0.19	0.83	0.58	0.12	0.79	0.34	0.55	0.18	-0.79	0.03	-0.11	0.09	0.02	0.48	0.38	0.50	0.44	0.53	0.22
Peak-457	-0.60	1.00	0.55	-0.75	0.36	0.36	1.23	0.22	0.84	0.59	0.14	0.79	0.36	0.56	0.19	-0.80	0.03	-0.12	0.09	0.02	0.38	0.40	0.52	0.46	0.55	0.23
Peak-4323	-1.32	1.00	0.50	-0.43	0.36	0.90	-0.27	1.37	0.63	1.03	0.98	0.73	0.90	0.35	-0.16	-0.31	0.25	0.59	0.11	-1.07	0.74	0.07	0.93	0.75	0.47	0.02
Peak-6566	-0.62	-0.31	-0.57	-0.81	-0.41	0.30	-1.96	1.36	-0.67	0.19	0.64	-0.51	0.30	-0.62	-0.09	0.89	-1.05	0.78	-0.50	-1.65	0.42	-1.12	0.46	0.18	-0.45	-0.34
Peak-2492	-0.69	-0.24	-0.55	-0.80	-0.39	0.35	-2.00	1.46	-0.64	0.24	0.66	-0.48	0.35	-0.60	-0.04	0.91	-1.01	0.79	-0.46	-1.63	0.41	-1.09	0.46	0.20	-0.43	-0.28
Peak-6441	-0.70	-0.22	-0.52	-0.78	-0.33	0.37	-1.97	1.39	-0.62	0.25	0.66	-0.46	0.37	-0.59	-0.04	0.95	-1.01	0.82	-0.48	-1.67	0.45	-1.09	0.48	0.21	-0.43	-0.31
Peak-6334	0.69	0.11	0.59	0.67	0.56	-0.20	1.53	-1.31	0.50	-0.26	-0.51	0.37	-0.20	0.50	-0.15	-0.76	1.06	-0.65	0.50	1.52	-0.61	0.99	-0.52	-0.25	0.31	0.42
Peak-888	-1.10	0.21	-0.08	-0.42	0.07	0.77	-1.94	1.59	-0.24	0.76	1.07	-0.03	0.77	-0.30	-0.40	0.74	-0.58	1.22	-0.05	-1.99	0.60	-0.87	0.84	0.57	-0.15	0.21
Peak-5372	-0.54	-0.37	-0.68	-0.87	-0.44	-0.20	-1.91	1.20	-0.72	0.07	0.46	-0.59	0.20	-0.62	0.22	0.87	-1.13	0.58	-0.59	-1.43	0.32	-1.12	0.31	0.05	-0.52	-0.49
Peak-5368	-0.53	-0.39	-0.71	-0.87	-0.46	-0.18	-1.91	1.20	-0.72	0.06	0.43	-0.59	0.18	-0.66	0.26	0.88	-1.16	0.56	-0.61	-1.42	0.32	-1.15	0.28	0.02	-0.55	-0.52
Peak-7068	-0.93	-0.02	-0.27	-0.61	0.13	0.61	-1.94	1.56	-0.43	0.48	0.93	-0.25	0.61	-0.47	-0.18	0.97	-0.84	1.01	-0.30	-1.81	0.51	-0.99	0.64	0.38	-0.29	-0.07
Peak-84	-1.36	0.55	0.32	-0.18	0.31	1.12	-1.82	1.70	-0.02	1.08	1.45	0.27	1.12	-0.19	-0.53	0.64	-0.38	1.32	0.12	-2.02	0.54	-0.77	0.90	0.64	-0.09	0.39
Peak-2059	-1.01	-0.08	-0.21	-0.57	-0.10	0.68	-1.98	1.64	-0.38	0.59	1.03	-0.18	0.68	-0.33	-0.38	0.91	-0.65	1.21	-0.13	-1.96	0.73	-0.88	0.79	0.53	-0.18	0.14
Peak-6948	-0.90	-0.05	-0.34	-0.65	-0.20	0.56	-1.98	1.54	-0.47	0.46	0.87	-0.29	0.56	-0.42	-0.26	0.94	-0.78	1.09	-0.25	-1.86	0.66	-0.94	0.70	0.44	-0.24	-0.02
Peak-6279	1.34	-0.73	-0.44	-0.21	-0.17	-1.15	1.74	-1.49	0.32	-1.31	-1.14	-0.56	-1.15	0.37	-0.30	0.57	0.58	-0.23	0.11	1.10	-0.23	0.83	-0.37	-0.13	0.31	0.06
Peak-907	-0.96	1.47	1.26	1.37	1.29	1.08	1.02	-0.26	1.37	1.13	0.53	1.40	1.08	1.47	-1.07	-0.63	1.39	0.76	1.48	-0.19	0.47	1.58	1.23	1.26	1.42	1.18
Peak-7088	-1.64	1.05	0.61	-0.23	0.48	1.21	-0.97	1.69	0.49	1.38	1.50	0.73	1.21	0.29	-1.03	0.50	0.19	1.71	0.54	-2.12	1.42	0.19	1.32	1.12	0.43	0.87
Peak-2451	-1.63	1.66	1.48	1.17	1.32	1.42	0.68	1.25	1.38	1.55	1.56	1.49	1.42	0.98	-1.61	-0.16	1.17	1.85	1.22	-1.65	1.92	0.83	1.62	1.56	1.15	1.52
Peak-503	-1.18	1.45	1.36	1.49	1.20	1.25	0.75	0.30	1.51	1.41	0.61	1.54	1.25	1.45	-0.97	-1.27	1.25	0.89	1.34	-0.63	1.00	1.19	1.31	1.30	1.26	1.12
Peak-2333	-1.09	1.04	0.71	1.33	0.32	0.75	0.40	0.31	1.34	1.20	0.47	1.32	0.75	1.54	-1.16	-1.16	1.18	0.87	1.46	-0.73	1.33	1.29	1.58	1.57	1.58	1.29
Peak-2265	-1.03	0.96	0.64	1.30	0.25	0.68	0.39	0.24	1.31	1.15	0.41	1.28	0.68	1.50	-1.15	-1.10	1.13	0.89	1.42	-0.77	1.35	1.25	1.58	1.58	1.56	1.26
Peak-1827	0.60	-1.23	-1.48	-1.26	-1.25	-0.98	-1.15	-0.06	-1.23	-0.94	-0.81	-1.25	-0.98	-1.30	1.53	0.79	-1.93	-0.92	-1.58	0.08	-0.75	-1.50	-0.93	-1.13	-1.28	-1.78
Peak-6291	0.83	-1.45	-1.58	-1.26	-1.11	-1.18	-0.71	-0.53	-1.24	-1.18	-1.07	-1.31	-1.18	-1.14	1.46	0.75	-1.83	-0.97	-1.53	0.22	-0.57	-1.32	-0.90	-1.05	-1.13	-1.76
Peak-473	0.51	-1.13	-1.41	-1.24	-1.25	-0.90	-1.21	0.09	-1.20	-0.84	-0.71	-1.20	-0.90	-1.23	1.54	0.60	-1.89	-0.94	-1.51	0.06	-0.75	-1.47	-0.88	-1.08	-1.23	-1.75
Peak-7756	0.82	-1.44	-1.59	-1.30	-1.16	-1.17	-0.77	-0.48	-1.27	-1.16	-1.05	-1.33	-1.17	-1.14	1.50	0.67	-1.84	-1.03	-1.53	0.27	-0.68	-1.33	-0.94	-1.10	-1.16	-1.78
Peak-3356	-0.78	1.25	1.37	1.73	0.80	0.83	1.26	0.33	1.64	0.98	0.71	1.52	0.83	1.16	-1.08	-1.25	1.31	0.23	1.08	0.36	0.54	1.25	0.68	0.92	1.27	1.23
Peak-2336	1.77	-1.48	-1.28	0.53	-1.94	-1.71	0.27	-1.34	-0.84	-1.29	-1.73	-1.06	-1.71	-0.43	1.48	-1.15	-0.90	-1.60	-0.78	1.27	-2.14	-0.55	-1.01	-1.05	-0.71	-1.27
Peak-1852	1.79	-1.49	-1.29	0.55	-1.96	-1.73	0.26	-1.35	-0.86	-1.31	-1.75	-1.08	-1.73	-0.45	1.50	-1.14	-0.92	-1.61	-0.80	1.26	-2.15	-0.57	-1.02	-1.06	-0.73	-1.29
Peak-39	-1.72	1.87	1.91	1.42	1.62	1.81	0.32	1.64	1.55	1.68	1.77	1.66	1.81	1.07	-1.83	-0.12	1.39	1.60	1.20	-1.14	2.26	1.04	1.40	1.52	1.33	1.66
Peak-6227	-1.37	1.30	1.25	1.19	1.09	1.20	0.72	1.00	1.35	1.30	1.28	1.38	1.20	1.28	-1.53	-0.48	1.25	1.59	1.40	-1.36	1.76	0.94	1.50	1.47	1.17	1.51
Peak-7828	-1.38	1.31	1.27	1.24	1.14	1.23	0.74	0.97	1.39	1.32	1.28	1.41	1.23	1.26	-1.51	-0.46	1.24	1.58	1.38	-1.34	1.76	0.95	1.50	1.47	1.17	1.48
Peak-7634	-1.40	1.36	1.26	1.13	1.06	1.26	0.55	1.09	1.29	1.34	1.27	1.34	1.26	1.20	-1.65	-0.17	1.26	1.77	1.38	-1.53	1.80	0.89	1.56	1.52	1.16	1.59
Peak-7699	-1.37	1.29	1.25	1.21	1.12	1.21	0.72	0.97	1.36	1.30	1.26	1.38	1.21	1.24	-1.51	-0.44	1.23	1.58	1.37	-1.35	1.80	0.93	1.47	1.45	1.14	1.49
Peak-4567	-1.48	0.82	0.40	-0.06	0.79	1.25	-1.31	1.75	0.16	0.80	1.36	0.34	1.25	0.41	-1.49	1.21	0.59	1.79	0.71	-1.77	1.02	0.22	1.29	1.21	0.71	1.24
Peak-4183	0.91	-1.15	-1.04	-0.64	-1.26	-0.94	-0.52	0.22	-0.77	-1.02	-0.66	-0.94	-0.94	-0.07	0.47	-0.61	-0.29	-0.71	-0.33	0.67	-1.34	-0.08	-0.33	-0.31	-0.05	-0.51
Peak-5775	-1.34	1.72	1.66	1.52	1.48	1.44	0.85	1.01	1.57	1.33	1.26	1.60	1.44	1.58	-1.10	-0.77	1.44	0.84	1.55	-0.33	1.04	1.66	1.41	1.41	1.52	1.18
Peak-8173	1.32	-1.16	-0.92	-0.23	-0.22	-1.31	1.43	-1.64	-0.35	-1.49	-1.54	-0.64	-1.31	0.74	0.17	-0.66	0.58	-1.03	0.28	1.84	0.03	1.19	-0.50	-0.22	0.52	0.14
Peak-6976	-0.09	-0.38	-0.86	-0.37	-1.25	0.38	-1.06	0.55	-0.38	0.09	-0.38	-0.40	-0.38	-0.32	1.00	-0.58	-1.13	-0.61	-0.56	-0.26	-0.35	-0.66	0.09	-0.13	-0.24	-0.96
Peak-3789	0.25	-0.13	-0.15	1.01	-0.47	0.45	1.20	-0.75	0.81	-0.13	-0.90	0.54	-0.45	1.63	-0.88	-1.25	1.27	0.37	1.44	0.00	0.87	1.63	1.32	1.36	1.63	1.06
Peak-640	0.22	-0.06	-0.10	1.06	-0.48	0.42	1.21	-0.73	0.86	-0.06	-0.88	0.59	-0.42	1.63	-0.88	-1.25	1.30	0.38	1.45	-0.01	0.88	1.64	1.32	1.37	1.63	1.06
Peak-7636	-1.54	1.95	1.92	1.09	1.58	1.73	0.34	2.09	1.26	1.34	1.91	1.34	1.73	0.81	-0.71	-0.27	0.80	0.89	0.86	-0.75	1.42	1.01	1.29	1.21	1.12	0.70
Peak-5590	-1.71	1.46	1.65	0.86	1.74	1.93	-0.48	1.82	1.03	1.65	2.01	1.26	1.93	0.92	-1.32	0.23	1.05	1.57	0.99	-1.29	0.94	0.80	1.39	1.34	1.03	1.12
Peak-1211	0.87	-0.95	-1.16	-0.37	-1.07	-1.19	0.32	-0.57	-0.48	-1.21	-1.06	-0.74	-1.19	-0.35	0.48	0.35	-0.79	-0.68	-0.53	0.43	-0.04	-0.19	-0.21	-0.22	0.00	-0.63
Peak-7243	-0.95	1.11	1.10	1.44	0.69	0.83	0.80	0.64	1.43	1.00	0.80	1.35	0.83	1.47	-1.38	-1.02	1.38	0.82	1.49	-0.43	1.39	1.43	1.40	1.49	1.62	1.51
Peak-5960	-1.01	1.17	1.17	1.47	0.79	0.92	0.79	0.64	1.47	1.09	0.85	1.40	0.92	1.50	-1.45	-1.02	1.45	0.88	1.54	-0.46	1.39	1.47	1.43	1.53	1.65	1.57
Peak-4673	-0.56	-0.05	-0.58	-0.82	-0.96	0.12	-1.90	1.37	-0.68	0.32	0.58	-0.50	0.12	-0.93	0.80	0.42	-1.42	-0.06	-0.79	-1.17	-0.63	-1.51	0.05	-0.38	-0.94	-0.79
Peak-7994	-0.38	0.48	0.35	0.96	0.42	0.14	1.09	-0.25	0.97	-0.36	-0.38	0.83	0.14	0.69	0.52	-1.71	0.14	-0.88	0.36	0.90	-0.25	0.69	-0.10	-0.12	0.53	-0.28
Peak-502	-1.08	1.64	1.81	1.63	1.54	1.35	1.23	0.49	1.64	1.32	1.01	1.64	1.35	1.79	-1.31	-1.42	1.92	0.70	1.83	0.06	0.79	1.88	1.25	1.37	1.63	1.58
Peak-4140	0.59	-0.80	-1.14	-0.98	-1.49	-0.90	-1.20	0.23	-1.02	-0.76	-0.68	-1.04	-0.90	-1.10	1.80	-0.34	-1.62	-1.44	-1.25	0.64	-1.05	-1.19	-1.04	-1.21	-1.15	-1.66
Peak-7250	-0.97	1.27	1.31	1.51	1.11	1.06	1.07	0.10	1.51	1.24	0.49	1.49	1.06	1.24	-1.49	-0.73	1.41	1.17	1.20	-0.70	1.53	1.14	1.25	1.39	1.35	1.47
Peak-5282	-0.10	0.61	0.71	1.56	0.32	0.10	1.71	-0.65	1.38	-0.40	-0.31	1.16	0.10	0.67	-0.16	-1.60	0.63	-0.70	0.37	1.14	0.23	0.75	-0.20	-0.07	0.59	0.38
Peak-2493	-0.37	-0.23	-0.62	-0.90	-0.98	-0.12	-2.09	1.53	-0.81	0.17	0.45	-0.65	0.12	-0.80	0.83	0.44	-1.46	-0.01	-0.79	-1.11	-0.49	-1.45	0.06	-0.38	-0.91	-0.88
Peak-893	-1.80	1.64	1.55	0.76	1.95	1.93	-0.18	1.30	1.00	1.65	1.75	1.26	1.93	0.80	-1.81	0.84	1.26	2.00	1.16	-1.54	1.50	0.71	1.27	1.29	0.86	1.60
Peak-3784	-1.78	1.55	1.47	-0.60	1.86	1.93	-0.45	1.47	0.85	1.63	1.80	1.13	1.93	0.66	-1.69	0.98	1.06	1.99	1.03	-1.65	1.39	0.50	1.20	1.18	0.68	1.46
Peak-4386	-0.34	0.98	1.30	1.65	0.81	0.60	1.57	0.05	1.50	0.64	0.35	1.32	0.60	1.68	-0.71	-1.88	1.52	0.12	1.48	0.76	0.57	1.83	0.86	1.03	1.54	1.04
Peak-7736	0.33	-0.72	-0.60	-0.06	-0.78	-0.49	-0.34	0.17	-0.06	-0.43	-0.50	-0.24	-0.49	-0.49	0.85	-0.35	-1.13	-0.73	-0.77	-0.03	-0.08	-0.65	-0.11	-0.21	-0.17	-0.90
Peak-6108	0.66	-0.36	-0.03	0.92	-0.40	-0.62	1.31	-0.70	0.66	-0.47	-0.78	-0.34	-0.62	0.53	0.05	-1.27	0.26	-0.74	0.19	0.88	0.34	0.62	-0.05	-0.22	0.68	0.10
Peak-6372	0.62	-0.31	0.03	0.96	-0.39	-0.59	1.38	-0.63	0.72	-0.42	-0.73	0.39	-0.59	0.44	0.11	-1.22	-0.19	-0.75	0.11	0.86	0.23	0.57	-0.05	-0.21	0.66	-0.03
Peak-325	-1.47	1.11	0.90	-0.19	2.16	1.44	-0.09	0.65	0.53	0.83	1.22	0.71	1.44	-0.03	-1.43	1.70	0.77	1.62	0.37	-1.09	1.62	0.41	0.77	0.86	0.55	1.00
Peak-1203	-1.46	1.10	0.89	-0.19	2.16	1.43	-0.09	0.63	0.52	0.83	1.22	0.71	1.43	-0.04	-1.42	1.69	0.76	1.61	0.36	-1.08	1.61	0.41	0.76	0.85	0.55	1.00
Peak-2448	-1.48	1.12	0.93	-0.22	2.17	1.46	-0.09	0.69	0.55	0.84	1.26	0.73	1.46	0.02	-1.47	1.65	0.82	1.63	0.42	-1.08	1.58	0.46	0.78	0.88	0.58	1.05
Peak-3744	-0.56	0.95	0.98	1.61	0.38	0.46	1.40	0.03	1.50	0.77	0.33	1.34	0.46	1.67	-0.88	-1.42	1.41	0.38	1.55	-0.07	0.45	1.69	1.30	1.33	1.61	1.10
Peak-4953	-0.61	0.95	0.97	1.60	0.45	0.50	1.36	0.07	1.50	0.76	0.36	1.34	0.50	1.65	-0.85	-1.36	1.39	0.38	1.53	-0.06	0.44	1.68	1.30	1.32	1.59	1.06
Peak-2721	-0.59	0.97	1.00	1.61	0.41	0.49	1.36	0.07	1.51	0.79	0.37	1.35	0.49	1.67	-0.87	-1.42	1.40	0.37	1.54	-0.06	0.45	1.68	1.30	1.33	1.61	1.09
Peak-3733	-0.60	0.96	1.00	1.62	0.45	0.51	1.37	0.06	1.52	0.79	0.36	1.36	0.51	1.66	-0.89	-1.41	1.41	0.39	1.54	-0.06	0.47	1.69	1.31	1.34	1.62	1.10
Peak-2395	-1.52	1.69	1.58	1.42	1.71	1.53	0.97	0.54	1.54	1.49	1.28	1.62	1.53	1.58	-1.38	-0.31	1.43	1.15	1.43	-0.58	1.23	1.56	1.46	1.50	1.52	1.26
Peak-3142	0.28	-0.35	-0.56	-0.49	-0.12	-0.40	0.26	0.21	0.40	-0.86	-0.35	-0.50	-0.40	0.40	-0.76	-0.42	0.58	0.37	0.50	0.03	0.94	0.28	-0.04	0.12	0.17	0.75
Peak-5911	0.18	-0.30	-0.54	-0.48	-0.15	-0.34	0.20	0.31	0.37	-0.80	-0.27	-0.47	-0.34	0.37	-0.80	-0.32	0.54	0.46	0.49	-0.09	0.96	0.23	-0.01	0.16	0.17	0.76
Peak-6037	0.22	-0.32	-0.50	-0.41	-0.17	-0.35	0.30	0.30	0.32	-0.83	-0.26	-0.44	-0.35	0.42	-0.86	-0.32	0.60	0.50	0.52	-0.11	1.06	0.29	-0.07	0.22	0.23	0.81

Table S9. VIPpred and correlation coefficients of components for each prediction model (continued)

	H28BY													H29BY												
Peak-ID	Ginjo-ka	Hine-ka	Nama hine-ka	Fatty acid smell	Grassy/aldehydic	Diacetyl	Sweetness	Sourness	Body	Bitterness	Harsh taste/acrid taste	Aftertaste	Overall quality	Color	Ethyl hexanoate	Isoamyl acetate	Nama hine-ka	Ethyl acetate	Grainy/Sweet aroma	Sweetness	Sourness	Body	Bitterness	Astringency	Aftertaste	Overall quality
Peak-6986	-0.94	1.31	1.35	1.69	0.69	1.01	0.83	0.47	1.59	1.30	0.70	1.57	1.01	1.93	-1.06	-1.75	1.59	0.40	1.82	-0.10	1.01	1.79	1.25	1.32	1.61	1.35
Peak-5240	-0.37	-0.19	-0.64	-1.05	-1.00	-0.05	-2.03	1.41	-0.93	0.10	0.55	-0.74	0.05	-1.26	1.17	0.76	-1.71	-0.21	-1.19	-0.93	-0.69	-1.66	0.31	-0.64	-1.14	-1.24
Peak-3964	0.17	-0.45	-0.49	-0.46	-0.45	0.49	-0.02	0.83	0.38	0.82	0.17	-0.55	-0.49	-0.87	-0.11	1.76	-0.80	0.49	-0.88	-0.79	0.69	-0.60	0.25	0.19	-0.05	-0.39
Peak-7748	0.17	-0.50	-0.87	-1.22	-1.35	-0.31	-1.99	0.93	-1.20	0.27	0.03	-1.05	-0.31	-1.29	1.47	0.52	-1.74	-0.62	-1.24	-0.48	-0.91	-1.61	-0.58	-0.89	-1.26	-1.42
Peak-6346	-0.80	-0.10	-0.35	-0.69	-0.59	0.37	-1.77	1.41	-0.50	0.55	0.85	-0.29	0.37	-0.95	0.39	0.91	-1.25	0.63	-0.73	-1.69	-0.06	-1.48	0.27	0.07	-0.78	-0.53
Peak-4198	-0.86	0.19	-0.28	-0.66	-0.55	0.45	-1.78	1.48	-0.47	0.63	0.92	-0.24	0.45	-0.89	0.42	0.77	-1.23	0.57	-0.69	-1.64	-0.15	-1.46	0.25	0.09	-0.79	-0.53
Peak-7759	-0.91	0.22	-0.24	-0.61	-0.47	0.49	-1.74	1.48	-0.41	0.67	0.96	-0.18	0.49	-0.92	0.37	0.88	-1.22	0.65	-0.71	-1.70	-0.07	-1.46	0.30	0.04	-0.76	-0.52
Peak-738	-0.57	0.97	1.36	1.15	1.35	0.81	1.38	0.69	1.15	0.38	0.94	1.01	0.81	1.15	-0.62	-0.63	1.05	0.41	1.02	0.00	1.05	1.49	1.15	1.17	1.39	0.67
Peak-3355	0.54	-0.50	-0.50	0.49	-0.65	-0.77	0.86	-0.57	0.30	-0.72	-0.76	-0.01	-0.77	0.41	0.09	-1.02	-0.03	-0.73	0.18	0.73	0.07	0.47	-0.10	-0.20	0.61	0.04
Peak-468	-0.62	0.09	-0.29	-0.74	-0.76	0.40	-1.99	1.56	-0.63	0.50	0.82	-0.41	0.40	-0.49	-0.08	0.63	-0.78	0.87	-0.20	-1.76	-0.12	-1.15	0.49	0.19	-0.50	-0.05
Peak-7691	-0.18	-0.40	-0.48	-0.03	0.20	0.26	-0.05	0.55	0.09	0.38	0.14	0.08	-0.26	1.09	-0.61	-0.57	0.85	0.31	0.94	-0.02	0.19	1.24	1.00	1.00	1.22	0.57
Peak-7349	-0.61	0.11	-0.24	-0.72	-0.75	0.43	-2.00	1.59	-0.61	0.53	0.86	-0.39	0.43	-0.54	-0.01	0.63	-0.83	0.83	-0.26	-1.75	-0.15	-1.21	0.45	0.14	-0.56	-0.01
Peak-4812	-0.94	0.77	0.38	1.12	0.11	0.48	0.31	0.38	1.12	0.86	0.28	1.08	0.48	1.49	-1.47	-0.72	1.27	0.89	1.46	-0.49	1.69	1.18	1.12	1.23	1.28	1.48
Peak-971	-1.05	0.87	0.49	1.17	0.26	0.60	0.33	0.56	1.19	0.92	0.43	1.15	0.60	1.48	-1.53	-0.62	1.30	0.95	1.46	-0.52	1.62	1.19	1.15	1.26	1.30	1.51
Peak-6024	-0.88	1.12	0.84	1.09	0.32	0.57	0.92	0.76	1.14	0.79	0.75	1.07	0.57	1.15	-1.03	-0.40	0.97	0.97	1.21	-0.80	0.87	0.98	1.27	1.21	1.10	1.07
Peak-7393	0.34	-0.99	-1.24	-0.49	-1.10	-0.95	-0.31	-0.04	-0.46	-0.78	-0.67	-0.62	-0.95	0.00	-0.45	0.42	-0.34	0.43	-0.01	-0.73	0.95	-0.23	0.47	0.43	0.27	0.22
Peak-4446	-0.19	0.40	0.34	1.19	-0.20	0.04	0.84	0.27	1.03	0.20	-0.13	0.79	0.04	0.66	-0.46	-1.02	0.43	0.01	0.41	-0.05	0.84	0.67	0.68	0.78	1.00	0.48
Peak-4385	-0.19	0.41	0.36	1.21	-0.17	0.05	0.86	0.24	1.05	-0.20	-0.14	0.81	0.05	0.69	-0.47	-1.07	0.46	0.00	0.43	-0.09	0.83	0.70	0.68	0.79	1.02	0.49
Peak-5408	0.91	-0.48	-0.15	0.37	0.44	-0.62	1.51	-1.53	0.21	-0.90	-1.18	-0.03	-0.62	0.97	-0.28	-0.74	0.91	-0.64	0.58	1.52	0.73	1.37	-0.19	-0.13	0.80	0.30
Peak-733	0.50	-0.41	-0.29	0.03	0.59	-0.35	0.81	-0.92	0.02	-0.87	-0.69	-0.21	-0.35	1.14	-0.54	-0.90	0.77	-0.21	0.82	0.69	1.61	1.07	-0.20	0.42	0.82	0.63
Peak-1288	-0.03	0.18	0.10	1.12	-0.53	0.13	0.75	-0.17	0.90	-0.18	-0.47	0.68	-0.13	1.50	-0.66	-1.43	1.11	0.22	1.31	-0.02	0.76	1.51	1.28	1.29	1.56	0.87
Peak-5069	0.00	0.20	0.16	1.16	-0.44	0.12	0.86	-0.19	0.94	-0.15	-0.48	0.70	-0.12	1.52	-0.66	-1.43	1.12	0.20	1.32	-0.07	0.72	1.53	1.26	1.28	1.56	0.86
Peak-727	-1.19	0.43	0.36	-0.06	1.52	0.92	-0.11	1.39	0.30	0.31	1.17	0.36	0.92	0.14	-1.37	1.55	0.72	1.59	0.42	-1.10	1.99	0.54	0.87	0.93	0.65	0.93
Peak-10	-1.11	0.39	0.35	-0.01	1.40	0.85	-0.10	1.39	0.33	0.27	1.13	0.36	0.85	0.18	-1.38	1.48	0.77	1.56	0.46	-1.02	1.94	0.60	0.86	0.94	0.69	0.93
Peak-2324	-1.18	0.46	0.45	-0.11	1.67	0.97	-0.03	1.25	0.44	0.32	1.15	0.47	0.97	0.27	-1.45	1.43	0.83	1.61	0.51	-1.05	1.92	0.66	0.92	1.00	0.75	0.99
Peak-3973	-1.25	0.49	0.48	-0.08	1.70	1.02	-0.06	1.33	0.43	0.39	1.24	0.48	1.02	0.16	-1.37	1.46	0.75	1.54	0.44	-1.00	1.95	0.58	0.82	0.90	0.66	0.92
Peak-2167	0.56	-0.63	-0.27	-0.32	0.77	-0.40	0.81	-0.69	-0.27	-0.90	-0.50	-0.38	-0.40	-0.06	0.01	0.59	-0.29	-0.46	-0.29	1.27	0.51	0.65	-0.64	-0.38	0.09	-0.31
Peak-4387	-0.17	0.45	0.40	1.31	-0.04	0.05	1.10	-0.23	1.13	-0.24	-0.18	0.90	0.05	0.47	-0.41	-0.84	0.27	0.11	0.29	-0.15	0.53	0.42	0.39	0.52	0.74	0.45
Peak-1287	0.33	-0.10	-0.08	0.94	-0.78	0.44	0.89	-0.33	0.69	-0.13	-0.74	-0.42	-0.44	1.38	-0.43	-1.58	0.85	0.02	1.11	0.13	0.64	1.30	1.12	1.13	1.42	0.67
Peak-5067	0.27	-0.08	-0.10	0.94	-0.73	0.39	0.83	-0.31	0.70	-0.11	-0.71	-0.44	-0.39	1.37	-0.45	-1.59	0.86	0.01	1.10	0.12	0.67	1.31	1.13	1.15	1.44	0.68
Peak-7112	-1.06	0.85	0.83	0.63	0.82	1.27	-0.72	1.54	0.66	0.91	1.20	0.73	1.27	1.37	-0.93	-1.13	1.12	0.45	1.41	-0.15	1.42	1.13	0.84	0.91	1.03	1.19
Peak-1003	0.00	-0.08	-0.02	0.49	-0.20	0.24	0.65	0.29	0.44	-0.27	-0.07	0.21	-0.24	0.04	0.43	-1.02	-0.30	-0.74	-0.30	0.58	0.21	0.17	-0.02	-0.08	0.39	-0.43
Peak-7996	-1.14	1.22	1.44	0.77	1.12	1.48	-0.50	0.95	0.81	1.49	1.48	1.05	1.48	0.04	-1.13	0.73	0.73	1.48	0.46	-1.23	0.15	-0.02	0.60	0.59	0.14	1.01
Peak-5743	-1.26	1.09	1.22	0.39	0.90	1.48	-1.07	1.41	0.50	1.49	1.70	0.80	1.48	-0.06	-1.07	0.76	0.48	1.53	0.40	-1.55	0.17	-0.36	0.61	0.54	-0.05	0.99
Peak-2550	-1.84	1.62	1.51	0.94	1.19	1.81	-0.65	1.70	1.11	1.88	1.96	1.36	1.81	0.62	-1.56	0.29	0.86	1.99	1.02	-2.10	0.99	0.26	1.61	1.47	0.85	1.44
Peak-408	-1.83	1.69	1.58	0.99	1.18	1.83	-0.61	1.75	1.15	1.89	1.98	1.39	1.83	0.63	-1.55	0.26	0.87	1.97	1.02	-2.09	0.97	0.28	1.63	1.49	0.87	1.45
Peak-2182	-1.81	1.63	1.53	0.97	1.14	1.80	-0.65	1.71	1.13	1.88	1.95	1.37	1.80	0.63	-1.54	0.24	0.86	1.97	1.02	-2.10	0.98	0.27	1.64	1.49	0.87	1.44
Peak-964	-1.81	1.73	1.63	1.03	1.20	1.84	-0.54	1.73	1.18	1.90	1.97	1.42	1.84	0.61	-1.54	0.32	0.85	1.98	1.00	-2.09	0.98	0.26	1.62	1.47	0.86	1.43
Peak-1407	-1.83	1.66	1.55	0.96	1.16	1.81	-0.64	1.74	1.13	1.89	1.98	1.37	1.81	0.65	-1.56	0.23	0.88	1.99	1.04	-2.11	1.00	0.27	1.63	1.49	0.87	1.47
Peak-3020	-1.82	1.66	1.56	0.98	1.18	1.82	-0.62	1.73	1.14	1.89	1.98	1.39	1.82	0.64	-1.56	0.26	0.88	1.98	1.03	-2.09	1.01	0.30	1.65	1.51	0.90	1.45
Peak-3265	-1.13	0.62	0.62	0.13	0.26	1.08	-1.51	1.32	-0.23	1.26	1.30	0.50	1.08	0.39	-1.05	0.04	0.61	1.32	0.72	-1.38	0.28	-0.13	0.71	0.65	0.15	1.08
Peak-2311	-1.79	1.66	1.58	1.01	1.16	1.80	-0.57	1.69	1.17	1.89	1.95	1.40	1.80	0.63	-1.56	0.27	0.88	1.98	1.02	-2.09	1.01	0.30	1.64	1.50	0.89	1.45
Peak-240	-1.82	1.64	1.53	0.94	1.14	1.81	-0.69	1.74	1.10	1.88	1.96	1.36	1.81	0.59	-1.53	0.28	0.85	1.98	0.99	-2.10	0.95	0.23	1.60	1.45	0.83	1.44
Peak-7012	-1.84	1.65	1.52	0.92	1.16	1.81	-0.67	1.74	1.10	1.88	1.99	1.35	1.81	0.53	-1.52	0.41	0.79	2.00	0.93	-2.14	0.94	0.16	1.56	1.41	0.77	1.39
Peak-3607	-1.82	1.68	1.56	0.95	1.17	1.82	-0.62	1.76	1.12	1.89	2.01	1.37	1.82	0.62	-1.55	0.33	0.87	1.99	0.99	-2.08	0.93	0.28	1.62	1.48	0.87	1.42
Peak-4211	-1.84	1.65	1.53	0.93	1.17	1.82	-0.68	1.77	1.11	1.89	1.99	1.36	1.82	0.60	-1.53	0.25	0.84	1.98	1.00	-2.12	1.00	0.23	1.61	1.46	0.83	1.42
Peak-6356	-1.79	1.62	1.47	0.92	1.07	1.76	-0.64	1.70	1.08	1.87	1.92	1.34	1.76	0.66	-1.56	0.22	0.88	1.98	1.04	-2.11	0.98	0.26	1.63	1.48	0.86	1.46
Peak-1993	-1.81	1.67	1.57	0.99	1.16	1.81	-0.60	1.72	1.15	1.89	1.98	1.39	1.81	0.62	-1.58	0.31	0.88	2.01	1.02	-2.11	1.02	0.28	1.63	1.49	0.87	1.46
Peak-1125	-1.81	1.61	1.50	0.91	1.11	1.80	-0.73	1.76	1.08	1.88	1.96	1.33	1.80	0.64	-1.56	0.22	0.87	1.98	1.04	-2.10	1.04	0.27	1.62	1.48	0.86	1.45
Peak-7824	-1.83	1.62	1.51	0.91	1.13	1.81	-0.73	1.78	1.08	1.88	1.98	1.33	1.81	0.63	-1.55	0.24	0.85	1.99	1.03	-2.12	1.04	0.25	1.62	1.48	0.85	1.45
Peak-7702	0.37	-0.30	-0.41	0.77	-0.80	0.59	0.64	-0.76	0.53	-0.26	-0.96	-0.27	-0.59	0.69	0.19	-1.77	0.17	-0.82	0.38	0.79	0.17	0.56	-0.11	0.21	0.63	0.06
Peak-6870	0.61	-0.94	-0.89	-0.36	-0.18	-0.70	0.46	-0.87	-0.38	-1.16	-0.99	-0.59	-0.70	0.81	-0.19	-0.59	0.43	-0.59	0.43	1.13	0.77	0.90	-0.23	-0.01	0.53	0.19
Peak-732	1.21	-1.13	-1.07	-0.53	-0.28	-1.20	0.85	-1.44	-0.62	-1.55	-1.42	-0.87	-1.20	0.48	0.26	-0.51	-0.16	-1.07	0.06	1.60	0.47	0.70	-0.69	-0.43	0.19	-0.22
Peak-2000	1.23	-1.20	-1.13	-0.57	-0.31	-1.23	0.78	-1.46	-0.66	-1.57	-1.47	-0.91	-1.23	0.47	0.30	-0.56	-0.13	-1.11	0.05	1.62	0.44	0.66	-0.74	-0.48	0.15	-0.24
Peak-7358	0.90	-1.03	-1.35	-1.48	-1.45	-1.00	-1.26	-0.25	-1.49	-1.02	-0.85	-1.46	-1.00	-1.54	1.45	1.49	-1.76	-0.54	-1.56	0.25	-0.85	-1.51	-0.79	-1.04	-1.34	-1.66
Peak-1715	-0.61	1.24	1.62	1.41	1.24	0.96	1.20	0.76	1.36	0.70	0.91	1.25	0.96	-0.08	-0.25	-0.78	0.51	-0.50	0.02	1.03	0.44	0.48	-0.41	-0.10	0.34	0.31
Peak-7918	-1.00	0.72	0.37	-0.72	0.36	0.52	0.31	0.56	0.85	0.61	0.45	0.78	0.52	0.14	-0.92	0.72	0.24	1.15	0.26	-1.20	0.78	0.26	1.14	1.05	0.78	0.54
Peak-2711	0.84	-0.89	-0.78	0.30	-0.40	-0.95	0.54	-0.83	-0.41	-1.21	-0.67	-0.63	-0.95	-0.45	0.64	-0.08	-0.57	-1.12	-0.46	1.10	-0.76	-0.22	-0.83	-0.75	-0.37	-0.57
Peak-2194	-1.63	1.27	1.06	0.38	0.70	1.53	-1.16	1.71	0.58	1.67	1.80	0.88	1.53	0.05	-0.94	0.73	0.11	1.72	0.42	-2.21	0.50	-0.44	1.23	0.98	0.21	0.78
Peak-4726	-0.60	0.22	-0.24	-0.89	-0.51	0.41	-1.81	1.14	-0.72	0.47	0.78	-0.46	0.41	-1.06	0.36	1.62	-1.13	0.74	-0.84	-1.54	-0.17	-1.30	0.21	0.10	-0.78	-0.63
Peak-2537	1.11	-0.95	-0.58	-0.07	-0.50	-1.04	0.98	-0.76	-0.12	-1.22	-1.05	-0.45	-1.04	0.15	0.50	-0.78	-0.15	-1.06	-0.18	1.18	0.52	0.51	-0.27	-0.13	0.40	-0.45
Peak-1051	-1.63	1.23	0.99	0.32	0.66	1.51	-1.27	1.74	0.52	1.65	1.79	0.83	1.51	0.04	-0.87	0.83	0.00	1.69	0.32	-2.23	0.51	-0.54	1.19	0.92	-0.14	0.69
Peak-6342	-1.61	1.20	0.94	-0.28	0.57	1.48	-1.34	1.79	0.48	1.62	1.78	0.79	1.48	0.07	-0.85	0.84	0.03	1.68	0.30	-2.23	0.51	-0.56	1.17	0.90	-0.12	0.68
Peak-7761	-1.66	1.28	1.06	0.40	0.69	1.54	-1.18	1.78	0.61	1.68	1.85	0.90	1.54	0.23	-1.13	0.59	0.31	1.82	0.61	-2.24	0.73	-0.28	1.35	1.12	0.38	1.01
Peak-4142	-1.63	1.22	0.93	-0.21	0.60	1.48	-1.32	1.81	0.43	1.59	1.83	0.75	1.48	-0.14	-0.82	0.98	0.09	1.66	0.22	-2.22	0.52	-0.61	1.12	0.86	-0.07	0.62
Peak-6284	-1.41	1.81	1.80	1.33	1.90	1.60	0.89	0.86	1.46	1.35	1.40	1.54	1.60	1.44	-1.61	-0.72	1.77	1.02	1.50	-0.17	2.18	1.52	0.92	1.15	1.28	1.67
Peak-6692	-1.64	1.50	1.33	1.08	1.76	1.54	0.62	0.50	1.29	1.56	1.35	1.45	1.54	1.18	-1.08	0.04	1.07	1.14	1.07	-0.71	0.96	1.07	1.13	1.12	1.01	0.93
Peak-3223	-1.70	1.45	1.28	0.89	1.90	1.60	0.46	0.64	1.14	1.50	1.45	1.33	1.60	1.07	-1.11	0.35	0.99	1.19	0.98	-0.76	1.13	0.98	1.06	1.06	0.92	0.91
Peak-4945	0.16	-0.07	0.26	0.03	1.33	-0.30	0.49	-0.22	0.05	-0.34	0.17	0.02	0.30	0.20	-1.18	1.01	0.65	1.01	0.39	-0.58	1.30	0.48	0.60	0.74	0.65	0.93
Peak-2393	-0.35	0.65	0.48	1.46	0.06	0.12	1.34	-0.36	1.33	-0.50	-0.26	1.13	0.12	1.57	-0.67	-1.85	1.19	0.05	1.29	0.46	0.54	1.56	0.99	1.11	1.53	0.90
Peak-6306	0.56	-0.40	-0.42	0.77	-1.07	-0.78	0.86	-0.71	0.52	-0.32	-0.97	-0.24	-0.78	0.90	0.25	-1.73	0.32	-0.49	0.60	0.43	-0.16	0.87	0.65	0.61	0.92	0.02
Peak-1210	0.50	-0.70	-0.92	-0.13	-1.05	-0.89	0.10	0.04	-0.21	-0.89	-0.80	-0.46	-0.89	-0.16	0.17	0.17	-0.44	-0.29	-0.45	0.11	0.39	0.10	0.30	-0.31	0.48	-0.40
Peak-5663	-0.58	0.92	0.86	1.60	0.44	0.49	1.27	-0.44	1.49	0.97	-0.09	1.41	0.49	1.88	-1.06	-1.83	1.73	0.44	1.76	0.18	0.70	1.85	1.24	1.34	1.65	1.35
Peak-7847	-0.79	0.26	-0.24	-0.29	-0.60	0.36	-1.31	1.82	-0.17	0.30	0.75	0.13	0.36	-0.17	-0.34	0.89	-0.43	0.84	-0.22	-1.35	0.82	-0.28	0.98	0.81	-0.43	0.04
Peak-5530	-0.05	-0.34	-0.63	-0.34	-0.92	0.37	-0.66	0.94	-0.35	-0.46	-0.18	-0.47	-0.37	-0.25	0.13	0.56	-0.47	-0.05	-0.51	-0.33	0.66	-0.01	0.49	-0.44	-0.43	-0.42
Peak-540	-0.58	-0.03	-0.42	-0.02	-0.55	0.03	-0.69	1.16	0.10	0.03	0.26	0.03	0.03	0.16	-0.68	0.55	-0.02	0.79	0.18	-1.01	0.77	0.16	1.03	0.94	0.75	0.37
Peak-1866	0.14	-0.49	-0.75	-0.49	-0.89	-0.44	-0.72	0.84	-0.50	-0.63	-0.18	-0.62	-0.44	-0.37	0.16	0.78	-0.58	0.11	-0.59	-0.44	0.53	-0.15	0.47	-0.39	-0.32	-0.48
Peak-2013	0.10	-0.47	-0.74	-0.49	-0.88	-0.41	-0.78	0.85	-0.49	-0.59	-0.17	-0.61	-0.41	-0.38	0.15	0.79	-0.58	0.12	-0.59	-0.45	0.54	-0.15	0.48	-0.40	-0.32	-0.47
Peak-2592	0.27	-0.56	-0.70	-0.02	-0.53	0.65	0.19	0.04	-0.01	-0.76	-0.60	-0.25	-0.65	0.25	-0.32	0.08	-0.15	0.00	0.10	-0.17	0.48	0.63	0.54	0.60	0.84	0.07
Peak-2582	0.37	-0.63	-0.77	-0.03	-0.61	0.74	0.21	0.05	-0.08	-0.85	-0.69	-0.32	-0.74	0.18	-0.22	0.08	-0.06	0.10	0.02	0.24	0.44	0.57	0.46	0.52	0.77	-0.02
Peak-6820	-0.17	0.34	0.27	0.89	-0.29	0.02	0.65	0.42	0.76	0.07	0.04	0.54	0.02	0.35	-0.24	-0.55	0.21	-0.08	0.07	0.13	0.71	0.54	0.55	0.64	0.86	0.17
Peak-3675	-1.65	1.62	1.34	1.04	1.55	1.50	0.45	0.95	1.26	1.37	1.52	1.37	1.50	0.99	-1.48	0.01	1.37	1.39	1.26	-0.84	1.42	1.01	1.07	1.12	0.95	1.43
Peak-1002	-1.45	1.78	1.95	1.45	1.67	1.68	0.66	1.12	1.55	1.61	1.59	1.64	1.68	0.26	-1.41	0.23	0.99	1.39	0.46	-0.99	1.32	0.42	0.87	1.01	0.77	1.21
Peak-4253	-0.47	0.80	0.96	1.50	0.42	0.47	1.24	-0.02	1.36	0.67	0.32	1.20	0.47	0.71	-0.32	-1.65	0.56	-0.37	0.52	0.53	0.55	0.57	-0.15	0.34	0.66	0.58
Peak-6504	0.41	-0.45	-0.42	-0.03	-0.76	-0.59	0.19	0.51	-0.04	-0.67	-0.43	-0.28	-0.59	-0.33	0.09	0.12	-0.45	-0.12	-0.53	-0.18	0.79	-0.23	-0.19	0.22	-0.25	-0.23
Peak-2021	0.56	-0.77	-0.67	-0.13	-0.80	-0.84	0.51	-0.09	-0.04	-0.78	-0.67	-0.25	-0.84	-0.06	0.09	0.09	-0.27	0.16	-0.23	0.00	0.50	0.06	0.29	0.35	0.49	-0.03
Peak-4121	-0.54	0.85	0.61	1.51	0.13	0.34	1.15	-0.54	1.39	0.89	-0.17	1.29	0.34	1.78	-0.78	-1.55	1.39	0.32	1.65	0.10	0.51	1.70	1.24	1.25	1.51	1.04
Peak-4388	-0.17	0.63	0.42	1.34	0.06	0.05	1.41	-0.68	1.18	-0.35	-0.46	0.97	0.05	1.54	-0.39	-1.97	0.99	-0.23	1.21	0.52	0.02	1.43	0.89	0.96	1.37	0.65
Peak-8292	-1.08	1.43	1.51	1.60	0.96	1.06	1.09	0.87	1.62	1.17	1.01	1.54	1.06	1.02	-0.93	-1.39	1.07	0.36	0.91	0.00	0.91	1.04	0.86	1.02	1.23	1.03
Peak-7763	0.25	-0.92	-1.36	-1.33	-1.18	-0.62	-1.66	0.60	-1.26	-0.73	-0.34	-1.21	-0.62	-0.92	1.20	0.47	-1.68	-0.63	-1.07	-0.32	-0.42	-1.36	-0.57	-0.79	-1.04	-1.28
Peak-735	0.40	-1.02	-1.44	-1.34	-1.24	-0.75	-1.55	0.47	-1.29	-0.87	-0.50	-1.27	-0.75	-0.93	1.31	0.40	-1.71	-0.80	-1.12	-0.12	-0.47	-1.32	-0.67	-0.88	-1.05	-1.38
Peak-919	0.15	-0.26	-0.02	-0.43	-0.23	-0.35	0.71	0.60	0.40	-0.41	-0.02	0.14	-0.35	-0.52	0.08	0.09	-0.36	-0.27	-0.69	0.25	0.70	-0.15	-0.11	-0.02	-0.18	-0.31
Peak-765	-1.12	1.25	0.88	1.26	0.78	0.85	0.84	0.55	1.35	1.07	0.36	1.32	0.85	1.26	-0.89	-1.13	1.08	0.73	1.13	-0.47	1.24	1.20	1.28	1.29	1.33	0.93
Peak-1716	-1.23	1.33	1.30	1.28	0.74	1.12	0.51	1.15	1.34	1.25	1.16	1.33	1.12	0.51	-1.03	-0.18	0.64	0.98	0.45	-0.90	0.80	0.40	1.01	1.06	0.90	0.88
Peak-3163	-0.54	1.04	0.82	1.48	0.41	0.44	1.35	-0.17	1.38	0.66	0.04	1.22	0.44	0.81	-0.11	-1.76	0.41	-0.50	0.50	0.62	0.02	0.66	-0.25	0.37	0.74	0.30
Peak-3896	0.75	-1.01	-1.00	-0.58	-1.05	-1.00	-0.12	0.29	-0.64	-1.23	-0.47	-0.87	-1.00	-0.02	-0.36	0.50	-0.23	0.17	-0.12	-0.31	0.40	0.02	0.42	0.43	0.46	0.12
Peak-3962	0.77	-1.03	-1.01	-0.59	-1.04	-1.02	-0.12	0.25	-0.66	-1.25	-0.48	-0.89	-1.02	-0.04	-0.34	0.54	-0.26	0.17	-0.14	-0.32	0.37	0.00	0.41	0.42	0.43	0.10
Peak-4941	-0.50	0.73	0.63	1.40	0.09	0.34	0.99	0.15	1.27	0.58	0.01	1.09	0.34	1.56	-1.06	-1.16	1.32	0.59	1.48	-0.24	1.44	1.58	1.41	1.46	1.65	1.22
Peak-6853	-0.15	0.21	0.22	1.21	-0.56	0.24	1.04	-0.30	0.96	-0.12	-0.54	0.71	-0.24	1.51	-0.56	-1.65	1.05	0.05	1.29	0.16	0.83	1.48	1.17	1.20	1.51	0.82
Peak-4172	0.16	0.18	0.21	1.20	-0.53	0.24	1.04	-0.31	0.95	-0.09	-0.57	0.70	-0.24	1.56	-0.56	-1.69	1.09	0.05	1.33	0.18	0.80	1.51	1.17	1.20	1.52	0.84
Peak-6839	-0.92	1.31	1.59	1.45	1.27	1.23	0.86	0.16	1.39	1.31	1.07	1.44	1.23	1.71	-1.07	-1.32	1.63	0.56	1.72	0.03	0.31	1.68	1.15	1.21	1.43	1.31
Peak-8200	0.15	0.17	0.19	1.20	-0.54	0.25	1.03	-0.34	0.95	-0.09	-0.58	0.70	-0.25	1.55	-0.57	-1.69	1.10	0.06	1.32	0.18	0.81	1.52	1.18	1.21	1.53	0.84
Peak-8275	-0.13	0.15	0.18	1.18	-0.51	0.23	0.99	-0.29	0.94	-0.10	-0.53	0.69	-0.23	1.54	-0.56	-1.67	1.06	0.06	1.30	0.16	0.80	1.49	1.18	1.21	1.52	0.82
Peak-4384	-0.08	0.44	0.58	1.21	0.10	0.06	1.26	0.13	1.06	-0.11	0.02	0.80	0.06	-0.04	0.15	-0.96	-0.11	-0.58	-0.27	0.57	-0.11	-0.05	-0.15	-0.01	0.28	-0.10
Peak-576	-0.91	1.34	1.72	1.00	1.54	1.41	0.52	0.92	1.02	1.11	1.45	1.12	1.41	0.14	-0.79	0.45	0.69	1.04	0.39	-0.79	1.36	0.29	0.54	0.56	0.29	0.73
Peak-1650	-1.32	1.60	1.83	1.52	1.75	1.47	1.11	0.99	1.61	1.27	1.39	1.60	1.47	1.42	-1.80	-0.45	1.71	1.40	1.52	-0.70	1.47	1.48	1.43	1.58	1.57	1.74
Peak-6109	-0.48	0.73	0.72	1.36	0.30	0.33	1.23	0.23	1.28	0.48	0.13	1.07	0.33	0.52	-0.25	-1.41	0.42	-0.29	0.30	0.38	0.44	0.53	-0.32	0.46	0.75	0.38
Peak-7704	0.98	-0.95	-0.62	-0.14	-0.82	-0.90	0.36	-0.27	-0.33	-1.04	-0.73	-0.59	-0.90	-0.97	1.16	0.03	-1.12	-0.94	-1.17	0.42	-0.68	-0.68	-0.44	-0.50	-0.37	-1.16
Peak-923	0.73	-0.55	-0.63	0.49	-0.95	-0.90	0.87	-0.86	-0.25	-0.67	-1.19	-0.05	-0.90	-0.06	0.63	-1.17	-0.41	-1.08	-0.30	0.89	0.00	0.05	-0.33	-0.24	-0.15	-0.49
Peak-3370	0.48	-0.64	-0.71	0.09	-1.38	-0.60	-0.65	-0.18	-0.16	-0.33	-0.75	-0.31	-0.60	0.44	0.46	-1.36	-0.15	-0.39	0.30	-0.07	0.08	0.35	0.69	0.55	0.64	-0.21
Peak-7451	-0.80	0.94	1.00	1.16	0.92	0.63	1.15	0.94	1.22	0.42	0.91	1.05	0.63	0.62	-1.06	0.24	0.67	0.87	0.76	-0.69	1.24	0.79	1.12	1.13	1.11	0.95
Peak-3165	-0.37	0.62	0.57	1.33	-0.03	0.17	1.15	0.19	1.22	0.43	0.00	1.00	0.17	0.39	-0.26	-1.14	0.23	-0.21	0.16	0.25	0.54	0.34	-0.26	0.40	0.63	0.31
Peak-619	-1.15	1.32	1.24	1.16	1.32	1.27	0.59	0.35	1.21	1.40	0.85	1.35	1.27	1.45	-0.94	-0.53	1.16	0.85	1.21	-0.39	0.90	1.40	1.25	1.25	1.30	0.86
Peak-764	-0.35	0.65	0.72	1.59	0.39	0.26	1.43	-0.49	1.45	-0.59	-0.16	1.26	0.26	1.85	-0.91	-1.70	1.57	0.25	1.72	0.33	0.75	1.87	1.17	1.26	1.63	1.17
Peak-271	0.81	-0.77	-0.74	0.26	-1.03	-0.97	0.56	-0.49	-0.03	-0.89	-1.07	-0.28	-0.97	0.94	0.23	-1.70	0.41	-0.85	0.62	0.93	0.29	1.05	0.39	0.46	0.98	0.07
Peak-6216	0.71	-0.64	-0.56	0.41	-0.93	-0.86	0.65	-0.40	-0.19	-0.74	-0.94	-0.12	-0.86	1.09	0.07	-1.84	0.59	-0.74	0.78	0.88	0.38	1.16	0.49	0.57	1.08	0.25
Peak-5598	0.32	-0.22	-0.28	-0.56	-0.45	0.63	1.21	-0.29	0.50	-0.41	-0.73	0.23	-0.63	0.00	0.35	-0.74	-0.32	-0.53	-0.39	0.48	0.20	0.14	0.00	-0.06	0.32	-0.50
Peak-2396	0.66	-0.60	-0.53	0.47	-0.86	-0.81	0.69	-0.41	-0.25	-0.69	-0.93	-0.06	-0.81	1.14	0.04	-1.87	0.65	-0.72	0.84	0.90	0.37	1.22	0.51	0.60	1.12	0.29
Peak-5344	-0.31	0.47	0.32	1.29	-0.07	0.00	1.20	-0.23	1.21	-0.40	-0.29	1.00	0.00	0.25	-0.35	-0.80	0.25	0.07	-0.05	0.18	0.46	0.31	-0.25	0.40	0.59	0.31
Peak-4631	-0.76	1.20	1.26	1.67	0.74	0.78	1.26	0.44	1.58	0.87	0.62	1.43	0.78	1.51	-0.69	-1.89	1.25	0.10	1.32	0.54	0.65	1.59	0.93	1.07	1.52	0.96
Peak-1875	0.39	-0.32	-0.16	-0.58	-0.42	-0.59	1.17	-0.28	0.50	-0.35	-0.67	-0.23	-0.59	0.52	0.20	-1.30	0.09	-0.37	0.14	0.28	0.83	0.60	0.51	0.53	0.78	-0.14
Peak-5290	0.75	-0.94	-0.87	-0.25	-1.01	-1.00	0.21	0.04	-0.36	-1.11	-0.57	-0.63	-1.00	0.08	0.47	-0.18	-0.58	-0.58	-0.31	-0.20	-0.02	-0.04	0.25	-0.19	-0.38	-0.49
Peak-4757	-1.01	1.03	0.70	1.35	0.27	0.68	0.43	0.41	1.33	1.09	0.44	1.30	0.68	1.72	-1.37	-1.24	1.45	0.63	1.69	-0.14	1.07	1.43	1.11	1.21	1.40	1.50
Peak-5084	-0.63	0.68	0.49	1.23	-0.07	0.29	0.71	0.48	1.17	0.57	0.28	1.00	0.29	0.15	-0.70	0.06	0.20	0.46	0.15	-0.49	0.89	0.16	0.61	0.69	0.67	0.60
Peak-1282	-0.76	0.89	0.70	1.35	0.12	0.46	0.78	0.41	1.31	0.78	0.38	1.17	0.46	0.28	-0.85	0.12	0.36	0.61	0.31	-0.62	0.85	0.25	0.76	0.83	0.78	0.76
Peak-2342	1.36	-1.23	-1.10	0.07	-1.02	-1.36	0.76	-1.64	-0.35	-1.32	-1.66	-0.66	-1.36	0.64	0.71	-1.50	-0.06	-1.21	0.30	1.15	-0.32	0.69	-0.03	0.00	0.52	-0.40
Peak-936	0.13	-0.44	-0.36	-0.06	-0.30	0.36	0.12	0.64	0.04	0.39	0.37	0.11	-0.36	-0.17	-0.93	1.26	0.02	1.06	-0.01	-1.14	1.39	-0.06	0.79	0.78	0.50	0.55
Peak-849	0.05	-0.52	-0.42	-0.10	-0.35	0.43	0.10	0.60	0.02	-0.49	0.32	0.18	-0.43	-0.19	-0.88	1.25	-0.07	0.99	-0.05	-1.09	1.34	-0.08	0.74	0.73	0.47	0.50
Peak-279	0.30	-0.13	0.08	0.85	-0.22	0.48	1.28	-0.02	0.74	-0.39	-0.38	0.43	-0.48	0.42	-0.41	-0.82	0.35	0.25	0.21	0.48	0.87	0.54	-0.18	0.37	0.70	0.39
Peak-5157	1.09	-1.12	-1.01	0.69	-0.93	-1.09	-0.08	-0.43	-0.83	-1.40	-0.78	-1.04	-1.09	-0.98	1.43	0.24	-1.37	-1.54	-1.21	1.17	-0.98	-0.69	-1.08	-1.10	-0.74	-1.49
Peak-7312	1.14	-1.08	-0.89	-0.12	-0.77	-1.34	1.24	-1.10	-0.03	-1.21	-1.39	-0.37	-1.34	0.21	0.17	-0.95	-0.02	-0.92	-0.05	1.14	-0.21	0.36	-0.39	-0.20	0.30	-0.11
Peak-6127	0.19	-0.49	-0.60	-0.12	-0.55	0.66	0.67	-0.22	0.16	-0.37	-0.66	-0.03	-0.66	0.16	0.02	-0.46	-0.31	-0.05	-0.14	-0.33	1.02	0.08	0.29	0.27	0.22	-0.17
Peak-6672	-1.39	1.51	1.62	1.48	1.21	1.43	0.63	1.26	1.54	1.50	1.42	1.58	1.43	1.58	-1.36	-0.74	1.41	1.26	1.67	-0.98	1.36	1.38	1.64	1.59	1.47	1.51
Peak-6483	-1.42	1.53	1.62	1.49	1.19	1.45	0.60	1.33	1.54	1.52	1.43	1.59	1.45	1.60	-1.31	-0.77	1.42	1.19	1.68	-0.89	1.30	1.43	1.62	1.57	1.49	1.46
Peak-574	-1.22	1.38	1.44	1.55	0.93	1.18	0.82	1.07	1.55	1.31	1.17	1.53	1.18	1.66	-1.26	-0.96	1.40	1.07	1.69	-0.79	1.27	1.46	1.61	1.57	1.54	1.45
Peak-840	-0.57	0.72	0.62	1.10	0.44	0.32	1.16	0.42	1.11	0.35	0.21	0.91	0.32	0.23	-0.32	-0.90	0.27	0.07	-0.04	0.18	0.54	0.36	0.31	0.45	0.65	0.26
Peak-3648	0.41	-0.43	-0.57	-0.56	-0.53	-0.32	-0.73	0.00	-0.63	-0.58	-0.77	-0.67	-0.32	-0.23	0.40	0.11	-0.24	-0.22	-0.14	0.13	-1.01	-0.19	-0.23	-0.30	-0.31	-0.24
Peak-6422	-1.26	1.67	1.86	1.57	1.41	1.44	0.84	1.33	1.60	1.29	1.49	1.58	1.44	1.22	-1.23	-1.05	1.18	0.66	1.14	-0.28	1.87	1.19	1.07	1.23	1.37	1.28
Peak-3538	-1.59	1.51	1.64	1.14	1.82	1.65	0.26	1.23	1.31	1.50	1.59	1.46	1.65	0.42	-1.29	0.40	1.09	1.17	0.69	-0.58	0.78	0.69	0.69	0.80	0.68	1.08
Peak-5172	1.05	-1.23	-1.05	-0.27	-1.05	-1.25	0.40	-0.54	-0.42	-1.29	-1.03	-0.72	-1.25	-0.58	0.76	-0.34	-0.80	-1.05	-0.81	0.76	-0.56	-0.44	-0.56	-0.50	-0.20	-0.76
Peak-2452	-0.83	0.39	0.52	-0.11	1.74	0.78	0.51	0.08	0.18	-0.40	0.74	0.33	0.78	-0.36	-0.90	1.64	0.33	1.30	0.07	-0.94	1.50	0.03	0.30	0.36	0.00	0.53
Peak-2318	-0.83	0.38	0.50	-0.12	1.74	0.78	0.49	0.07	0.17	-0.40	0.72	0.32	0.78	-0.36	-0.90	1.64	0.34	1.29	0.07	-0.92	1.48	0.02	0.29	0.35	0.01	0.52
Peak-6479	-0.87	0.45	0.59	-0.04	1.79	0.85	0.51	0.12	0.24	0.46	0.77	0.39	0.85	-0.36	-0.92	1.64	0.35	1.31	0.06	-0.94	1.48	0.02	0.29	0.35	0.00	0.54
Peak-4637	0.06	0.18	0.19	1.10	-0.20	0.22	1.21	-0.20	0.94	-0.06	-0.39	0.66	-0.22	1.51	-0.52	-1.81	1.13	0.16	1.29	0.52	0.53	1.57	0.97	1.05	1.49	0.80
Peak-2374	-1.38	1.67	1.75	0.83	1.84	1.70	0.10	1.78	0.99	1.06	1.75	1.09	1.70	0.78	-0.73	0.19	0.36	0.40	0.54	-0.14	0.68	0.39	0.24	0.32	0.36	0.58
Peak-4630	0.08	0.16	0.18	1.10	-0.21	0.24	1.23	-0.22	0.94	-0.09	-0.41	0.65	-0.24	1.49	-0.49	-1.79	1.09	0.19	1.26	0.54	0.52	1.55	0.96	1.04	1.48	0.76
Peak-8201	0.20	-0.11	0.02	0.76	-0.25	0.39	1.08	0.10	0.65	-0.36	-0.32	0.36	-0.39	0.49	-0.13	-1.11	0.19	-0.44	0.26	0.48	0.62	0.57	-0.32	0.43	0.78	0.21
Peak-8291	-1.11	1.68	1.74	1.67	1.25	1.27	1.16	0.76	1.66	1.26	1.09	1.62	1.27	0.59	-0.57	-1.37	0.79	0.05	0.49	0.41	0.29	0.63	-0.22	0.43	0.70	0.72
Peak-1461	-1.74	1.67	1.54	1.05	1.66	1.62	0.30	1.90	1.29	1.32	1.73	1.37	1.62	0.66	-1.28	0.40	0.80	1.56	0.77	-1.41	2.16	0.70	1.42	1.38	1.05	1.05
Peak-7305	-1.33	1.08	1.09	-1.11	1.89	1.14	1.15	0.38	1.32	0.89	1.03	1.30	1.14	0.76	-1.38	0.13	1.23	1.06	0.97	-0.43	0.07	0.92	0.82	0.94	0.92	1.24
Peak-282	-1.49	1.84	1.82	1.44	1.35	1.58	0.61	1.70	1.54	1.47	1.60	1.58	1.58	1.19	-1.41	-0.48	1.11	1.32	1.22	-1.19	1.86	1.02	1.62	1.61	1.43	1.41
Peak-3422	-0.57	1.01	1.11	1.65	0.36	0.62	1.12	-0.05	1.49	1.06	0.31	1.42	0.62	1.76	-0.91	-1.47	1.50	0.52	1.68	-0.07	0.81	1.74	1.35	1.37	1.57	1.17
Peak-2389	-0.58	1.03	1.10	1.65	0.34	0.62	1.14	0.01	1.51	1.06	0.31	1.43	0.62	1.81	-0.95	-1.58	1.56	0.50	1.72	-0.03	0.85	1.78	1.35	1.38	1.61	1.23
Peak-3299	-0.57	1.01	1.12	1.64	0.35	0.62	1.12	0.02	1.49	1.07	0.35	1.42	0.62	1.78	-0.94	-1.51	1.55	0.53	1.69	-0.05	0.82	1.76	1.35	1.38	1.60	1.20
Peak-2331	-0.51	0.96	1.07	1.63	0.28	0.56	1.13	-0.07	1.46	1.03	0.28	1.39	0.56	1.79	-0.92	-1.53	1.53	0.49	1.70	-0.02	0.82	1.76	1.33	1.36	1.59	1.19
Peak-2648	-1.31	1.35	1.12	1.37	0.86	0.98	0.88	1.06	1.48	1.12	0.96	1.42	0.98	1.66	-1.30	-0.91	1.39	1.01	1.57	-0.64	1.81	1.58	1.62	1.63	1.68	1.34
Peak-3678	-1.43	1.43	1.20	1.36	1.02	1.11	0.79	1.23	1.49	1.18	1.13	1.46	1.11	1.64	-1.31	-0.87	1.39	1.02	1.56	-0.64	1.83	1.58	1.61	1.63	1.67	1.34
Peak-3618	-1.37	1.37	1.14	1.36	0.96	1.04	0.84	1.14	1.49	1.13	1.03	1.43	1.04	1.66	-1.34	-0.89	1.39	1.05	1.57	-0.68	1.85	1.57	1.63	1.65	1.68	1.36
Peak-4888	-1.09	1.30	1.23	1.65	0.71	1.02	0.75	0.47	1.60	1.34	0.66	1.58	1.02	1.87	-1.62	-1.64	1.81	0.83	1.86	-0.22	1.27	1.55	1.20	1.38	1.54	1.87
Peak-7142	0.38	-0.60	-0.85	-0.25	-0.78	-0.79	0.14	0.24	-0.26	-0.92	-0.69	-0.50	-0.79	-0.65	0.91	-0.29	-0.93	-1.03	-0.89	0.62	0.12	-0.47	-0.48	-0.46	-0.23	-0.92
Peak-1887	-1.12	0.93	0.60	1.17	0.28	0.65	0.31	0.80	1.23	0.98	0.60	1.19	0.65	0.95	-1.29	-0.44	0.86	0.75	0.94	-0.46	1.34	0.68	0.77	0.90	0.91	1.20
Peak-8283	0.26	-0.28	-0.22	-0.64	-0.37	0.55	1.13	-0.34	0.55	-0.34	-0.61	-0.28	-0.55	-0.04	0.20	-0.78	-0.26	-0.46	-0.34	0.37	0.46	0.04	-0.02	-0.07	0.30	-0.27
Peak-8225	0.26	-0.30	-0.25	-0.60	-0.39	0.55	1.08	-0.30	0.52	-0.37	-0.61	-0.25	-0.55	-0.11	0.23	-0.73	-0.32	-0.48	-0.41	0.37	0.45	0.03	-0.07	-0.02	0.23	-0.31
Peak-8100	-0.66	0.95	0.93	1.49	0.58	0.54	1.28	0.30	1.43	0.65	0.38	1.25	0.54	1.38	-0.66	-1.30	1.01	0.19	1.11	0.16	1.03	1.48	1.13	1.20	1.51	0.72
Peak-6052	-0.03	0.06	-0.10	0.95	-0.46	0.30	0.87	-0.41	0.78	-0.05	-0.50	0.53	-0.30	1.01	-0.37	-1.52	0.61	0.25	0.80	0.35	0.49	0.98	0.73	0.82	1.17	0.57
Peak-1281	-1.07	1.57	1.62	1.58	0.89	1.20	0.76	1.24	1.54	1.12	1.16	1.46	1.20	0.86	-0.84	-1.04	0.86	0.20	0.78	-0.08	1.11	0.95	0.76	0.93	1.17	0.93
Peak-4252	-1.33	1.85	2.03	1.39	1.44	1.65	0.44	1.65	1.44	1.41	1.70	1.50	1.65	0.70	-1.20	-0.57	1.07	0.65	0.77	-0.22	1.38	0.83	0.72	0.92	1.02	1.21
Peak-2035	0.92	-1.16	-1.37	-0.66	-1.21	-1.29	0.11	-0.40	-0.71	-1.31	-1.22	-0.95	-1.29	-0.91	1.28	0.25	-1.48	-1.09	-1.25	0.53	-0.27	-0.78	-0.64	-0.73	-0.58	-1.48
Peak-7968	-0.68	0.99	0.89	1.58	0.57	0.54	1.33	-0.19	1.53	0.95	-0.01	1.43	0.54	1.95	-1.06	-1.78	1.69	0.39	1.78	0.24	0.79	1.89	1.22	1.33	1.67	1.32
Peak-7904	-0.98	1.15	1.54	0.98	1.10	1.09	0.52	1.79	1.08	0.77	1.58	1.02	1.09	0.12	-1.14	0.46	0.64	1.05	0.29	-0.79	1.82	0.47	0.87	0.98	0.84	0.87
Peak-8035	-0.97	1.14	1.58	0.97	1.14	1.11	0.50	1.78	1.07	0.80	1.62	1.03	1.11	-0.10	-1.04	0.60	0.47	1.00	0.08	-0.78	1.71	0.25	0.68	0.80	0.64	0.74
Peak-5859	-0.94	1.09	1.54	0.90	1.10	1.07	0.48	1.79	1.01	0.76	1.61	0.97	1.07	-0.18	-0.98	0.65	0.42	0.96	0.01	-0.76	1.71	0.19	0.62	0.73	0.57	0.68
Peak-6317	0.50	-0.78	-0.73	-0.20	-0.98	-0.85	0.13	0.40	-0.25	-0.87	-0.33	-0.49	-0.85	-0.36	-0.23	0.44	-0.35	0.08	-0.43	-0.25	0.40	-0.25	0.19	0.24	-0.25	-0.01
Peak-7310	1.01	-1.02	-1.00	0.18	-1.17	-1.13	0.21	-0.57	-0.40	-1.20	-1.10	-0.69	-1.13	-0.23	0.85	-0.98	-0.64	-1.28	-0.52	1.03	-0.24	-0.17	-0.57	-0.49	-0.09	-0.70
Peak-2410	0.08	0.07	0.24	0.94	-0.16	0.03	0.52	0.17	0.85	0.22	-0.12	0.68	-0.03	1.18	-1.31	-0.65	1.35	0.85	1.21	-0.31	1.11	1.36	1.21	1.33	1.47	1.29
Peak-7627	-1.80	1.80	1.65	1.37	1.41	1.67	0.31	1.85	1.53	1.58	1.77	1.61	1.67	1.47	-1.35	-0.46	1.32	1.24	1.53	-0.93	1.78	1.41	1.65	1.62	1.54	1.39
Peak-4944	-1.98	1.68	1.56	-0.88	2.15	1.87	0.20	1.45	1.20	1.50	1.87	1.37	1.87	0.27	-1.52	1.18	0.89	1.93	0.67	-1.68	1.85	0.52	1.40	1.36	0.90	1.22
Peak-495	-2.00	1.75	1.56	0.95	2.03	1.85	0.20	1.50	1.25	1.52	1.85	1.41	1.85	0.38	-1.65	1.09	0.96	1.99	0.80	-1.73	1.92	0.57	1.46	1.42	0.96	1.35
Peak-4916	1.07	-1.15	-1.20	-0.34	-1.12	-1.32	0.56	-0.85	-0.47	-1.27	-1.38	-0.76	-1.32	-0.68	1.21	-0.21	-1.24	-1.21	-1.07	0.76	-0.39	-0.54	-0.60	-0.63	-0.37	-1.33
Peak-6810	-0.29	0.85	1.37	0.97	0.68	0.71	0.70	1.25	0.87	0.30	0.98	0.73	0.71	0.55	-0.65	-0.71	0.58	0.04	0.43	0.23	1.52	0.68	-0.37	0.57	0.81	0.65
Peak-6750	-0.26	0.81	1.34	0.95	0.65	0.67	0.70	1.22	0.85	0.26	0.94	0.70	0.67	0.49	-0.63	-0.67	0.54	0.04	0.39	-0.22	1.49	0.65	-0.36	0.56	0.80	0.61
Peak-3492	-1.35	1.23	1.10	0.41	1.25	1.54	-0.67	1.10	0.56	1.31	1.32	0.81	1.54	0.65	-1.40	0.63	0.93	1.65	0.99	-1.40	1.04	0.37	0.96	0.94	0.50	1.35
Peak-5600	-1.48	1.22	1.12	0.36	1.44	1.70	-0.88	1.29	0.54	1.39	1.43	0.82	1.70	0.63	-1.41	0.68	0.90	1.73	0.97	-1.53	1.04	0.34	1.04	1.01	0.52	1.35
Peak-4241	-1.82	1.52	1.35	0.62	1.68	1.96	-0.80	1.91	0.82	1.45	1.81	1.05	1.96	0.72	-1.61	0.79	0.88	1.85	1.02	-1.65	1.36	0.45	1.24	1.21	0.74	1.44
Peak-5025	-1.51	1.76	1.74	1.42	1.28	1.61	0.14	1.69	1.48	1.48	1.61	1.54	1.61	1.67	-1.36	-1.30	1.61	0.61	1.69	0.00	0.65	1.50	1.02	1.16	1.37	1.54
Peak-7467	0.64	-0.90	-0.78	-0.33	-0.78	-0.88	-0.05	0.42	-0.37	-1.11	-0.57	-0.63	-0.88	-0.62	0.49	0.22	-0.83	-0.56	-0.83	0.25	0.65	-0.39	-0.22	-0.18	-0.04	-0.67
Peak-4170	-0.82	0.55	0.29	0.51	-0.22	0.29	0.05	1.63	0.61	0.41	0.78	0.53	0.29	1.33	-1.25	-0.68	1.10	1.00	1.31	-0.80	2.15	1.22	1.48	1.50	1.46	1.27
Peak-2660	-0.60	0.35	0.11	0.50	-0.37	0.08	0.16	1.37	0.56	0.21	0.50	0.43	0.08	1.23	-1.17	-0.60	1.00	0.92	1.18	-0.73	2.17	1.17	1.40	1.43	1.42	1.17
Peak-5855	-1.25	1.46	1.12	1.50	0.60	1.06	0.44	0.88	1.49	1.30	0.80	1.49	1.06	1.68	-1.58	-1.15	1.54	0.87	1.70	-0.35	1.38	1.44	1.23	1.36	1.49	1.68
Peak-3560	-0.15	0.22	0.00	0.85	-0.34	0.32	1.21	-0.23	0.83	-0.13	-0.29	0.63	-0.32	0.66	-0.40	-0.86	0.54	0.32	0.59	-0.11	0.75	0.83	0.75	0.75	0.87	0.37
Peak-5089	0.19	0.16	0.05	0.86	-0.55	0.51	1.37	-0.58	0.75	-0.01	-0.46	0.55	-0.51	0.58	-0.29	-0.87	0.51	0.32	0.58	-0.17	0.69	0.70	0.67	0.65	0.70	0.34
Peak-5384	-0.25	0.17	-0.06	0.78	-0.22	0.27	1.06	-0.06	0.79	-0.08	-0.19	0.59	-0.27	0.62	-0.38	-0.77	0.43	0.27	0.54	-0.09	0.72	0.80	0.74	0.74	0.88	0.31
Peak-3335	-0.20	0.14	-0.03	0.82	-0.22	0.29	1.14	-0.09	0.82	-0.06	-0.21	0.61	-0.29	0.59	-0.32	-0.86	0.40	0.18	0.48	-0.02	0.69	0.77	0.69	0.70	0.85	0.26
Peak-1454	-0.22	0.16	-0.03	0.83	-0.26	0.28	1.10	-0.04	0.82	-0.08	-0.18	0.61	-0.28	0.59	-0.31	-0.89	0.38	0.16	0.48	0.00	0.72	0.76	0.67	0.68	0.85	0.26
Peak-4606	-1.11	1.13	0.95	1.49	0.64	0.83	0.83	0.35	1.50	1.15	0.57	1.45	0.83	1.74	-1.66	-1.06	1.55	1.12	1.78	-0.72	1.37	1.46	1.56	1.62	1.65	1.76
Peak-4255	-0.17	0.17	0.24	0.72	-0.07	0.03	0.69	0.84	0.66	0.15	0.21	0.41	0.03	0.57	-0.37	-0.93	0.26	0.04	0.33	-0.01	1.14	0.53	0.61	0.69	0.88	0.39
Peak-4694	-0.95	0.91	0.75	1.43	0.47	0.65	0.83	0.31	1.42	0.95	0.34	1.32	0.65	1.54	-1.56	-0.88	1.34	1.07	1.57	-0.74	1.26	1.30	1.49	1.54	1.56	1.60
Peak-2785	-0.96	0.92	0.75	1.40	0.47	0.63	0.85	0.37	1.40	0.92	0.40	1.29	0.63	1.51	-1.52	-0.92	1.29	1.04	1.54	-0.74	1.32	1.25	1.47	1.52	1.54	1.58
Peak-5854	-0.95	0.88	0.75	1.41	0.52	0.64	0.86	0.34	1.41	0.90	0.40	1.30	0.64	1.52	-1.55	-0.86	1.32	1.07	1.53	-0.75	1.37	1.29	1.49	1.55	1.56	1.58
Peak-6176	-0.80	1.01	1.07	1.38	0.67	0.64	1.14	0.91	1.38	0.65	0.70	1.21	0.64	0.85	-1.01	-0.77	0.70	0.56	0.77	-0.43	1.35	0.69	0.88	0.98	1.04	1.01
Peak-5080	-1.26	1.42	1.31	1.35	0.78	1.09	0.62	1.59	1.42	1.05	1.24	1.34	1.09	0.26	-0.76	-0.21	0.15	0.66	0.17	-0.79	1.41	0.05	0.69	0.74	0.60	0.60
Peak-7905	-1.38	1.67	1.51	1.53	1.06	1.28	0.74	1.35	1.60	1.31	1.23	1.58	1.28	1.03	-1.24	-0.78	1.00	0.91	0.93	-0.66	1.56	1.00	1.28	1.36	1.38	1.15
Peak-4742	-1.55	1.50	1.31	1.34	1.16	1.43	0.11	1.03	1.44	1.57	1.21	1.55	1.43	1.11	-1.54	-0.51	1.30	1.08	1.43	-0.63	0.72	0.85	0.87	0.96	0.88	1.63
Peak-2323	-1.53	1.50	1.31	1.35	1.14	1.41	0.12	1.02	1.44	1.56	1.20	1.55	1.41	1.12	-1.54	-0.54	1.31	1.07	1.44	-0.61	0.71	0.86	0.86	0.96	0.88	1.64
Peak-6751	0.12	-0.36	-0.35	-0.07	-0.47	-0.48	0.27	0.83	0.09	-0.60	-0.13	-0.14	-0.48	-0.04	-0.31	0.08	-0.17	0.13	-0.19	-0.25	1.54	0.05	0.35	0.43	0.46	0.11
Peak-4132	-1.25	1.68	1.70	1.57	1.28	1.39	0.80	0.80	1.59	1.48	1.24	1.65	1.39	1.70	-1.24	-1.03	1.62	0.94	1.73	-0.44	1.38	1.72	1.50	1.52	1.60	1.41
Peak-5338	-1.27	1.68	1.72	1.57	1.33	1.41	0.81	0.81	1.60	1.49	1.26	1.66	1.41	1.70	-1.29	-0.99	1.65	1.01	1.75	-0.49	1.38	1.71	1.52	1.54	1.61	1.45
Peak-3627	0.49	-0.49	-0.49	-0.35	-0.60	-0.81	1.09	-0.29	0.28	-0.69	-0.69	-0.02	-0.81	0.51	0.21	-1.08	-0.11	-0.41	0.14	0.22	0.39	0.49	0.53	0.51	0.75	-0.20
Peak-3758	0.45	-0.46	-0.48	-0.35	-0.59	-0.79	1.09	-0.28	0.29	-0.66	-0.68	-0.01	-0.79	0.50	0.22	-1.04	-0.12	-0.44	0.13	0.27	0.36	0.50	0.50	0.48	0.74	-0.22
Peak-599	-1.24	0.92	0.77	1.14	0.59	0.84	0.33	1.19	1.23	0.94	0.95	1.17	0.84	1.21	-1.45	-0.64	1.05	0.98	1.23	-0.75	1.55	0.99	1.28	1.36	1.34	1.40
Peak-752	-1.24	0.90	0.73	1.14	0.57	0.82	0.32	1.14	1.23	0.94	0.92	1.16	0.82	1.24	-1.48	-0.64	1.08	1.00	1.26	-0.75	1.56	1.01	1.29	1.37	1.35	1.44
Peak-6768	1.21	-1.16	-0.85	-0.31	-0.98	-1.23	0.60	-0.30	-0.46	-1.35	-0.92	-0.76	-1.23	-0.36	1.32	-0.85	-0.92	-1.44	-0.81	1.04	-0.55	-0.18	-0.49	-0.51	-0.12	-1.22
Peak-6413	-1.51	1.23	1.24	0.96	0.90	1.37	-0.19	1.87	1.10	1.29	1.63	1.16	1.37	0.93	-1.49	-0.10	1.27	1.50	1.18	-1.12	2.10	1.10	1.45	1.47	1.27	1.36
Peak-1149	-1.10	1.37	1.78	1.04	1.87	1.51	0.64	1.00	1.12	1.14	1.65	1.22	1.51	1.26	-0.68	-0.82	0.78	0.57	1.01	-0.46	-0.05	0.67	0.79	0.78	0.72	0.86
Peak-2719	-1.11	1.38	1.78	1.05	1.89	1.53	0.65	1.01	1.14	1.15	1.66	1.23	1.53	1.26	-0.69	-0.79	0.78	0.57	1.01	-0.46	-0.07	0.68	0.79	0.78	0.72	0.86

Table S10. The top VIPpred variables for each prediction

BY	Sensory attributes	Peak-ID	VIPpred & correlation coefficients	Retention Time	m/z	Candidate compounds	Difference in Retention Time compared to the in-house library	Difference in m/z compared to the in-house library
H28BY	Ginjo-ka	Peak-495	-2.00	8.8461	71.0498	-	-	-
H28BY	Hine-ka	Peak-7636	1.95	2.0980	161.0927	-	-	-
H28BY	Nama hine-ka	Peak-4252	2.03	8.5913	217.1529	-	-	-
H28BY	Fatty acid smell	Peak-3356	1.73	1.9815	205.1167	-	-	-
H28BY	Grassy/aldehydic	Peak-2448	2.17	2.4389	104.0678	-	-	-
H28BY	Diacetyl	Peak-3405	1.98	1.5171	116.0711	L-Proline	-0.0171	-0.0017
H28BY	Sweetness	Peak-2493	-2.09	2.3473	417.1578	-	-	-
H28BY	Sourness	Peak-7636	2.09	2.0980	161.0927	-	-	-
H28BY	Body	Peak-8291	1.66	8.3406	267.1322	-	-	-
H28BY	Bitterness	Peak-964	1.90	5.1050	81.0343	-	-	-
H28BY	Harsh taste/acrid taste	Peak-3607	2.01	5.1070	209.1025	alpha-Ethylglucoside	-0.007	-0.0006
H28BY	Aftertaste	Peak-39	1.66	10.5119	271.1643	-	-	-
H28BY	Overall quality	Peak-3405	1.98	1.5171	116.0711	L-Proline	-0.0171	-0.0017
H29BY	Color	Peak-7968	1.95	8.6247	262.1191	-	-	-
H29BY	Ethyl hexanoate	Peak-7757	2.00	1.5495	163.0584	Sakebiose_f/Maltose_f/Isomaltose_f/Trehalose_f/D-Cellobiose_f	-0.05 ~ -0.08	0.0001 ~ 0.0010
H29BY	Isoamyl acetate	Peak-4388	-1.97	7.7825	219.1344	-	-	-
H29BY	Nama hine-ka	Peak-5848	1.96	1.1689	243.0298	-	-	-
H29BY	Ethyl acetate	Peak-4670	-2.01	1.5331	487.1644	Sakebiose_f/Kojibiose_f/Isomaltose_f/Trehalose_f/D-Cellobiose_f	-0.03 ~ -0.07	-0.0004 ~ 0.0013
H29BY	Grainy/Sweet aroma	Peak-5848	1.94	1.1689	243.0298	-	-	-
H29BY	Sweetness	Peak-7761	-2.24	7.6156	163.0595	-	-	-
H29BY	Sourness	Peak-39	2.26	10.5119	271.1643	-	-	-
H29BY	Body	Peak-7968	1.89	8.6247	262.1191	-	-	-
H29BY	Bitterness	Peak-894	1.75	1.1671	76.0392	Glycine_Gly-Gly_f	-0.03 ~ 0.06	-0.0004 ~ -0.0009
H29BY	Astringency	Peak-3405	1.78	1.5171	116.0711	L-Proline	-0.0171	-0.0017
H29BY	Aftertaste	Peak-2213	1.73	1.1980	298.0533	-	-	-
H29BY	Overall quality	Peak-7757	-1.97	1.5495	163.0584	Sakebiose_f/Maltose_f/Isomaltose_f/Trehalose_f/D-Cellobiose_f	-0.05 ~ -0.08	0.0001 ~ 0.0010
